# An ecosystem of carbon dioxide removal reviews – part 1: direct air CO_2_ capture and storage

**DOI:** 10.1039/d5ee01732g

**Published:** 2025-10-01

**Authors:** Mijndert van der Spek, André Bardow, Chad M. Baum, Vittoria Bolongaro, Vincent Dufour-Décieux, Carla Esch, Livia Fritz, Susana Garcia, Christiane Hamann, Dianne Hondeborg, Ali Kiani, Sarah Lueck, Shrey Kalpeshkumar Patel, Shing Bo Peh, Maxwell Pisciotta, Peter Psarras, Tim Repke, Paola Alejandra Sáenz-Cavazos, Ingrid Schulte, David Shu, Qingdian Shu, Benjamin Sovacool, Jessica Strefler, Sara Vallejo Castaño, Jin-Yu Wang, Matthias Wessling, Jennifer Wilcox, John Young, Jan C. Minx

**Affiliations:** a Research Centre for Carbon Solutions, Heriot-Watt University Edinburgh UK mv103@hw.ac.uk; b Department of Mechanical and Process Engineering, ETH Zürich Zürich Switzerland; c Department of Business Development and Technology, Aarhus University Denmark; d Chemical Process Engineering AVT.CVT, RWTH Aachen University Aachen Germany; e Department for Environmental Economics and Policy, Potsdam Institute for Climate Impacts Research (PIK) Berlin Germany jan.minx@pik-potsdam.de; f Department of Management, Technology, and Economics, ETH Zürich Zürich Switzerland; g CSIRO Energy Mayfield West NSW 2304 Australia; h Department of Chemical and Biomolecular Engineering, University of Pennsylvania Philadelphia USA; i Carbon Direct New York City NY USA; j Department of Chemical Engineering, Imperial College London London UK; k Wetsus, European Centre of Excellence for Sustainable Water Technology Leeuwarden The Netherlands; l Science Policy Research Unit (SPRU), University of Sussex Business School UK; m Department of Earth and Environment, Boston University USA; n Potsdam Institute for Climate Impact Research Potsdam Germany; o DWI – Leibniz-Institute for Interactive Materials Aachen Germany; p Climeworks AG Zürich Switzerland; q Priestley Centre for Climate Futures, University of Leeds UK

## Abstract

Direct air CO_2_ capture and storage (DACCS) is a technology in an emerging portfolio for carbon dioxide removal (CDR), understood to play a critical role in stabilising our climate by offsetting residual carbon emissions and ensuring net-negative greenhouse gas emissions post reaching net-zero. Carbon dioxide removal is anticipated to gain further importance due to lacking progress on climate reduction efforts. Meanwhile, CDR, including DACCS, is transitioning from a merely scientific effort to implementation, requiring policy and decision making based on a comprehensive understanding of the scientific body of knowledge. This calls for a source of information synthesising the body of knowledge on CDR, which we set out to author and publish as a series of systematic review papers on CDR. This first review focuses on DACCS. Given the need for practical implementation, this review reports not only on DACCS technology and state of development, but also on the state-of-the-art in technoeconomic and environmental performance, policy, equity & justice, public perceptions, and monitoring, reporting, and verification, closing with the foreseen role for DACCS in future decarbonisation scenarios. The synthesis shows that direct air carbon capture and storage can only scale and overcome current challenges, such as its high cost, *via* targeted and long-term government support, including subsidies, similar to the support renewable energy received in past decades.

## Abstract 2

We review the near-complete literature on direct air capture and storage until August 2024 inclusive, discussing technology, costs, environmental impacts, socioeconomic impacts, monitoring, reporting, and verification, and uptake and scaling.

## Graphical abstract

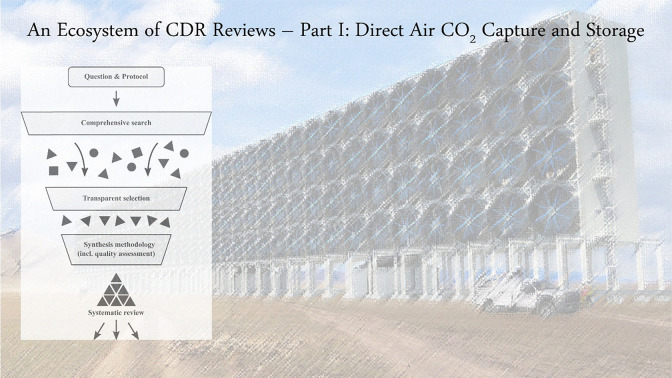

**Broader context**

Carbon dioxide removal (CDR) is a suite of approaches to remove CO_2_ from our atmosphere. This is needed to balance greenhouse gas emissions we cannot easily mitigate (*e.g.*, from agriculture) and to remove emissions from the atmosphere that were emitted previously. Humanity has started decarbonising their economies to reach the Paris climate targets, but the pace of decarbonisation is too slow, meaning we still emit too much carbon. CDR is hence becoming an increasingly important strategy to achieve climate goals, or at least not overshoot them by too much. How much carbon dioxide removal we need depends on the future pace of decarbonisation, and is the subject of many scenario studies, for the world, but also for countries and regions. These studies need quality scientific data inputs on CDR potentials, costs, environmental and socioeconomic side effects, and possible incentive policies. To provide a synthesis of the data for CDR strategies, a team of global scholars set out to review the literature on CDR and collect as much information as possible. This is the first publication resulting from this effort, for a CDR approach called direct air capture. A technical approach that quite literally filters CO_2_ from the air.

## 1. Introduction

Carbon dioxide removal (CDR) refers to the removal of CO_2_ molecules from the atmosphere, which is recognized as an indispensable component of any effort to meet the Paris Agreement climate targets of limiting global temperature increase to well below 2 degrees celsius, while pursuing efforts to limit the increase to 1.5 degrees. Recent science assessments have highlighted that the removal of CO_2_ complements rapid, sustained and deep emissions reductions across 1.5 °C and 2 °C decarbonisation scenarios,^1,2^ corroborated by several integrated assessment modelling studies (*e.g.*, ref. 3 and 4).

There are multiple reasons why CDR is an indispensable component of any effort to meet the Paris climate goals (*e.g.*, ref. 2 and 5): first, achieving net-zero emissions to halt global temperature rise requires compensating residual emissions that are otherwise hard-to-abate;^6^ second, many Paris-consistent scenarios involve so-called temperature overshoot, where global mean temperatures temporarily exceed the limit and are pulled back by reducing atmospheric carbon dioxide removal concentrations through net-negative emissions;^5,7^ third, beyond overshoot, scholars have suggested the need to reverse anthropogenic climate change by cleaning up the atmosphere through CDR by re-establishing pre-industrial or at least substantially lower atmospheric carbon concentration levels;^6,8^ finally, given the large climate-physical uncertainties (*e.g.*, climate sensitivity) CDR could insure against less favourable climate outcomes and climate feedbacks.^8^

The need for and dependency on CDR continues to increase due to the lagging global progress on greenhouse gas emissions mitigation, *e.g.*, *via* renewable energy, fuel switching, bioenergy use, and CO_2_ capture, utilisation, and storage, and the continued growth in global greenhouse gas emissions. Recent research suggests there is not only an emissions gap,^9^ but also a carbon dioxide removal gap,^2,10^*i.e.*, countries’ plans for CDR deployment – in the short as well as long term – are out of line with the requirements derived from long-term mitigation pathways.

To close the CDR gap, a key step is to understand the state of knowledge on CDR research and implementation. The Intergovernmental Panel on Climate Change (IPCC) Sixth Assessment Report was clear in highlighting the importance of CDR while it struggled to provide comprehensive information on the individual CDR pathways and how they compare. This shortcoming in part stems from the underlying review papers that have covered a broad spectrum of CDR methods but have remained rather coarse and unspecific in their treatment of individual methods (*e.g.*, ref. 11–15) and are no longer fit to advance the scientific and policy debates.

One CDR pathway is direct air CO_2_ capture coupled with safe, permanent storage (direct air CO_2_ capture and storage, DACCS). DACCS is the name for a suite of technological solutions that separate CO_2_ molecules from the atmosphere (air) and produce a pure CO_2_ stream that can be stored in deep underground reservoirs, mineral formations in the earth's crust, or waste and natural minerals at the earth's surface.^16–18^ These solutions rely on energy, often water, and other resources to fulfil this task, and often on a connection to CO_2_ transportation and storage infrastructure. DACCS is one of the technologies believed to be required to achieve the Paris climate targets, as part of the total portfolio of decarbonisation options.^17^

Here, we present an exhaustive review of the scientific, peer-reviewed literature on direct air CO_2_ capture and storage, plus a limited number of peer-reviewed grey literature publications. This review is part of a wider CDR literature reviewing effort, aiming to deliver an ecosystem of systematic reviews on carbon dioxide removal, undertaken by scientists from across the globe. The forthcoming review papers will cover the other land, air, and ocean-based CDR approaches, plus overarching themes like utilisation of CO_2_ to produce value-added products. There are at least three overarching goals of such a review ecosystem:

• High quality insights enabled by rigorous review methodologies: rigorous systematic review methodologies are applied to synthesize the large and vast-growing literature on CDR. This approach enables similar levels of transparency and rigour as common for primary research.

• Relevance through granularity and comprehensive scope: as CDR turns from a mainly scientific discourse into an implementation and policy discourse, the information demands are much more specific than can be met by single reviews across several CDR methods. Instead, we develop an ecosystem of systematic reviews, each carried out by a team of leading international experts on one CDR approach (*i.e.*, DACCS, afforestation, enhanced rock weathering, et cetera) assessing the information on all relevant pathways within an approach (here, the suite of direct air capture (DAC) technologies) explicitly.

• Synthetic insights through common design and protocol: to enable critical synthesis and cross-technology learning, there is a need for a consistent approach and coordination across systematic reviews. Reviews of the ecosystem therefore share a common design and follow harmonized concepts, definitions and methodologies all enshrined in their respective protocols.

This first review aspires to summarize the state of knowledge on DACCS serving a diverse range of audiences from DACCS scholars and practitioners to scientists working in the climate change and climate change mitigation domain and from IPCC and other climate change assessment authors to policymakers. Compared to other existing reviews, *e.g.* ref. 19–23, it is much broader in scope, cutting across a range of topics and scientific disciplines, highlighting the state-of-the-art in DAC engineering and physical sciences, technology assessment and appraisal, policy and public perception, monitoring, reporting, and verification, and the role of direct air capture in future climate scenarios.

The systematic review on DACCS followed four broad methodological steps:††Methodological guidance on systematic reviews can, for example, be found in the Handbooks of the coordinating bodies such as cochrane (https://training.cochrane.org/handbook/current) or the collaboration of environmental evidence (https://environmentalevidence.org/information-for-authors/). (i) development of a review protocol (supplementary MS Word file), formalising how to screen the scientific literature, which topics to include respectively exclude, how to code the data in each publication, which data to retrieve, and so forth; (ii) automated searching, identification, and classification of the scientific DACCS literature, assisted by machine learning as outlined in Lück *et al.*;^24^ ‡‡A publicly accessible, comprehensive repository of carbon dioxide removal research can be found here: https://climateliterature.org/#/project/cdrmap. (iii) data extraction and harmonization, where different reviewers were responsible for reviewing a specific technology or topic; and (iv) synthesis, analysis, and manuscript preparation. All scientific papers in the respective bibliographic databases until 31 August 31, 2024 were included, rendering a total peer-reviewed scientific literature body of over 800 manuscripts. Grey literature reports were included only if some form of peer-review had been undertaken, and when adding sufficiently important insights to the scientific body of literature.

This manuscript is structured as follows. First, the direct air DACCS literature landscape is identified and discussed, based on machine-learning-supported article selection and classification. Second, eight DAC technology categories are concisely discussed, including their working principles, challenges and opportunities, and reported energy and resource consumptions. The review continues with discussing DAC's technoeconomic performance and life cycle environmental impacts, followed by discussions on policy, equity and justice, social perceptions, and monitoring, reporting, and verification (MRV). The final section discusses upscaling and the featuring of DAC in integrated assessment modelling (IAM) scenarios, including new model results highlighting the role of DACCS in future scenarios that limits temperature rise to well below 2 °C.

## 2. The DACCS literature landscape

While the concept of direct air capture has been established in the context of air purification in spacecrafts, submarines and fossil fuel processes since the 1940s, it was introduced for climate change mitigation only in the late 1990s.^25^ As such, DACCS is a much more recent technology concept in climate change mitigation than many other CDR options such as soil carbon sequestration or afforestation and reforestation.

**Fig. 1:**
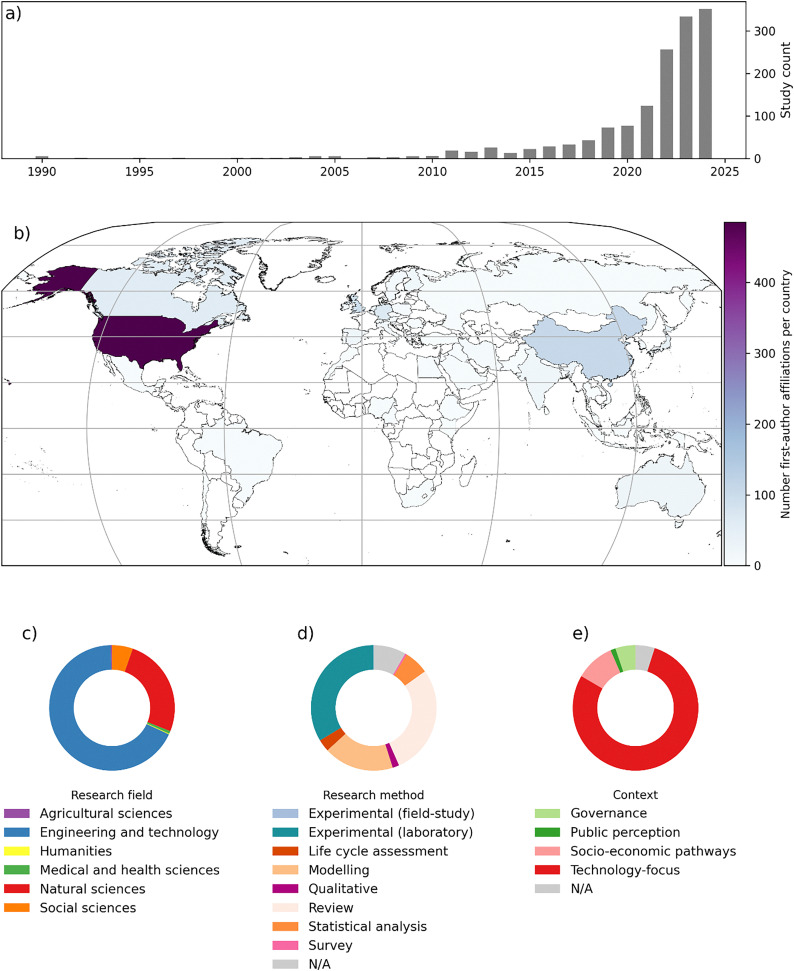
DACCS research landscape, (a) number of studies per year; (b) number of studies per country (based on first-author affiliation). (c) Share of research fields the studies were published in (based on OpenAlex field classifications); (d) share of scientific methods used in the studies; (f) share of the main context in each study. For (c)–(e) we used the most prominent category per study.

Following the methodology outlined in Lück *et al.*^24^ to OpenAlex, the largest open bibliographic database, we estimate the literature on CDR to comprise 53 000 publications for the period 1990–2024. Only 2.8% of these are primarily on DACCS, but the share has been growing in recent years from around 0.9% in 2010–2014 to 3.8% in 2020–2024, with the highest share of 4.9% (287 studies) in 2024 (Fig. 1).

CDR research is growing more rapidly than the climate change literature as a whole.^2,24^ Overall, the compound average growth rate for scientific literature on CDR was 20% for the last five years (13% for 2014–2024). Literature on DACCS has seen one of the highest growth rates with 41% in the last five years (37% for 2014–2024).

The vast majority of DACCS publications are technology studies published in engineering (67%) or natural science (25%) journals. Socio-economic aspects and public perceptions of DACCS appear to receive only little attention with 5% of studies published in social science journals. A third of all DACCS studies conduct experimental lab work, followed by 28% reviews, and 18% perform modelling analyses. Content wise, most DACCS research focuses on understanding different adsorbents and other materials and associated process designs. Integration of DACCS into energy systems as well as wider CDR portfolios is a more recent trend in the literature. In particular, we observe a recent growth in the socio-economic pathways literature, where the potential role of DACCS is explored in long-term climate change mitigation scenarios – usually as part of a broader CDR portfolio comprising afforestation/reforestation as well as bioenergy with carbon capture (BECCS) amongst others, *e.g.*, ref. 3. This observation is matched by an emerging literature on the role of renewable energy in powering DACCS systems including related integration issues.^26^ Finally, we observe important discourses around process efficiency, cost as well as governance/policy, all growing in importance in recent years.

Most research on DACCS was conducted by authors affiliated with institutions in the USA (33%), followed by China (7%), Great Britan (6%), and Germany (5%). There are large parts of the world where we currently do not find any DACCS publications, notably South America and Sub-Saharan Africa. We were unable to extract mentions of geographic locations from the study abstracts, which highlights the lack of field studies (as also indicated by the distribution of research methods) and early development stage. While DACCS is usually considered a technology that can be applied anywhere, local conditions can affect the efficiency of DACCS processes and will therefore remain an important avenue for future research.^27,28^

## 3. Technology overview

This section concisely discusses eight different technology categories under development for direct air capture:

(1) Solid adsorbents

(2) Liquid absorbents with calcium looping regeneration

(3) Liquid absorbents with electrochemical regeneration

(4) Solid adsorbents with electrochemical regeneration

(5) Amine and amino acid-based liquid absorbents with thermal regeneration

(6) Mineral looping

(7) Membrane-based DAC, and

(8) Cryogenic DAC

The section introduces major technological challenges for each category, as well as key attributes including energy consumption and state of development and deployment. Challenges and attributes are discussed as relevant to each technology and may differ across technology categories. The section also discusses fast-tracking of materials discovery and design for three DAC technology categories, using computer-based materials screening methods. We end the section by synthesising the technologies’ attributes and energy consumption. Costs, economics, and life cycle environmental performance are discussed in Section 4.

The review of DACCS technologies in this section is less comprehensive than other DAC technology reviews, commensurate our aim to provide an interdisciplinary account of the state of DAC scientific research – not merely an account of technology research. This section does introduce the most important technology elements to help the reader understand the technical context and better appreciate the subsequent sections. Excellent DAC technology reviews exist that need no repetition, examples include those by Zhu *et al.*^22^ and by Low *et al.*^19^ on adsorption-based DAC, Sharifian *et al.*^29^ on electrochemical approaches to CO_2_ capture, and Ignatusha *et al.*^30^ on membrane-based DAC.

### 3.1. Solid adsorbents

#### 3.1.1. Introduction

Adsorption-based DAC entails the physical (physisorption) or chemical (chemisorption) binding of CO_2_ to the surface of a porous adsorbent material. Subsequently, the adsorbent is regenerated, releasing the CO_2_, *via* heating, a reduction in pressure, or introducing a competitive species.^31,32^ Using high selectivity materials, for example, amine-functionalised chemisorbents, CO_2_ can be produced at >99% purity.^33^ Meanwhile, the CO_2_ is almost always delivered at 1 bar, as compressing the air feed is not energetically favourable, so post-processing steps will be used to compress the CO_2_ to the desired pressure.§§Exceptions exist.^67^ While adsorption-based DAC could be realized using many potential material and process combinations, the most mature is temperature-vacuum swing adsorption (TVSA) using amine-functionalised adsorbents. Research into using adsorbents to remove CO_2_ from ultradilute streams has a long history, and traces back to space exploration, long before DAC was considered a climate change solution.^34–37^

#### 3.1.2. Major challenges

Technical research on adsorption-based DAC is categorised into material science or process development, and both are attempting to solve the same fundamental challenges. These challenges, which feed into the overall cost, are detailed in Table 1, with examples of how each field has attempted to address the problem.

**Table 1:** Fundamental challenges virtually all technical research is aiming to address for adsorption-based DAC

Challenges	Material science example	Process development example
Productivity – maximisation of CO_2_ production rate per bulk volume of adsorbent material	Synthesising materials with a higher CO_2_ capacity.^36^	Investigation of microwave heating to reduce the time to regenerate the adsorbent.^38^
Energy consumption – minimisation of heat and electricity use per unit of CO_2_ produced	Development of humidity swing adsorbents to reduce the heat input.^32,39–42^	Heat integration, heat pumps, and waste heat use.^43,44^
Adsorbent lifetime – extension of adsorbent lifetime to reduce changeout frequency	Comparison of the stability of alumina *versus* silica materials under direct steam contacting.^45^	Adding a cooling step before exposure to air and vacuum steps before exposure to heat to protect against oxidative degradation.^46^
Ambient variability – the development of a material-process combination or a portfolio of material-process combinations that can perform across a wide range of temperatures and humidities	Development of hydrophobic moisture-swing materials so performance can be maintained at high humidities.^47^	A comparison in the performance of a specific process-material combination across the world.^48,49^

A significant issue is that the performance indicators defining the challenges above compete and trade-offs exist. To quantify these trade-offs, detailed technical cost analysis is required that assesses a proposed solution against a benchmark. Techno-economic analysis will be covered in Section 4. Table 1 contains examples of how the material science and process engineering fields have individually tried to address the major challenges. However, a holistic approach born from deep collaboration between the two fields will likely yield the best overall solutions (see Section 3.9).

#### 3.1.3. Materials & contactor design

**Fig. 2:**
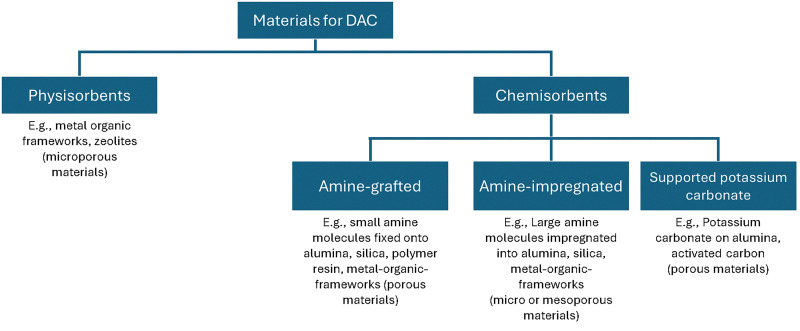
General high-level breakdown of the adsorbent materials studied for solid sorbent direct air capture.

Extensive reviews exist covering the materials space for solid sorbent DAC (Fig. 2).^19,22,50^ Thus, only the main classes of materials will be introduced here. The class of materials that has garnered the most attention in literature is amine-functionalised materials due to their high selectivity to CO_2_ over N_2_ and O_2_ and lack of competition between CO_2_ and H_2_O.^31,45,51–62^ However, they also undergo oxidative degradation, so there are questions over their lifetime.^63–65^ Meanwhile, ion-exchange humidity swing adsorbents are also highly selective to CO_2_ over N_2_ and O_2_, and additionally, they promise lower regeneration temperatures or no heat input altogether.^32,39–42^ But, these materials suffer from competitive adsorption between H_2_O and CO_2_ at high humidities, making them exclusively suited for arid environments. Physisorbents, such as zeolites, metal–organic frameworks (MOFs), or covalent-organic frameworks (COFs) could promise greater long-term stability and lower regeneration energy,^36,66–69^ while they usually suffer from either poor CO_2_ selectivity to N_2_ or to H_2_O. Finally, a very small proportion of the literature has investigated potassium carbonate-containing materials.^70,71^ These materials are selective to CO_2_ over N_2_ and O_2_ but require higher temperatures for desorption (>150 °C) and potentially exhibit slower adsorption and desorption kinetics.

As of today, there has not been a material, which has proven to be significantly better than amine-functionalised in real process conditions. However, breakthroughs in the material design could lead to a step change in the cost *via* an improvement in any one of the fundamental challenges identified in Table 1. Although, many of the alternative classes of material – including zeolites, MOFs, and COFs – appear to have fundamental challenges associated with them. For example, CO_2_ and water usually compete for the same physisorption sites, particularly under direct air capture conditions where sites with very strong binding energies, such as polar or charged sites, are required, making these materials ineffective in the presence of humidity.^72,73^ As a result, it is imperative that new material discovery is paired with smart process design and development, so that these materials can be judged in processes optimised to overcome their challenges rather than in processes designed around typical DAC adsorbents such as amine-functionalised adsorbents.^68^

Another important consideration within this literature space is the geometry utilised to contact the active material with air. Structured materials have been proposed as a promising approach to reducing pressure drop and improving mass transport compared to a packed bed, thereby reducing electricity consumption and increasing productivity.^74^ Monoliths are the most commonly researched form of structuring due to their mechanical stability, low-pressure drop, and existing knowledge from other industries.^70,74–77^ Alternatives proposed include flat-packed beds and laminates.^78–81^

#### 3.1.4. Processes and process evaluation

Adsorption processes are inherently cyclic, where a packed bed of adsorbent pellets or a structured bed undergoes a series of states relating to adsorption and regeneration. Since the adsorption mode remains the same across adsorption processes, their regeneration mechanism can be used to define the process. Regeneration mechanisms are heating (temperature swing adsorption), a pressure differential (vacuum or pressure swing adsorption), a dilute/competitive purge (*e.g.*, humidity swing adsorption), or a combination of these.

**Fig. 3:**
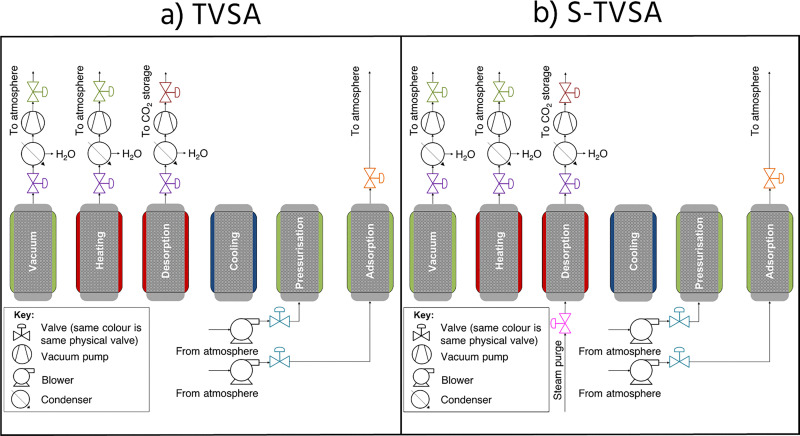
Example process cycle diagrams of (a) a TVSA, process and (b) a steam-assisted TVSA process. The humidity swing process would appear similar to the steam-assisted TVSA process, but the steam purge would be sub-ambient steam. It is important to note that these cycles serve as illustrative examples, and many permutations can be designed. Reproduced with permission from Young *et al.* 2023.^46^

In the context of direct air capture, temperature only swing adsorption may lead to low CO_2_ purities, due to the air remaining inside the column contaminating the CO_2_ product. Meanwhile, pressure/vacuum only swing adsorption will likely not provide enough energy to desorb the CO_2_, given the strong binding required to obtain meaningful CO_2_ capacities from the low concentrations in air. By far, the most commonly studied process is temperature vacuum swing adsorption, which employs a vacuum (<0.3 bar) and heat (80–120 °C) to desorb CO_2_ from the adsorbent (Fig. 3).^33,46,48,49,82,83^ The heat can be supplied *via* heat exchange between a heating fluid and the adsorbent bed, microwaves, electrical resistance, induction, or direct steam contacting.^82,84–86^ Moisture or humidity swing adsorption has also received attention as an approach for certain materials, where water adsorbs competitively over CO_2_ causing any adsorbed CO_2_ to desorb on exposure to high humidities or liquid water.^32,39–42^

To date, limited modelling work has been undertaken on DAC adsorption cycles. However, process modelling efforts are currently hampered by a critical lack of materials data and fundamental understanding of the underlying adsorption phenomena in the presence of humidity. For example, it has been identified that only two amine-functionalised materials have sufficient equilibria data for detailed modelling, whilst the same research group recently added an additional material to this list.^19,46^ Additionally, despite the importance of mass transfer in the context of DAC,^74^ no work has been performed to characterise adsorption and desorption mass transfer in ambient conditions. Generally, data availability is even more sparse for other adsorbent classes of interest for DAC.

Process modelling work has focussed on characterising and optimising cyclic adsorption processes for DAC.^46,74,82,83,85,87^ This work has mainly focused on TVSA and steam-assisted TVSA. There have also been studies to assess how performance varies by location, including novel process strategies to improve performance, such as the humidification of the air inlet.^48,49^

Whilst there is some work on modelling the adsorption cycle itself, even fewer studies analyse the DAC system outside the boundaries of the adsorption bed. Liu *et al.*^88^ have assessed the integration of a heat pump, but no work characterises the detailed balance of plant and design of other critical pieces of equipment. These pieces are essential to accurate cost estimates, and addressing these challenges in academia may lead to novel approaches to design that can drive improvements in performance and cost as we advance.

#### 3.1.5. State of development and deployed plants

Most pilot, demonstration, and commercial plants deployed to date use an adsorption process. Climeworks and Global Thermostat (acquired by Zero Carbon Systems) use an amine-functionalised adsorbent in TVSA process and are responsible for just over 41 000 t-CO_2_ year^−1^ and 4000 t-CO_2_ year^−1^ of installed capacity, respectively. The maximum technology readiness level (TRL) can therefore be categorised as 8–9. Meanwhile, Airthena, Hydrocell, Skytree, Avnos, DACMA, Exxonmobil, the University of Twente, and Carbon Collect have also deployed small research pilots employing adsorption processes. Table 13 summarises the plants deployed to date. Climeworks has shared some of their experiences operating Orca.^89^ The design removal capacity of the plant is 3000 tonnes per year, of which 2500 tonnes per year can be achieved with the current filter material. The maximum weekly run rate of the plant, in reality, has been 1900 tonnes per year using a two-year degraded filter material, and the actual amount of CO_2_ removed in 2023 was 1000 tonnes. Additionally, in 2022, only 487 tonnes of CO_2_ was removed whilst mechanical challenges were being solved. All of this demonstrates the challenges faced when operating a first-of-a-kind plant, especially in the first few years. However, it also demonstrates that these challenges can be overcome and that the solutions can be implemented in the next plant. Unfortunately, beyond this, very little data is publicly available on these plants' performance, successes and failures. This would be an invaluable addition to the literature.

#### 3.1.6. Energy consumption

One of the highlighted challenges for solid adsorbent processes is the energy consumption. In a TVSA process, the energy is consumed in three fundamental processes: (i) blowing air through the sorbent, (ii) evacuating the chamber containing the sorbent, and (iii) providing heat to the column.^46,74,83^ Of the papers analysed in this review, the majority of estimates of thermal energy fall in the 5–25 GJ t-CO_2_^−1^ range with a median of 9 GJ t-CO_2_^−1^ and a mean of 13 GJ t-CO_2_^−1^ (note that Section 3.10 includes a conversion to work equivalent for comparison with electricity driven DAC technologies).^38,46,61,62,67–69,79,82,85,87,88,90–99^ In a process without a steam purge, most of the heat is needed for heating the adsorbent, including any adsorbed species, and desorbing co-adsorbed water.^46,74,83^ CO_2_ desorption will consume 1.6–2.7 GJ t-CO_2_^−1^ for heats of adsorption between 70–120 kJ mol^−1^, which covers most amine-functionalised adsorbents, for example. The main difference in using a steam purge is that less heat is required to desorb H_2_O, and H_2_O may net adsorb during the purge.^74^ However, more heat is potentially lost from steam leaving the column, while this heat may be largely recoverable. Notably, the heat required is low temperature. All articles analysed used regeneration temperatures from 80–120 °C, with a median of 100 °C and mean of 90 °C. This means there is a high potential to use low-cost heat, such as waste heat or efficient heat pumps.

For the electrical work requirement, most estimates fall between 0.5–5 GJ t-CO_2_^−1^, with a median of 0.8 GJ t-CO_2_^−1^, and a mean of 3.4 GJ t-CO_2_^−1^.^38,46,61,69,79,83,85,87,90,92–94,96,97,99^ Breakdowns between the work required for blowing air through the adsorbent and evacuation of the adsorption chamber are reasonably uncommon, and vary from the vacuum step dominating to the air blowing dominating.^46,74,83^ As this is clearly not well understood, more work needs to be done to characterise this breakdown.

Of the studies that provide details of energy requirements, the vast majority are TVSA using amine-functionalised adsorbents. The literature on energy requirements using humidity swing adsorption or physisorbents is relatively sparse.

Pathways and avenues are available to reduce the heat and electrical work requirements. For example, humidity swing materials can drastically reduce the temperature lift the adsorbent needs to desorb the CO_2_, whilst passive contacting with air can eliminate the fan electrical requirement altogether.^100^ Of course, there are almost always trade-offs to be considered, *e.g.*, passive contacting will likely reduce productivity, thus increasing capital cost intensity per unit of carbon captured. Equally important are efficient heat integration, process optimisation, and the design of low pressure drop contactors to ensure that the system wastes little energy.

#### 3.1.7. Productivity

Productivity is an important proxy metric for the levelised capital costs given a particular process cycle and material. Although, the trend between levelised capital cost and productivity will certainly not be linear and this proxy will not hold well when comparing across different types of beds, for example, packed *vs.* structured contactors. In a case where the capital for the adsorbent-containing chamber, downstream CO_2_ processing, and balance of plant are optimised and the adsorbent cost is roughly the same across materials, the maximum productivity out of a set of process and material combinations will lead to the lowest levelised capital cost. This is arguably a more important metric than energy consumption due to the current dominance of capital cost towards total cost for solid adsorbent direct air capture.^58^ Assessing the productivity correctly for TVSA processes requires accurate heat and mass transfer models. Table 2 below shows some of the estimates of productivity available.

**Table 2:** Productivity estimates for DAC temperature vacuum swing adsorption technology available in literature

Conditions	Productivity [t-CO_2_^−1^ m^−3^_bed_ year^−1^]	Ref.
Steam purge TVSA process with amine-functionalised cellulose (20 °C and 50% relative humidity)	0.9–4.4	Stampi-Bombelli *et al.* 2020^85^
TVSA process using a range of amine-functionalised materials with a range of assumed mass and heat transfer coefficients (20 °C and 43% relative humidity)	4.4–96.4	Sabatino *et al.* 2021^83^
Nitrogen purge TVSA process using a benchmark amine-functionalised polymer resin (20 °C and 50% relative humidity)	12.1–48.4	Schellevis *et al.* 2021^82^
TVSA process using a hypothetical amine-functionalised material (variation across all ambient conditions)	13.0–77.9	Wiegner *et al.* 2022^49^
Steam purge TVSA process using a benchmark amine-functionalised polymer resin (temperature and relative humidities covering locations across the globe)	30.9–38.6	Sendi *et al.* 2022^48^
TVSA process using a benchmark amine-functionalised polymer resin (15 °C and 55% relative humidity)	34.7–36.7	Young *et al.* 2023^46^
TVSA and steam-purge TVSA processes optimised for specific energy and productivity for five adsorbents (three chemisorbents and two physisorbents), and varying mass transfer coefficient parametrically	2.9–104.0	Balasubramaniam *et al.* 2024^101^

From the set of literature studied, it appears that anything from 1–100 t-CO_2_^−1^ m^−3^_bed_ year^−1^ could be a reasonable estimate of the productivity of a TVSA process today. Although the range of materials studied in Table 2 is minimal (only seven real materials were modelled), and all studies consider a packed bed. The recent study by Stampi-Bombelli *et al.*^75^ show that monolith contactors can significantly improve TVSA productivity, suggesting the need for more monolith DAC modelling studies. The literature is also in desperate need of productivity estimates for humidity swing adsorption and adsorption using other materials like physisorbents to make a fair comparison between these different processes.

There are two main levers to improve productivity. The volumetric working capacity¶¶Generally defined as the moles or mass of CO_2_ recovered per cycle per unit volume of adsorbent bed. of the material can be increased, and the overall cycle time can be reduced. It has been shown that the adsorbent capacity of CO_2_ is somewhat important. However, the process is primarily driven by heat and mass transfer in the design space of amine functionalised materials.^74,101^ Heat transfer can be improved through alternate heat transfer mechanisms like direct steam contacting, microwave regeneration, Joule heating, or inductive heating. Mass transfer can be improved by better tuning of pore structure and distribution and structuring of the material into monoliths or laminates. Further, process optimisation is also crucial to extracting the full potential of any given material.

#### 3.1.8. Sorbent lifetime

The final key aspect of adsorption-based DAC is the degradation of the adsorbent material. There are concerns over the lifetime of amine-functionalised materials in particular.^65^ Jahandar Lashaki *et al.*^64^ have covered the fundamental literature on this in great detail, so this section shall not focus on the underlying science behind the degradation of amine-functionalised materials or laboratory experiments characterising this.

Carneiro *et al.* identify the three main conditions where degradation can occur in amine-functionalised adsorbents: (i) adsorption (high oxygen concentration, low temperature, long times), (ii) regeneration (low oxygen concentration and high temperature, variable times), and (iii) re-exposure to air after cooling (high oxygen concentration, intermediate temperature, and short times).^102^ However, to our knowledge, there is no literature that includes deep analysis of the relative contributions of each of these environments to the overall degradation rate of the material in a given process. If process engineers are to design a process that is inherently protective of a material, then this knowledge is a requirement. A powerful tool towards this analysis would be an accurate degradation rate equation to integrate with the dynamic concentration and temperature profiles created by process modelling. Nezam *et al.*^103^ developed an equation, but it is yet to be validated against real process data.

There is little data at all to show the stability of humidity swing adsorbents beyond short laboratory scale tests over 10 cycles.^47^ There needs to be testing of these materials at a greater scale over a longer period in real process conditions. Equally, the stability of any physisorbent considered for DAC should be assessed individually, as each one may have different constraints, such as a temperature limit or the concentrations of water vapour, oxygen, or trace poisons in the system.

However, it should be emphasised that there is no data in literature on how any DAC materials degrade over an extended period in a pilot or commercial scale process where any given material may experience various oxidative environments and poisons. This will be crucial data when deciding whether adsorbent degradation will contribute significantly to adsorption-based DAC costs.

### 3.2. Liquid sorbents with calcium looping regeneration

Liquid sorbent DAC with Ca-looping involves using a strong alkali solution to capture CO_2_ and using a calcium loop for CO_2_ desorption and absorbent regeneration. The process originated when Lackner *et al.*^25^ first discussed using a solution of calcium hydroxide Ca(OH)_2_ as a chemical absorbent for CO_2_ separation from ambient air. Liquid sorbent regeneration can be done using a CaCO_3_–CaO–Ca(OH)_2_ loop, where calcium carbonate calcination occurs at very high temperatures (∼900 °C), the most energy intensive step in the process. The heat source is usually an oxy-fired kiln burning natural gas, which adds to the system's CO_2_ emissions and brings down the net CO_2_ removed by the process: while in carbon engineering designs the emissions from the natural gas combustion are co-captured, there are still considerable greenhouse gas emissions upstream the natural gas supply chain (*e.g.*, ref. 104). Other low carbon thermal energy sources like solar thermal, renewable electric heating, green hydrogen, biofuels, etcetera need be explored to reduce or replace the methane usage in the future.

Liquid sorbents offer the advantage of continuous process operation, compared to cyclic solid sorbent processes using a pressure, temperature, or moisture swing. This enables the liquid sorbent systems to benefit from economies of scale, also resulting in favourable costs (Section 4.1). The main disadvantages include the complexity of the sorbent regeneration facility, the periodic need for water replenishment in dry environments, and the fact that current designs still rely on natural gas to generate the high regeneration temperatures needed. Carbon Engineering, a DAC company based in Canada, has been the sole liquid sorbent DAC company with an active pilot plant, which has been the basis for a majority of the research literature and analysis on liquid sorbent DAC systems.^105^

#### 3.2.1. Major challenges

Three major challenges of using liquid sorbents for direct air capture are the low so-called cyclic CO_2_ loading of the sorbent (∼0.05 mol CO_2_ per mol K^+^), the need for water replenishment, and the complex sorbent regeneration cycle, including high calcining temperatures. The first is inherent to the low ‘driving force’ of CO_2_ in the air. When the caustic sorbent enters the air contactor, the CO_2_ over K^+^ loading is approximately 0.25 mol mol^−1^. Contacting the sorbent with the air may increase this to approximately 0.30, which is only a small increase, meaning that large equipment is needed to only capture a small amount of CO_2_. This also increases the complexity of sorbent regeneration, necessitating the multi-step regeneration loop including calcium carbonate precipitation. The need for considerable water replenishment is especially apparent for hot, dry climates, where the water evaporation from the air contactor exceeds 10 tonne H_2_O per tonne CO_2_ captured, as nicely illustrated by An, McCoy, and collaborators.^28,106^

#### 3.2.2. Materials

A strong alkaline solution of potassium hydroxide (KOH) with an approximate composition of 1.0 M OH^−^, 0.5 M CO_3_^2−^, and 2.0 M K^+^, acts as the liquid absorbent for CO_2_.^105^ A calcium hydroxide (Ca(OH)_2_) solution is then used in the calcium loop to precipitate out calcium carbonate and regenerate the KOH solution. These strong alkali solutions are stable and offer the advantage of fast CO_2_ absorption kinetics. This comes at the cost of the mentioned very high regeneration energy requirement to desorb the captured CO_2_ at temperatures as high as 900 °C. Earlier studies involved the use of NaOH as the liquid sorbent, as summarized in Table 3. Mahmoudkhani *et al.*^107^ introduced a lower energy alternative of using sodium tri-titanate (Na_2_O·3TiO_2_) instead of Ca(OH)_2_ to regenerate NaOH absorbent.

**Table 3:** Summary of liquid sorbent with calcium looping DAC process specifications published in the scientific literature. All studies are modelling studies

DAC sorbent	Sorbent regeneration reagent	Air contacting mechanism	Regeneration temperature (°C)	Energy requirement (GJ per tonneCO_2_)			CO_2_ produced pressure (bar)	CO_2_ purity (%wt)	Scale (kt per year)	Ref.
				Thermal	Electric	Total				
KOH	Ca(OH)_2_	Cross-flow	900	5.25	1.32	6.57	150	97.12%	980	105
KOH	Ca(OH)_2_	Cross-flow	900	9.18–12.18	0.74–1.66	9.92–13.84	1	≥98%	1000	110
KOH	Ca(OH)_2_	Cross-flow	900	5.11–8.10	1.32–2.75	6.43–10.85	1	>97%	0.365	121
KOH	Ca(OH)_2_	—	900	—	—	8.79	151	—	980	113
KOH	Ca(OH)_2_	Cross-flow	900	—	—	8.30–11.10	151	—	1000	28 and 122
XOH (X = K, Na)	Ca(OH)_2_	Counter-flow, cross-flow	900	5.25–8.10	1.32–1.80	6.57–9.90	1–150	—	1000	21
NaOH	Ca(OH)_2_	—	900	7.25	2.80	10.05	80	—	—	119
NaOH	Na_2_O·3TiO_2_	Cross-flow	800–860	—	—	3.07 (for sorbent regeneration loop only)	100	—	—	107
XOH (X = K, Na)	Ca(OH)_2_	Counter-flow, cross-flow	900	80% of total	20% of total	7.30–8.90	150	≥98%	1000	112
NaOH	Ca(OH)_2_	Counter-flow	900	8.10	1.78	9.88	100	—	1000	123
NaOH	Ca(OH)_2_	Counter-flow	900	8.10	1.78	9.88	100	—	1000	124
NaOH	Ca(OH)_2_	Counter-flow	900	6.70	1.73	8.43	100	—	1000	125
NaOH	Ca(OH)_2_	Cross-flow	—	17.00–54.00	3.00–3.50	20.00–57.50	101.325	—	76	118
NaOH	Ca(OH)_2_	—	900	6.04–8.80	1.58–1.79	7.62–10.59	58	—	420	120

#### 3.2.3. Processes

**Fig. 4:**
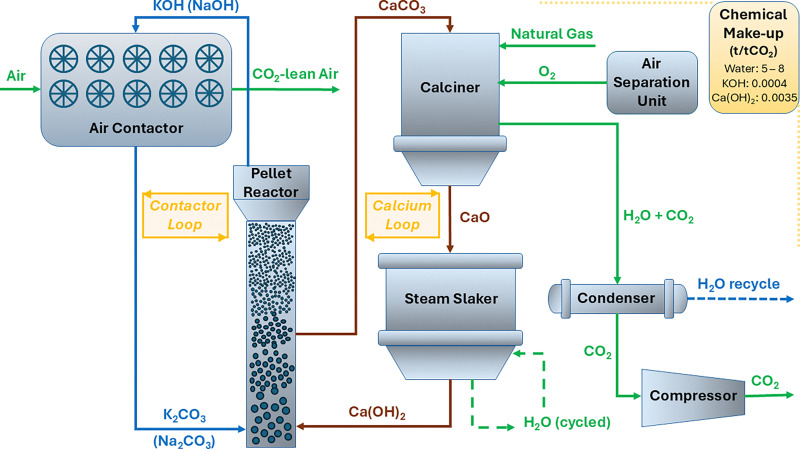
Schematic process diagram for the liquid sorbent DAC process with calcium looping. Green, blue, and brown lines represent gaseous, liquid, and solid flows respectively. Chemicals make-up in tonne/tonne of gross CO_2_ captured. Water make-up requirements vary depending on relative humidity and operating conditions.^28,105,109,110,113^

There are two main parts of the liquid sorbent with calcium looping-based direct air capture process: the air contacting system, and the regeneration facility (Fig. 4). The air-contacting system is responsible for the interaction of ambient air with the liquid absorbent, which can be done either in crossflow or in counter-flow configuration. The carbon dioxide (CO_2_) present in the air feed reacts with the aqueous potassium hydroxide solution to form water and potassium carbonate (K_2_CO_3_). Since air has a very low CO_2_ concentration (∼400 ppm), a large amount of air needs to be blown into the air-contactor per unit time to meet the CO_2_ capture capacity goals. Keith *et al.* determined the contactor area required for 1 MtCO_2_ per year capacity considering 75% CO_2_ capture from air and 1.5 m s^−1^ air velocity to be 38 000 m^2^. This is two orders of magnitude larger than the largest commercial packed tower contactor,^108^ suggesting there may be an opportunity for air contacting intensification, *e.g.*, *via* improved contactor design. Another critical element of contactor design is maintaining low pressure drop, again given the very large amount of air treated air needed. Nowadays, mostly cooling tower type packing air contacting design are used, while there clearly is an upside for further intensification.^108^ Stolaroff *et al.*,^109^ analysed the feasibility of spray-based air contactors that spray NaOH sorbent as fine mist-like droplets, where the levelized cost of capture was smaller for small (50 μm mean diameter) droplets, achievable by off-the-shelf spray nozzles.

The regeneration facility serves two purposes: to recover the liquid absorbent (potassium hydroxide), and to release the captured CO_2_ as a concentrated stream for downstream utilization or storage. These two purposes are mediated *via* a calcium loop (Ca-loop, Fig. 4). The potassium carbonate from the air contactor is reacted with calcium hydroxide (Ca(OH)_2_) in a causticizer, also known as the pellet reactor, to form calcium carbonate (CaCO_3_) precipitates and regenerate the aqueous potassium hydroxide to be reused in the air-contactor. The excess liquid in the CaCO_3_ slurry is removed by passing the slurry through a clarification and filter press step. The CaCO_3_ precipitate is then fed into a calciner operated at 900 °C to produce solid calcium oxide (CaO) and an almost 99% pure stream of CO_2_ which can be further compressed to 100–150 bars for transportation and storage. The solid CaO is reacted with water in a steam slaker at 300 °C to regenerate the calcium hydroxide to be reused in the causticizer. An air separation unit provides oxygen with 99.8% purity to be fed in the oxy-fired kilns to ensure that the kiln exhaust comprises only of CO_2_ and water, which are easier to separate.^110^ Due to the continuous cyclic nature of the process, the feedstock is fed once in the beginning and reagent loss during the process is replenished *via* make-up streams. For the air-contactor, some amount of absorbent is lost *via* aerosol formation and spray drift.^108^ For the pellet reactor, the calcium retention rate is maintained above ∼85%, where some loss is observed as fines.^105^ Materials make-up is essential as it is hard to prevent reagent loss and contamination (*e.g.*, by insects, birds, dust, particulates, NO_*x*_, and SO_*x*_) due to the nature of DAC. The calcium makeup is done using CaCO_3_ for its lower carbon footprint and cost than other lime alternatives (CaO and Ca(OH)_2_).^110^

Liu *et al.* combined the liquid sorbent DAC system with the electrolysis driven conversion of CO_2_ to synthetic fuel *via* Fischer–Tropsch synthesis (FTS) to show that this pathway can produce fuel with a lower carbon intensity than conventional fuels, however, it does not deliver net carbon dioxide removal. The study also reported that using an electric calciner can reduce the life cycle emissions of this FTS-DAC process by 60% as compared to oxy-fired natural gas calciner.^111^ Keith *et al.*^105^ discussed alternative configurations with the same four main unit operations (two loops). For minimizing gas input, the process omits the gas turbine and runs on grid electricity to meet the power requirements not met by the steam cycle from the steam slaker, making it an ideal configuration for locations with low-carbon low-cost electricity. CO_2_-to-fuel synthesis options using hydrogen feedstock produced by electrolysis involves less intense (30 bar) CO_2_ compression and the air separation unit is not required when sufficient oxygen can be produced by the electrolyser. McQueen *et al.*,^112^ compared the baseline process with the case of using an electric calciner where process power is obtained from different energy systems: natural gas, solar, wind, nuclear, and geothermal, showing the potential advantage of an electric calciner to exploit low-carbon energy alternatives, again with the priority of utilizing them for low-carbon grid electricity over DAC.

As this process involves corrosive alkali solutions, special alkali-resistant materials need to be employed for lining or designing the process equipment. Often, plastics are used. The use of corrosive alkalis also calls for an efficient chemical waste disposal facility to ensure no damage is done to the environment and communities in the locality.

#### 3.2.4. State of development and deployed plants

Carbon Engineering, a liquid sorbent-based DAC company from Canada, is the only DAC company working with the strong alkali liquid sorbent (potassium hydroxide) coupled with a calcium looping process. It has deployed a pilot plant on a 0.5-hectare industrial site in Squamish, British Columbia (BC), Canada, operating since 2015 with its expansion in 2017.^20,105^ The pilot plant has the capacity to capture up to 1 tCO_2_ per day (equivalent to 0.365 kt CO_2_ per a).

Using the US Department of Energy's definition of the technology readiness level (TRL) scale, Carbon Engineering's DAC technology stands at TRL 6 or 7. Liquid-sorbent based DAC systems benefit from economies of scale, desiring plant capacities larger than 0.5 MtCO_2_ per year. The scale-up from pilot plant to such large industrial scale DAC is a size increment of two orders of magnitude, which is technologically risky, time-demanding and requires huge financial investments. In collaboration with 1PointFive, a subsidiary of Occidental Petroleum Corporation's Low Carbon Ventures business, Carbon Engineering announced a DAC plant deployment plan in June 2022 to execute numerous DAC projects across the globe. 1PointFive proclaimed a scenario of constructing 70 large-scale DAC facilities, each with an expected capacity of 1 MtCO_2_ per year, by the year 2035.^114^ The site preparation of the first such DAC facility, STRATOS, began in 2022, and is expected to be commercially operational in mid-2025 in Ector County, Texas, USA, capturing 0.5 MtCO_2_ per year.^114,115^ As a part of the South Texas DAC Hub, King Ranch in Kleberg County, Texas, USA, is planned as the site for the next DAC facility with the front-end planning and engineering being started since October 2022.^116^ Based on a Joint Study Agreement signed between 1PointFive and Abu Dhabi National Oil Company (ADNOC) in October 2023, the potential to install a 1 MtCO_2_ per year DAC facility in Abu Dhabi is being explored.^117^

Carbon Engineering's pilot plant, while it includes key subsystems (air contactor, pellet reactor, slaker, and a batch-operated oxy-fired natural gas calciner), is not a complete miniature of a commercial plant as it lacks low technical risk components (like gas clean-up, CO_2_ compression, *etc.*)^105^ The coupled air-contactor and pellet reactor captures ∼0.6 tCO_2_ per day from the atmosphere, with CO_2_ capture rate and capture fraction being dependent on the air inlet velocity amongst other parameters. For example, an air inlet velocity of 1.4 m s^−1^ results in a maximum capture rate of 45 kg CO_2_ per h with 42% capture fraction. These values are lower than the 1 tCO_2_ per day net removed at 75% capture fraction for the overall pilot plant's design.^105^ The air contactor demonstrated stable pressure drop and matched the specified performance over 0.75 years of intermittent operation. The calciner achieved >98% CaCO_3_-to-CaO conversion at 90 kg CaCO_3_ per h with stable fluidization and minimal fouling.^105^ The pilot plant's measurements helped building and validating unit-level performance models informing the Aspen Plus simulation that reported the process’ energy and material balance data (*e.g.* 74.5% CO_2_ capture fraction in the air contactor; 90% calcium retention in the pellet reactor).^105^ Real-world performance remains unclear due to absence of empirical data on long-duration integrated continuous cyclic operation, and unit-level process efficiencies and material and energy losses.

#### 3.2.5. Energy consumption

The reported total energy requirements for a 1 MtCO_2_ per year capacity liquid sorbent-based DAC plant vary between roughly 6 and 14 GJ per tCO_2_ (Table 3), excluding the outlier by Keith in 2006.^118^ The National Academies of Science, Engineering, and Medicine estimated the total energy requirement at 9.92–13.84 GJ per tCO_2_ captured, where 0.74–1.66 GJ per tCO_2_ is required as electricity (to run the fans, pumps, slaker, causticizer, and air separation unit) and 9.18–12.18 GJ per tCO_2_ is required in the form of thermal energy (for the heater/dryer and calciner).^110^ Process design modifications and heat integration can help further reduce this high energy demand. Keith *et al.*^105^ arrived at a lower thermal energy demand estimate of 5.25 GJ per tCO_2_ using a rigorous heat integration design incorporating two heat recovery cyclones and steam slaking. We note it may be difficult to realise such low energy requirements without sufficient testing and analysis under realistic scenarios. The most energy intensive step is the recovery of the absorbent using oxy-fuelled calciner, consuming ∼63% of the total energy requirement.^25,110^ The minimum theoretical work to capture 75% of the CO_2_ in air stream generating 98% pure CO_2_ is 0.45 GJ per tCO_2_, which indicates the exergy efficiency of the liquid sorbent-based DAC process to be 3.5–5.3% only.^110^

The system's calciner incorporates heat recovery with the help of a heat exchanger and a condenser. The hot exhaust gas from the calciner (at 900 °C) is cooled to 200 °C *via* the heat exchanger while heating up the incoming gas. The exhaust is then further cooled to 30 °C using a condenser. There is also a potential to recover an additional 2.4 GJ per tCO_2_ of heat released from the hydration of CaO for use in CaCO_3_ drying.^110,119^ The drying of calcium carbonate pellets before calcination has a significant contribution to the energy demand which can be reduced using an innovative pellet reactor with an efficient CaCO_3_ dewatering system to bring the residual water content of the solid CaCO_3_ pellets entering the calciner to be as low as 10%.^120^

Long-Innes and Struchtrup^113^ studied the thermodynamic losses involved in the Carbon Engineering's proposed 1 MtCO_2_ per year capacity DAC plant and reported that 279.2 MW of work potential is consumed to remove 111.9 tCO_2_ per h, of which 21.20 MW is thermodynamically required minimum reversible work. This implies an irreversible work loss of 258 MW including the evaporation (loss) of water in the air contactor system (Table 4). Finally, An *et al.*^28^ analyzed the impact of climate on the liquid solvent-based DAC systems and observed that hot and humid conditions are necessary for high CO_2_ capture rates as they enhance absorption kinetics. Annual average weather conditions, along with the carbon sequestration opportunities, make certain geographical locations, like the southern tier states in the USA, more conducive to liquid solvent DAC according to the geospatial performance analysis by Brooks *et al.*^106^ The energy source for liquid solvent DAC can be decided independent of weather constraints.^106^

**Table 4:** Breakdown of energy requirements and irreversible thermodynamic work loss for a 1 MtCO_2_ per a liquid-sorbent DAC plant with calcium looping, based on National Academies of Science, Engineering, and Medicine^110^ and Long-Innes & Struchtrup^113^

Unit operation	Energy required (GJ per tCO_2_)	Irreversible thermodynamic work loss (GJ per tCO_2_)
Air-contactor fans	0.32–1.18	0.296 + 0.193 (evaporation) = 0.489
Solvent pump	0.048–0.065	—
Slaker	0.005	0.354 (steam turbine)
Causticizer (pellet reactor)	0.109	—
Air separation unit	0.30	0.335
Heater/dryer	3.18	—
Oxy-fired calciner	6.0–9.0	2.027
Exhaust gas cooling	−1.5 (heat recovery)	0.251 (water knockout) + 0.315 (compression system) = 0.566
Chemical exergy dissipation	—	3.195
Power generation for the process	—	1.335 (combined cycle gas turbine)
Total (w/o exhaust heat recovery)	9.9–14	—
Total (w/exhaust heat recovery)	8.4–12.5	—
Total work loss	—	8.301

Table 4 gives a summary of the energy requirements and the irreversible thermodynamic losses for each unit operation in a 1 MtCO_2_ per a capacity liquid-sorbent DAC plant.^110,113^

### 3.3. Liquid sorbents with electrochemical regeneration

The conventional regeneration methods for the DAC liquid sorbents involve a temperature swing process, discussed in Sections 3.2 and 3.5. Alternatively, electrochemical regeneration of the liquid sorbents has drawn much attention in recent years. The advantages of the electrochemical DAC process include easy installation and high scalability due to modular cell design, operating under ambient temperature and pressure, plus potentially low energy consumption. Furthermore, as the electrochemical regeneration step is powered by electricity only, the whole process can be integrated with renewable electricity to reach net negative carbon emissions.

Based on the sorbent properties, electrochemical DAC can be categorized into direct regeneration and pH-swing processes. Direct regeneration means that the capture absorbent can directly participate in the electrochemical redox reactions as a means of regeneration. Electrochemical regeneration of nucleophiles (including benzylthiolate,^126^ bipyridine,^127^ quinones^128^) has been studied for carbon capture from flue gas. A disadvantage is that the reduced state of these organic compounds reacts rapidly with O_2_.^129,130^ Moreover, Wang *et al.*,^131^ investigated the electrochemically mediated amine regeneration (EMAR) process for carbon capture from flue gas, where ethylenediamine (EDA) was regenerated electrochemically by competitive binding of EDA with Cu^2+^ ions *via* electrical polarization of copper electrodes. The effect of oxygen on the stability of the copper electrodes remains unknown for this technology. Therefore, the application for DAC is yet to be understood, and these two processes are, hence, excluded from this review.

**Fig. 5:**
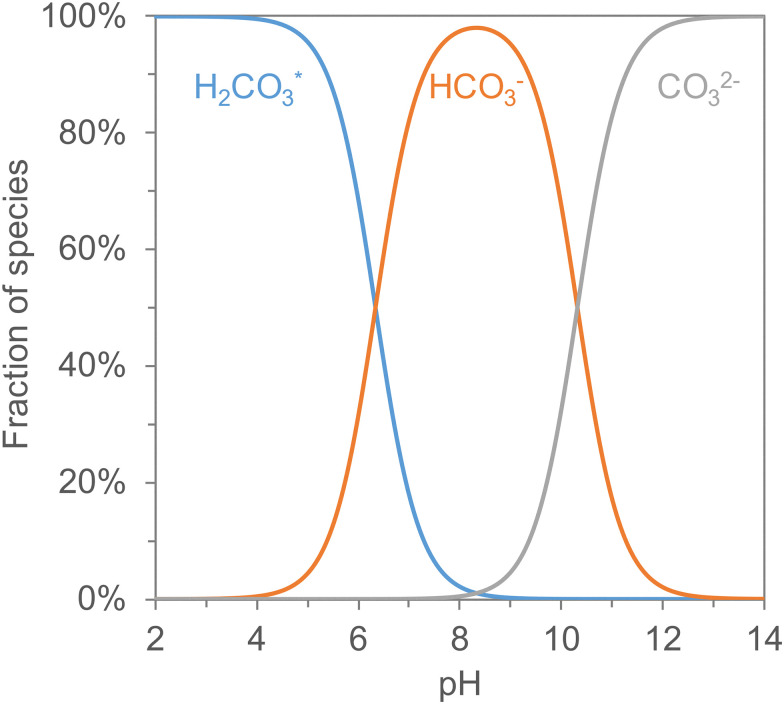
Distribution of carbon species in a 0.5 M Na^+^ solution with varying pH. Note that H_2_CO_3_ (carbonic acid) will usually react instantly to form aqueous CO_2_.

The pH-swing processes rely on the ionic forms of CO_2_ in aqueous solutions Fig. 5. CO_2_ from the air is captured by an alkaline solution through its reaction with OH^−^ (the reaction with H_2_O plays a very limited role down to pH < 10)s. In electrochemically driven pH-swing processes, the pH of the rich absorbent is lowered, desorbing pure CO_2_ gas due to the shift of CO_2_ equilibrium. After separating the desorbed CO_2_ gas from the liquid phase, the pH of the solution is raised *via* the production of OH^−^ to regenerate the alkalinity of the capture absorbent. This pH swing in the electrochemical cell is achieved by redox reactions or water dissociation, *e.g.*, using bipolar membranes (BPMs).

#### 3.3.1. Processes

##### 3.3.1.1. Redox mediated

In redox-mediated processes, the pH swing of the capture absorbent is driven by the redox reactions of the redox-active compounds. The oxidation reaction produces H^+^ that reduces the solution pH while the reduction reaction consumes the H^+^ or produces OH^−^ that increases the pH. Depending on the redox compounds used in the processes, they are categorised into H_2_-based and amine-based processes.

##### 3.3.1.2. H_2_-based

The concept of electrochemical carbon capture from ambient air was first proposed in 1995 by Stucki *et al.*^132^ They studied a DAC process using a KOH solution as absorbent, and CO_2_ was absorbed in the solution by reacting with OH^−^ and forming CO_3_^2−^/HCO_3_^−^. The loaded absorbent was then regenerated by an electrochemical cell, where the oxygen evolution reaction (OER) and hydrogen evolution reaction (HER) occurred respectively at the anode and cathode of the cell. The OER produced H^+^ that acidified the loaded absorbent to desorb CO_2_, while the HER produced OH^−^ that regenerated the alkalinity of the absorbent. Recent studies on this technology have been focusing on the characterization of the process.^133^ However, a notable disadvantage of this process is that the OER also produces O_2_ gas, resulting in the desorbed CO_2_ being mixed with O_2_.

**Fig. 6:**
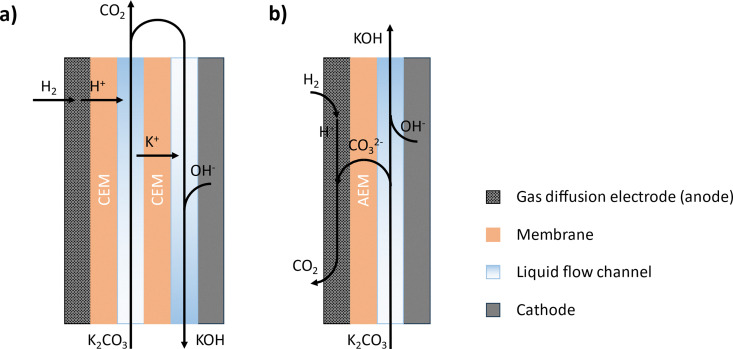
Schematic of H_2_-based electrochemical regeneration of alkaline absorbent (KOH) featuring (a) a cation exchange membrane (CEM) or (b) an anion exchange membrane (AEM).

Alternatively, the hydrogen oxidation reaction (HOR) has been proposed to replace the OER at the anode to avoid the production of O_2_. The H_2_ gas is supplied *via* a gas diffusion electrode (anode), where it is oxidized to H^+^, reducing the pH of the loaded absorbent. Depending on the transported species in the cell, the anode and cathode are separated by either cation exchange membranes (CEM) or an anion exchange membrane (AEM) Fig. 6. The CEM design enables the production of pure CO_2_ gas, and the rapid kinetics of H^+^ and K^+^ transport through the membrane significantly enhance the energy efficiency of the process.^134,135^ On the other hand, the AEM design has the advantage of a more compact cell design and lower electric resistance, while the produced gas stream is a mixture of H_2_ and CO_2_.^136,137^ Moreover, as a variant of the CEM design, Xu *et al.* and Liu *et al.* have developed a two-electrolyser configuration to separate the CO_2_ desorption and alkaline absorbent regeneration in two cells.^138,139^ In this configuration, the redox-active viologens connect the two electrolysers and improve the reaction kinetics. Furthermore, Lin *et al.* developed another variant of the CEM design by replacing one CEM with an AEM.^140^ They have tested both inorganic alkaline sorbent and organic amine-based sorbent and showed the potential of reduced energy consumption compared to the conventional CEM design.

##### 3.3.1.3. Amine-based

**Fig. 7:**
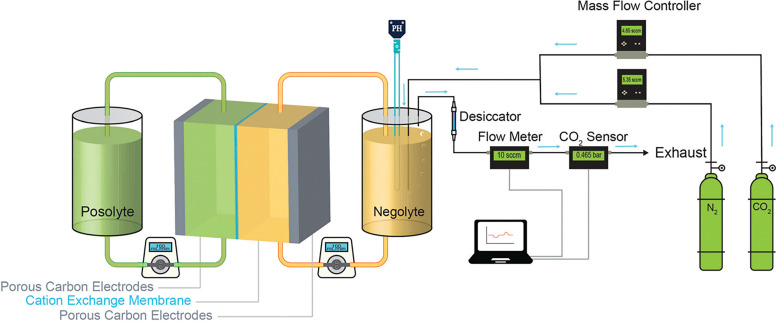
Schematic of Fe(CN)_6_ (posolyte)|DSPZ (negolyte) flow cell for CO_2_ capture/release experiments. Reproduced from ref. 141 with permission from the Royal Society of Chemistry, copyright 2020.

Some amine-based compounds undergo a so-called proton-coupled electron transfer (PCET) during the redox reactions in an electrochemical cell, such that the proton consumption or production during the reactions induces the pH-swing of the liquid solution. Jin *et al.* studied sodium (3,3′-(phenazine-2,3-diylbis(oxy))bis(propane-1-sulfonate)) (DSPZ) dissolved in KCl as the CO_2_ capture solution.^141,142^ The reduction reaction of DSPZ produces OH^−^ that provides the CO_2_ capture capacity of the solution, while the oxidation of the reduced form DSPZH_2_ decreases the pH of the solution that facilitates the desorption of CO_2_. As shown in Fig. 7, the process utilizes ferricyanide/ferrocyanide at another electrode to counter the redox reactions of DSPZ. Moreover, Pang *et al.* proposed to apply sodium (2,2′-(phenazine-1,8-diyl)bis(ethane-1-sulfonate)) (1,8-ESP) in the same cell design.^143^ This phenazine derivative1,8-ESP has approximately twice the solubility in an KCl solution compared to DSPZ, which improves the CO_2_ capture capacity of the solution and energy efficiency of the electrochemical cell.

Seo and Hatton explored the application of neutral red (NR) and leuco-neutral red (NRH_2_) as a PCET redox couple.^144^ Instead of using a different redox compound in the counter compartment of the electrochemical cell, they investigated the oxidation of NRH_2_ and the reduction of NR within the anode and cathode compartments of a single cell. This design enables the continuous operation of the system and simplifies the process design.

##### 3.3.1.4. Bipolar membrane based

**Fig. 8:**
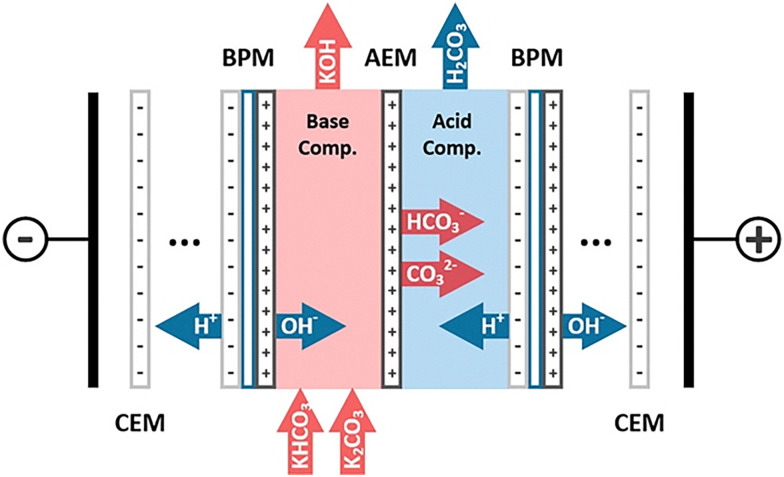
Schematic of a BPMED cell (AEM is anion exchange membrane; CEM is cation exchange membrane; BPM is bipolar membrane). Reproduced from ref. 149 with permission from the American Chemical Society, copyright 2020.

Bipolar membrane electrodialysis (BPMED) is another pH-swing process that can be applied for the regeneration of alkaline DAC sorbents.^145^ Under the applied electrical field, BPMs can dissociate water into H^+^ and OH^−^. While the H^+^ acidifies the CO_2_ loaded absorbent in one compartment, OH^−^ regenerates the alkalinity of the absorbent in an adjacent compartment (Fig. 8). The BPMED cells can be configured with either CEMs or AEMs between the BPMs, determining the species transported though the membranes.^146^ The BPM-AEM configuration suffers from significant undesired transport of OH^−^ through the AEMs, while the BPM-CEM configuration is limited by comparatively higher cell resistances.^147^ Nevertheless, with the development of membrane technologies and improved cell design, the BPMED technology has the potential to reach a low energy consumption of less than 100 kJ mol^−1^ (*i.e.*, <2.3 GJ_e_ per tCO_2_) at industrially relevant current densities over 100 mA cm^−2^.^148^

#### 3.3.2. Major challenges

Despite the promising advancements in electrochemical DAC using liquid sorbents, several challenges limit the widespread deployment of the technology. First, the electrical resistance and overpotential in the cell can lead to increased energy consumption at high current densities, while high current densities are preferred in industrial conditions as they maximise the productivity of a stack. Although strategies have been proposed to mitigate the resistance and overpotential (*e.g.*, a zero-gap configuration to reduce resistance^136^ and optimized cathode geometry to reduce overpotential^137^), further investigations are needed to successfully scale such improvements. Second, water losses during the absorption process through evaporation presents another challenge. For instance, in the amine-based neutral red system, additional water of 1.5 mL h^−1^ was added to compensate for the water loss.^144^ Effective water management strategies are crucial to ensure continuous operation and consistent performance of the electrochemical processes. Third, the reduced form of the amine-based redox compounds can be partially oxidized by the oxygen present in air.^141,142^ This undesirable oxidation reaction reduces the CO_2_ capture capacity of the absorbent and the coulombic efficiency of the electrochemical cell.^143^ Approaches have been proposed to either develop oxygen-insensitive PCET redox compounds^144^ or isolate the redox compound from the alkaline absorbent to prevent the contact with oxygen.^139^ Moreover, the low solubility of amine-based redox compounds constrains the CO_2_ capture capacity of the absorbent and increases the overall operating costs of the process. One approach to addressing this issue is the synthesis of novel high-solubility redox compounds.^143^ Alternatively, the addition of hydrotropic agents can enhance the solubility of these compounds, thereby improving the efficiency and cost-effectiveness of the process.^144^ Finally, some of the electrode and membrane materials required to upscale these technologies are difficult to source at industrial scales. Catalysts for the hydrogen and oxygen evolution and oxydation reactions are typically platinum-group metals, which are expensive materials and scarce in earth's crust. Moreover, ion exchange, and particularly bipolar membranes are novel materials still lacking robust and established supply chains.

##### 3.3.2.1. State of development and deployed plants

Most of the technologies discussed in this section are only tested in a laboratory environment, which gives them a maximum TRL of 4. Although several DAC startups that use electrochemistry have been founded recently,^23^ only few have brought the technology to a higher TRL. In November 2023, RepAir demonstrated their field prototype in an operational environment, which marks a TRL 6 for this company's technology^150^). In December 2023, Mission Zero Technologies launched its first electrochemical DAC plant in collaboration with the University of Sheffield with a CO_2_ capture capacity of 50 tonnes per year, which brought their technology to TRL 6.^151^ They have two more plants to be commissioned in 2025, while CO_2_Cirulair have also commissioned their wet electrochemical pilot plant in 2024 (TRL6).^152^

#### 3.3.3. Energy consumption

As most of the electrochemical DAC technologies are still at the early stage of development, it is difficult to give a direct comparison of the energy consumption of each technology due to the inconsistent testing conditions. To provide a reference, we have listed the reported energy consumption values of some electrochemical DAC technologies with liquid sorbents in Table 5.

**Table 5:** Energy consumption and testing conditions of electrochemical DAC technologies with liquid sorbents. If not mentioned otherwise, the desorbed CO_2_ has a purity greater than 95%. Note that industrial electrochemical processes often operate at current densities of >1000 A m^−2^

Absorbent	pH-swing mechanism	Electricity consumption kJ mol^−1^ (GJ per tonne)	Current density (A m^−2^)	Ref.
KOH + K_2_SO_4_	H_2_-based redox reaction	247 (5.6)^a^	150	135
KOH	H_2_-based redox reaction	290 (6.6)^b^	1000	137
LiOH	H_2_-based redox reaction	167 (3.8)^c^	100	139
MDEA	H_2_-based redox reaction	63 (1.4)^d^	20	140
KCl + DSPZ	Amine-based redox reaction	121 (2.8)^e^	200	142
KCl + NR	Amine-based redox reaction	65 (1.5)^f^	Diminutive	144
KOH	Bipolar membrane	100 (2.3)^g^	50	145
		200 (4.5)^h^		

a Pure CO_2_ desorbed into a partial vacuum (0.3 atm) gas phase.

b Experimentally obtained by feeding pure K_2_CO_3_ solution as mimic loaded absorbent into the electrochemical cell. CO_2_ desorbed in a mixture with H_2_.

c Practical energy consumption with over 200 hours of stable operation.

d CO_2_-saturated MDEA fed into the electrochemical cell and vacuum pump applied for CO_2_ desorption.

e Extrapolated results from experiments with 0.1–0.5 bar CO_2_ in the feed gas.

f Calculated from cyclic voltammetry results.

g Experimentally obtained by feeding pure K_2_CO_3_ solution as mimic loaded absorbent into the electrochemical cell.

h Experimentally obtained by feeding pure KHCO_3_ solution as mimic loaded absorbent into the electrochemical cell.

### 3.4. Solid adsorbents with electrochemical regeneration

#### 3.4.1. Process principle

In an emergent branch of electrically driven DAC, CO_2_ is captured by solid sorbents and regenerated *via* electro-swing by applying a voltage directly to the solid material. The electrode materials can be faradaic or capacitive, which determines if the CO_2_ reacts with the solid or does not, respectively.

**Fig. 9:**
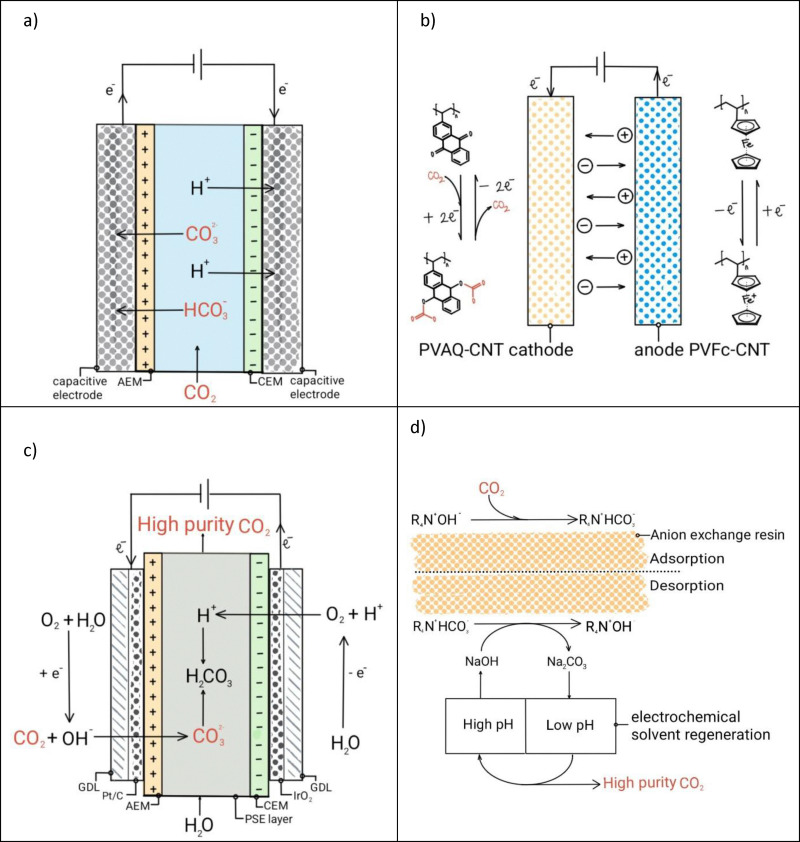
Schematic representation of (a) supercapacitive swing CO_2_ adsorption, (b) faradaic electro-swing reactive adsorption, (c) fuel cell type CO_2_ absorption, and (d) CO_2_ absorption in resins assisted by electrochemical regeneration. Reproduced from ref. 162–165 with permission from the American Chemical Society copyright 2022, 2018; the Royal Society of Chemistry, copyright 2019; and Springer Nature, copyright 2023.

Fig. 9 shows the process principles for the technologies described in this section.

#### 3.4.2. Processes

##### 3.4.2.1. Super-capacitive processes

In super-capacitive swing adsorption, CO_2_ dissolves in a liquid absorbent, to form HCO_3_^−^ or CO_3_^2−^ ions which then migrate towards the electrodes’ pores driven by the applied voltage. Kokoszka and Landskron *et al.*,^153^ demonstrated that the adsorption and desorption of bicarbonate ions from deionized water solutions into capacitive electrodes drives the CO_2_ (g) absorption and desorption from a gas phase. Further developments in this field included enhanced materials^154^ with larger surface area and improved charging–discharging protocols.^155^ Similarly, Legrand *et al.*,^156^ proved super-capacitive CO_2_ capture with additional ion exchange membranes to enhance process efficiency (Fig. 9a). The energy requirement of such system was found to be approximately 40 kJ mol^−1^ CO_2_ at 15% CO_2_ concentrations. Finally, Xu *et al.*,^157^ reported an energy consumption of 18 kJ mol^−1^ CO_2_ and a long lifetime of super-capacitive CO_2_ capture by improving the structural properties of the activated carbon and the charging protocol. The low energy consumption demonstrated by this technology is promising. Although the technology demonstrated CO_2_ concentration from a dilute stream, it does not produce a high purity CO_2_ stream, requiring additional effluent processing.

##### 3.4.2.2. Faradaic processes with electrodes that participate in the reaction

Faradaic electro-swing adsorption (ESA) is the newest method discussed in the literature to capture CO_2_. In this technology, upon the application of a voltage, a functionalized electrode directly reacts with CO_2_, which can be subsequently released by reversing the polarity of the system. Voskian *et al.*,^160^ demonstrated CO_2_ capture *via* the carboxyilation of a poly(vinylanthraquinone) (PVAQ) cathode that undergoes a two-electron reduction reaction and complexes with CO_2_. The anode was a polyvinyl ferrocene composite that undergoes a reversible oxidation reaction (Fig. 9b). After the system is saturated, polarity reversal results in reversing the redox reactions, releasing CO_2_ and regenerating the electrodes. An energy consumption of 40 to 90 kJ mol^−1^ CO_2_, and a performance loss of less than 30% after 7000 cycles of operation was demonstrated.^158^ It is important to note that the materials used by Voskian *et al.*, are expensive relative to competing sorbent materials.^159^ Additionally, the reduced form of quinone is highly sensitive to oxygen and therefore not suitable for air. Further testing in the presence of O_2_ is imperative to determining the long-term stability and performance of the material.^158,160^

##### 3.4.2.3. Faradaic processes with catalytic electrodes

Unreactive electrodes are those that act as catalysts (such as Pt in O_2_ and H_2_ evolution reactions) and do not react directly with CO_2_. Zhu *et al.*,^161^ developed a process based on oxygen reduction reactions (ORR) and oxygen evolution reactions (OER) using the architecture of a fuel cell (Fig. 9c). In this system, the hydroxide ions generated by the ORR capture the CO_2_ directly from a gas stream and convert them into carbonate ions (CO_3_^2−^), which transport through a gas diffusion layer (GDL) to the porous solid electrolyte (PSE) layer at the centre of the electrochemical cell. In the opposite electrode, oxygen evolution reactions produce protons (H^+^) that recombine with the CO_3_^2−^ in the porous solid electrolyte to produce carbonic acid (H_2_CO_3_) and high purity CO_2_.

##### 3.4.2.4. Solid adsorption with electrochemical solvent regenration

In an alternative combination of technologies, Shu *et al.*,^162^ demonstrated a process using anion exchange resins to capture CO_2_. The resins were regenerated by contacting them with an alkaline (NaOH) solvent. The resulting rich solvent was regenerated using a pH-swing approach based on hydrogen reduction and hydrogen evolution reactions, as explained in the previous section on electrochemical solvent regeneration.

#### 3.4.3. Major challenges

##### 3.4.3.1. Novelty of the technology

Electric swing adsorption and regeneration of CO_2_ in solid sorbents is one of the newest carbon capture technologies developed, with first publications on faradaic ESA in 2019 and on super-capacitive ESA in 2014. All reported technologies have only been tested on the lab scale and at low current densities (<15 mA cm^−2^). In most cases, these systems are demonstrated in a batch mode, and they tend to display very low energy consumption, while the proof of concept did not demonstrate high purity CO_2_ production. Voskian *et al.*,^160^ showed their proof of concept using a closed cell of electrodes impregnated with ionic liquid under a CO_2_ atmosphere. The adsorption capacity of the functionalized material was determined by the pressure changes in the closed chamber where the CO_2_ is adsorbed upon charging and desorbed upon discharging. Although this is an important material characterization method, it has no practical application, and the purity of the CO_2_ stream produced cannot be assessed. A more practical flow-by process configuration where the diluted CO_2_ stream flows between electrodes, was demonstrated by Hemmatifar *et al.*^158^ However, the approach used an atmospheric discharge pressure, instead of high purity CO_2_ production. Faradaic and super-capacitive ESA, have only been demonstrated at low TRL levels where the inlet CO_2_ concentration of the feed was higher than in air, therefore in order to determine the potential for scalability and the energy consumption of the process, these technologies need to demonstrate CO_2_ capture from air and the production of a purified CO_2_ stream.

##### 3.4.3.2. Scalability and stability challanges

The CO_2_ adsorption capacity of the materials is another challenge. For the faradaic ESA, Voskian and Hatton^160^ report CO_2_ adsorption capacities of 0.9 and 1.69 mmol CO_2_ g^−1^ sorbent for CO_2_ concentrations of 15% and 100%, respectively, which is lower than others reported in the literature for conventional adsorbents.^166^ The maximum adsorption capacity reported for super-capacitive materials was even lower (∼0.1 CO_2_ mmol g^−1^ sorbent). Moreover, further research at industrially relevant current densities (>20 mA cm^−2^) is required to determine the potential for scalability. The intricate relation between current density, power consumption, production rate, CO_2_ adsorption capacity and capital expenses requires techno-economic feasibility assessments.

#### 3.4.4. Materials

Typical electrode materials used in faradaic and super-capactive electro-swing adsorption must feature high surface areas, therefore carbon nanotubes and activated carbon are extensively used in the literature. For super-capacitive ESA, Xu *et al.* evaluated the impacts of different activated carbon electrode structures and found that electrodes with a combination of micro- and meso-pores and low oxygen functionalization showed the best CO_2_ capture performance. For faradaic ESA, high surface area materials are functionalized with reactive materials. The first proof of concept described in the literature by Voskian and Hatton,^160^ used a polyanthraquinone–carbon nanotube composite negative electrode to capture CO_2_ upon charging *via* the carboxylation of reduced quinones, and releases CO_2_ upon discharge. On the anode side, polyvinylferrocene (PVFc) was used as an electron acceptor or donor. Additionally, metal organic framework materials (MOFs) offering stability, high surface areas and reactivity may also be attractive candidates for ESA,^167,168^ although they have been typically used for temperature or pressure swing adsorption. Recently, Wenger *et al.*,^169^ demonstrated the combination of a zirconium-based metal–organic framework (MOF) and 9,10-phenanthrenequinone (PAQ) to produce a stable cathodic material that can reversibly capture CO_2_ when cycled between redox states, demonstrated through cyclic voltammetry studies. Finally, Winter *et al.*,^170^ recently demonstrated the synthesis of core–shell particles functionalized with redox-responsive 2-aminoanthraquinone (2-AAQ), which displays high affinity towards electrophilic CO_2_. It is important to note that none of the technologies report waste production, although it is highly unlikely that there is zero waste generation during the material synthesis, and a life cycle assessment of the materials used is necessary for all the electrochemical CO_2_ capture technologies.

#### 3.4.5. State of development and energy consumption

Table 6 summarizes the energy consumption, CO_2_ effluent concentration and the state of development reported in the literature. From the studied papers, only three demonstrated continuous CO_2_ production with CO_2_ concentrations above 95%.^158,161,162^ Although the TRL of faradaic ESA is far behind the other technologies described in this review, its simplicity and low energy consumption make it a promising emerging technology. Verdox, co-founded by Voskian and Hatton, is aiming at upscaling faradaic ESA.

**Table 6:** Summary of reported energy consumptions, CO_2_ effluent concentrations, process configurations and electro-swing types

Energy consumption kJ mol^−1^ (GJ per tonne)	CO_2_ effluent concentration	Process configuration	Type of electro-swing
113 (2.56)	0.1% (∼1000 ppm)	Lab scale bipolar stack	Faradaic electro-swing reactive adsorption.^158^
150 (3.4)	>99%		Electro swing adsorption^165^
537 (12.2)	>95%	Lab scale – 2 electrodes	pH swing enhanced with solid adsorbent.^162^
177 (4.02)	25% from 15% inlet concentration	Lab scale – 2 electrodes	Supercapacitive swing adsorption.^171^
628 (14.3)	Not reported	Lab scale – 2 electrodes	Supercapacitive swing adsorption.^155^
40–90 (0.9–2)	Not reported	Lab scale bipolar stack	Faradaic electro-swing reactive adsorption.^160^
70 (1.6)	25% from 15% inlet concentration	Lab scale – 2 electrodes	Supercapacitive swing adsorption.^172^
97 (2.2)	27% from 15% inlet concentration	Lab scale – 2 electrodes	Supercapacitive swing adsorption.^173^
40 (0.9)	Not reported	Lab scale – 2 electrodes	Supercapacitive swing adsorption, batch mode.^156^
18 (0.4)	Not repoted	Lab scale – 2 electrodes	Enhancing electrochemical carbon dioxide capture with supercapacitors.^157^

### 3.5. Amine and amino acid-based liquid absorbents with thermal regeneration

The main technologies investigated and employed for capture of ambient CO_2_ have been based on the use of solid adsorbents and liquid absorbents, the latter typically utilising caustic-based solutions (Sections 3.2 and 3.3). However, this contrasts with the alkanolamine-based absorption technologies typically used for post-combustion CO_2_-capture. Amine-based liquid technologies have been proven to be scalable, energy efficient and robust under the prevailing conditions of a flue gas^174,175^ but have been hardly considered for DAC.^83,176^

#### 3.5.1. Major challenges

There are four main challenges with the amine based liquid capture technology: (i) absorbent loss through evaporation, drift and degradation; (ii) high water requirement due to water evaporative loss; (iii) high (thermal) energy requirement for absorbent regeneration and (iv) high capital cost, mainly associated with large air contacting equipment.^176^ These challenges need to be addressed to make the amine based liquid capture technology technically, economically, and environmentally viable for DAC.

#### 3.5.2. Materials

Barzagli *et al.*,^177,178^ Kiani *et al.*,^176^ and Sabatino *et al.*,^83^ previously proposed alkanolamine-based liquid absorption processes targeting DAC. They showed that benchmark postcombustion capture solvent monoethanolamine (MEA) has comparable CO_2_ absorption affinity as aqueous hydroxides but performed insufficiently for direct air capture due to significant evaporative MEA losses, making MEA processes economically unviable for DAC. Therefore, Kiani *et al.*,^179^ proposed the use of non-volatile amino acid salts as the absorbent, to reduce the capture cost. They examined six different amino acid salts for DAC, in terms of their stability and mass transfer rate.^180,181^ Additionally, they conducted a fundamental study on absorption rate and capacity of CO_2_ into aqueous sodium alaninate for DAC applications.^182^ The use of aqueous amino acid salts for DAC was also investigated in other studies, evaluating absorption rate and regeneration temperature and energy.^183–186^ Subsequently, aqueous solutions of several amino acid salts for the use in a precipitation aided DAC process were studied.^187^ In one study,^188^ salts of sarcosine and glycine were used in a precipitation aided DAC process with concentrated solar energy used to regenerate the absorbents. In another study, 5 different diamines as liquid sorbents for DAC, in both aqueous and non-aqueous solutions, were investigated. Only the absorption behaviour was examined in this study and relationship between the carbonated species and the structure were developed to guide the selection of suitable amines for DAC. The authors concluded that the high CO_2_ capture efficiency of diamine molecules is closely related to the number of unhindered primary amino groups in their chemical structure.^177^

#### 3.5.3. Processes

**Fig. 10:**
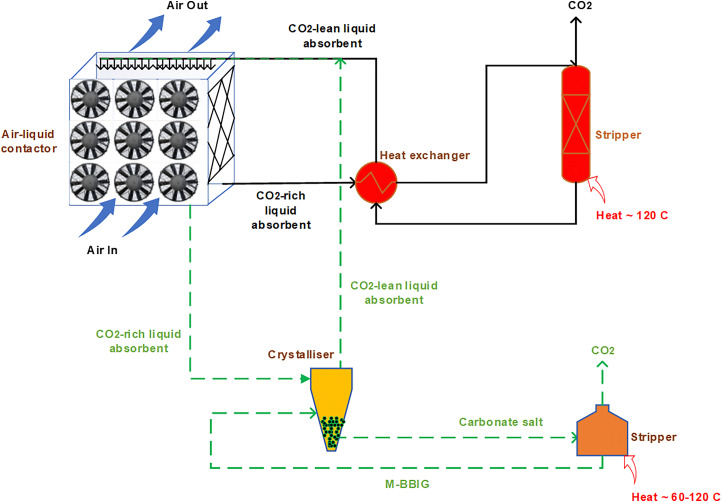
Schematic of two different amine-based DAC processes. Solid line, standard amine-based DAC process configuration, and dashed green line, phase change amine-based DAC process configuration.

Several process configurations for amine based liquid DAC have been proposed. The most obvious one is similar to what is used for point-source CO_2_ capture, with some process modifications that are required mainly due to the low concentration of CO_2_ in ambient air. This process typically consists of two steps; first, the ambient air is fed into an absorber where the CO_2_ reacts with the absorbent agent. The CO_2_-rich liquid absorbent is then heated in a desorber to release the CO_2_ and be regenerated and reused.^83,176^ Other studies proposed a phase change amine-based DAC process in which the regeneration of absorbents occurs at lower temperature, enabling the use of waste heat and solar heat and reduce the extent of thermal absorbent degradation.^183^ In such process, CO_2_-rich liquid absorbent reacts with solid *meta*-benzene-bis (iminoguanidine) (*m*-BBIG), resulting in regeneration of the amino acid and crystallisation of carbonate salts in a crystalliser, followed by release of CO_2_ under low temperature heating in a desorber. Fig. 10 illustrates these two major mine-based DAC process configurations.

#### 3.5.4. Equipment

The need for processing a large volume of air in a DAC application would result in large air contacting equipment and consequently high capital cost, not only for this technology but also in all other DAC technologies.^121,176^ Packed columns in either counter current or cross current configurations are often used as the air–liquid contacting equipment in amine-based DAC processes to date. Kiani *et al.*,^176^ proposed the use of a counter current conventional packed column as the gas–liquid contactor in their process. They reported that an absorber with the inlet area of 10.4 m^2^ and packing height of 4.4 m was needed to capture 50% of CO_2_ at the scale of 2.5 kt per a CO_2_ capture. The use of commercially available cooling tower packing was subsequently proposed as a way to reduce cost and energy requirement in such a system.^83,179^ Kiani *et al.*,^180^ tested their selected absorption liquid in various gas–liquid contactors, including packed columns with random and structured packings, and a cooling towers with two different packings. Relatively low mass transfer rates of CO_2_ into the solution were obtained in the cooling tower compared to other contactors, however, the gas side pressure drops were substantially lower for the cooling tower, helping to reducing the energy requirement of this system.^180^ Cross flow packed columns were also considered in a few amine-based DAC studies. Kasturi *et al.*,^184^ developed a gas–liquid contactor in cross current flow for DAC named HiDAC, using random packings between the layers of structured packing. Compared to other contactors, they reported this configuration has a higher specific surface area (885 m^2^ m^−3^), lower pressure drop (36 Pa at 1 m s^−1^ gas velocity), high wettability, and low corrosion, while being inexpensive and light. They reported a captured 21 kg of CO_2_ per m^3^ per day using 3 M potassium sarcosinate in a contactor with a volume of 0.027 m^3^. More recently, researchers looked at the use of membranes as the medium for contact between air and absorption liquid, showing that more than 83% of CO_2_ can be captured using an aqueous solution of glycinate amino acid salt.^185,186^

#### 3.5.5. Degradation

It is expected that degradation of the absorbent remains a significant concern for amine-based absorption liquids under DAC conditions, due to the use of heat for absorbent regeneration (thermal degradation) and continuous contact with a large volume of air (oxidative degradation). This is a major concern as volatile degradation products can be emitted to air if not suppressed or emission is mitigated. Amongst 6 amino acid salts investigated by Kiani *et al.*,^181^ only one was reported to pose high thermal and oxidative stability. Hence, any potential amine-based DAC system needs to use absorbents that pose high resistance to thermal and oxidative degradation and provide elevated CO_2_ mass transfer rates compared to current commercial offerings and MEA. In their recent work,^181^ they showed that the salts of proline and sarcosine have high potential to be considered in amine based liquid DAC, ideally for the processes with slightly lower regeneration temperature than 120 °C. Hastings *et al.*,^189^ studied the thermal degradation of two salts of taurate in terms of degradation extent and products, showing negligible degradation of these compounds at temperatures below 100 °C. Using the phase change DAC process reported by Custelcean *et al.*,^183^ the amino acids are never exposed to high temperature, hence the stability to high temperature will become less important.

#### 3.5.6. State of development and deployed plants

Most of the studies in this space still concern low TRL research, aiming to test and develop new absorbents and gas–liquid contactors for DAC. To date, the most advanced thermally regenerated amine-based DAC technology is developed by the commonwealth scientific and industrial research organisation (CSIRO) in Australia. The technology named the ambient CO_2_ harvester (ACOHA), is based on the use of a selected amino acid salt solution in a commercially available cooling tower.^190^ The technology readiness level (TRL) was quoted to be 4. Following this development, Rolls Royce and CSIRO partnered to build and demonstrate the technology at a scale of at least 100 t per year CO_2_ in the “environmental CO_2_ removal” (ENCORE) project under a UK government funding program. The pilot plant demonstration is located at the Rolls Royce site in Derby, UK, and front-end development started in 2022 with commissioning in 2024. The technology was expected to have reached TRL 6 upon completion of the project in 2025,^20,191^ but Rolls Royce ceased the pilot plant development after a strategy change. In their phase 1 project report, Rolls Royce and CSIRO reported that the energy requirement target for a final DAC product based on their technology would be less than 1 MWh per tCO_2_ (3.6 GJ per tCO_2_). Additionally, less water loss than other liquid-based DAC process is predicted by using the unique property of their amine-based absorption liquid.

#### 3.5.7. Energy consumption

Little information is available in the scientific or grey literature on electricity and heat requirements for amine-based DAC processes. Kiani *et al.*,^176^ report that the modelled heat required to regenerate an MEA solution was found to be around 3 MWh per tCO_2_ and considered to be almost similar for amino acid salt-based DAC as well. The electricity required for moving liquid and air in this system was estimated to be around 1.4 MWh per tCO_2_. The heat requirement can be reduced if one considers the integration of DAC with waste heat sources, as it requires low temperature heat for regeneration (100–120 °C). In another study,^179^ it was shown that the electricity requirement can be reduced to 0.308 MWh per tCO_2_ using the intermittent liquid flow that carbon engineering also suggested as a way to reduce the liquid pumping energy, and use of cooling towers that allow low pressure drops. Also, they showed that the heat requirement can be halved by integrating the CO_2_ air capture with methane production, using heat released from the methanation reaction. In Sabatino's study on the use of MEA for DAC, the energy consumption was quoted to be in line with the estimation of energy by Kiani, at similar operating conditions.^83^ More recently, research suggested that the desorption of CO_2_ can be accelerated using catalytic solvent regeneration, resulting in lower energy and temperature requirements for the regeneration process.^192^ It is unclear, however, as to what extent this will reduce the energy requirement. In the phase change DAC process discussed earlier, it was reported that the regeneration energy comprising the sensible heat and the heat required for releasing CO_2_ and water was 7.0–8.2 GJ per tCO_2_ (1.9–2.3 MWh per tCO_2_).^183^

### 3.6. Mineral looping

Mineral looping for DAC entails the binding of CO_2_ to metal oxides and hydroxides to form carbonate materials. The metal oxide and hydroxide sorbent material is regenerated using a high-temperature step to calcine the carbonate materials, in conjunction with a carbon capture and storage system, which results in high-purity CO_2_ for downstream compression, transportation, and storage.^193–195^ The metal oxides and hydroxides react spontaneously with CO_2_ in the atmosphere, leading to this process being achievable at atmospheric temperatures and pressures. Although quite a few metal oxide and hydroxides can be used to capture CO_2_ from the atmosphere, the two most covered in the literature are magnesium (MgO and Mg(OH)_2_) and calcium (CaO and Ca(OH)_2_).^193–195^ The only deployed DAC plant that uses the mineral looping system has been deployed in Tracy, California, by Heirloom Carbon, and it uses Ca(OH)_2_ as the adsorbent material.^195^

#### 3.6.1. Major challenges

The idea of using mineral looping as an approach to DAC was first tested by Erans *et al.* (2020), when they experimented with ground (100–500 μm) calcium oxide (CaO) and calcium hydroxide (Ca(OH)_2_) in shallow beds (∼5 mm) to determine if it would uptake CO_2_ from the atmosphere in indoor and outdoor environments, then be cycled through a high-temperature step to regenerate the capture medium.^194^ They were able to validate that indeed, both feedstocks did uptake CO_2_ from the atmosphere, forming CaCO_3_, and it was able to be regenerated *via* a calcination step to be reused for additional CO_2_ uptake. In their findings, they explore the diffusion and kinetic limitations of carbonation related to the relative humidity of the surrounding environment and the bed depth of the sorbent material. It was illustrated in their results that as the dry CaO samples were hydrated, forming Ca(OH)_2_, the kinetics of the carbonate formation increased. However, it was also stated that as the top layer of the material results in a build-up of CaCO_3_, the overall reaction then becomes diffusion limited due to the already reacted, passivation layer.^194^ This illustrates the benefits of shallower bed depths and the limited return from deeper bed depths. Provided that bed depth is a critical parameter to ensure the full reactivity of the capture sorbent, challenges may arise when designing infrastructure that optimizes for a high amount of surface area, as needed for DAC contactors.

Furthermore, with the carbonation reaction being one that is surface limited, by the interactions of CO_2_ and CaO or Ca(OH)_2_, increased surface area of the material is a key driver in the carbonation rate. Literature indicates that one way the surface area of the capture material is being maximized in stagnant beds is through grinding. In the experiments conducted by Erans *et al.* (2020), the particle sizes used ranged from 100–500 μm, yielding a carbonation efficiency of ∼75%^194^ and in the technoeconomic assessment carried out by McQueen *et al.* (2020), the particle size for the system was estimated to be 20 μm, estimated to yield a 90% carbonation efficiency.^193^ Grinding rocks to this particle size require crushing from 1000s of millimetres to ∼5 mm, then milling to achieve sizes smalling that, in which, milling is the more energy-intensive of the two operations.^196^ In addition to the energy required for reducing the capture material to these smaller sizes, rock powders at these sizes can pose respiration risks when dispersed throughout the air. This then demands that any DAC approaches utilizing rock powder as a capture agent design air contactors, so the capture agent is making adequate contact with the air but reducing the environmental losses of their material. This measure is well-aligned with business goals, because if environmental losses were increased, the operating cost of acquiring replacement capture material would also rise.

Lastly, to regenerate the capture material (CaO or Ca(OH)_2_) once it is carbonated (forming CaCO_3_), it must be calcined at temperatures close to 900 °C. Achieving these high temperatures at the industrial scale is yet to be reliably decarbonized. However, in literature where cycling CaO/Ca(OH)_2_ and CaCO_3_ are being discussed as a method for DAC, this high-temperature step is proposed to be met with electric kiln technology. Electric kiln technology for the purpose of calcining limestone is already on the market today,^197^ but their design often restricts the throughput of the material that can be achieved. Other entities are trying to address this by using direct separation technology^198^ or by using oxycombustion kilns fired with natural gas equipped with carbon capture and storage.^105^

Another potential challenge of deploying a mineral looping for DAC system, is aligning the continuous processes, specifically the calcination for regeneration, with the batch processes, such as the carbonation cycle of the material. In this same vein, mineral looping for DAC deployments will require decarbonized calcination processes to maximize the net CO_2_ removal, which may require point-source carbon capture systems or innovative hydrogen or renewable-driven high-temperature reactor systems.^197^

#### 3.6.2. Materials

The idea for using enhanced rock weathering to dispose of CO_2_ waste was first proposed by Walter Seifritz in 1990, where he illustrated that silicate minerals could be exposed to aqueous CO_2_, resulting in divalent cations (Ca^2+^ or Mg^2+^) and carbonic acid, and silica.^199^ The cations and the carbonic acid result in precipitate carbonates (CaCO_3_ or MgCO_3_). The mineral looping DAC approach is driven by the chemical reaction between metal oxides and hydroxides and CO_2_ in the atmosphere. However, metal oxides and hydroxides do not exist naturally, so must be sourced from either silicate or carbonate feedstock. When these oxides and hydroxides are sourced from silicate minerals, an extraction step is required to separate out the calcium and magnesium species from the silicate structure, so the calcium and magnesium oxides can be used for CO_2_ capture. When these oxides and hydroxides are sourced from carbonate minerals, they must undergo the calcination step to drive off CO_2_, replenishing the reactive oxide state for future CO_2_ uptake. This calcination step is the same that is used to regenerate these materials after the capture step has taken place.

When the calcium (or magnesium) carbonate is calcined, the resulting species are CO_2_, which is compressed for further downstream processing and calcium (or magnesium) oxide. This oxide can then be hydrated to form calcium hydroxide (Ca(OH)_2_), which has better CO_2_ capture kinetics.^194^ This hydration step requires water, predominantly in the form of steam.^194^

It is possible that the decision to utilize the calcium carbonate system in favour of the magnesium carbonate system is due to the hydrophilic nature of MgO and MgCO_3_. When magnesium hydroxide (Mg(OH)_2_) is carbonated at atmospheric pressure, this can result in a hydrated state of magnesium carbonate, MgCO_3_·3H_2_O, or nesquehonite.^200^ The added presence of water to the resultant product can lead to additional considerations regarding materials handling, process equipment, and energy requirements for the system. For example, the energy requirements for calcination, when the resulting carbonation state is nesquehonite, will be increased due to the energy required to drive off the water in the higher hydration state.

#### 3.6.3. Processes

The mineral looping approach to DAC is being pioneered by a few startup companies, including Heirloom Carbon Technologies, 8 Rivers Calcite, and Origen Carbon, of which, Heirloom Carbon Technologies has the most public literature available. Each of their methods centres around the same principal process. Calcium hydroxide (Ca(OH)_2_) is used to capture CO_2_ from the atmosphere, forming calcium carbonate (CaCO_3_). The resulting calcium carbonate is sent through a high-temperature reactor where it is calcined into calcium oxide (CaO) and CO_2_. The CO_2_ is separated from any other gasses in the gas stream and compressed for downstream processing. The calcium oxide is then hydrated, reforming calcium hydroxide, which can be reused as the CO_2_ sorbent.

The high-temperature step for calcining calcium carbonate takes place at 900 °C, which is usually conducted in a kiln.^195,201,202^ Both Heirloom Carbon Technologies and Origen Carbon indicate that their kiln technology is powered *via* renewable energy or can be flexible to the fuel that is utilized to achieve the required high-temperature environment.^195,202^ Provided that both the carbonation and the slaking steps are exothermic, with large enough material throughput, there may be opportunities for heat integration. One method may be utilizing the waste heat from the slaker to preheat the carbonated material before it enters the calcination kiln.

Based on the ability for Ca(OH)_2_ to naturally uptake CO_2_ when laid out in small bed depths,^194^ it may be possible for mineral looping for DAC to be done completely passively rather than with forced air flow.^195^ It should be noted that the concept of passive carbonation, specifically relating to mineral looping for DAC is not well defined within the literature, so claims of passive carbonation by any company operating in the space may be referring to the lack of a single aspect that accelerates this process or a host of aspects that have the potential to accelerate this process. Both 8 Rivers Calcite and Heirloom accelerate the carbonation process with the assistance of fans to overcome low pressure drop across the contactor geometry.^201,203^ Origen Carbon on the other hand, has indicated that their low-intensity air contactor is optimized for passive carbonation, which may mean that no fans are required.

The air contactor system that Heirloom has developed is a system of vertically aligned trays that contain small bed depths of Ca(OH)_2_. There are fans that assist ambient air flow over these trays to facilitate carbonation. The vertical tray-based design has the potential to utilize warehouse automation techniques to optimize and automate materials handling.^204^ Based on this system, it is estimated that Heirloom can achieve 85% carbonation before regenerating the sorbent material through the calcination step. At 85% carbonation, the carbonation rate for this system is 630 gCO_2_ m^−2^, which can be achieved in nearly 3 days.^195^ Based on the preliminary designs that have been mocked up by Origen Carbon, their contactors appear to resemble circular cooling towers, while 8 Rivers Calcite's design resembles conventional rectangular cooling towers.^202,205^

#### 3.6.4. State of development and deployed plants

To date, Heirloom Carbon Technologies has the only commercial DAC plant that operates using the mineral looping process. Their DAC plant is located in Tracy, California, USA and has been in operation since November 2023.^203^ This plant has the capacity to capture 1000 metric tons of CO_2_ per year and the CO_2_ captured from the atmosphere will be permanently sequestered in concrete through Heirloom's partnership with CarbonCure Technologies.^206^

Since this deployment, Heirloom has also been awarded contracts with the U.S. Department of Energy's Office of Clean Energy Demonstrations to establish the first DAC Hub in Louisiana, Project Cypress, alongside Climeworks.^207,208^ The ultimate goal of Project Cypress is to capture 1 MtCO_2_ per year at full capacity, of which, Heirloom has announced that they will be supplying 360 000 tCO_2_ per year.^207,209^ Origen Carbon is one of a few DAC technologies chosen to collaborate on the community alliance for direct air capture (CALDAC) DAC Hub award, which is focused on evaluating three different sites for developing community-centred DAC deployments. The locations under consideration for this study include South San Joaquin Valley, near Fresno, CA, and nearby Bakersfield, CA.^210^

#### 3.6.5. Energy consumption

The mineral looping for DAC process has three fundamental, energy-consumptive processes, (i) blowing air over the capture sorbent, (ii) calcination to regenerate the CaO, and (iii) hydrating CaO to form reactive Ca(OH)_2_. Of the public literature available regarding the mineral looping process for DAC, conservative estimates for calcination with a magnesium-based system, in which anhydrous magnesium carbonate (MgCO_3_) is formed, are 5.9–8.0 GJ per tCO_2_ with a 90% calcination efficiency and 90% kiln efficiency.^193^ These were estimated provided two different scenarios, where a lower temperature, longer calcination cycle (600 °C for 2 h) was used and a scenario with a higher temperature, shorter calcination cycle (1200 °C for 0.5 h).^193^ Due to the reality that anhydrous magnesium carbonate is not often the product, but rather nesquehonite (MgCO_3_·3H_2_O), the actual energy requirements for a magnesium-based system are expected to be higher because the water must first be evaporated. Despite the high temperatures required for calcining calcium carbonate (∼900 °C), the energy requirements are expected to be less than that for a magnesium-based system due to the absence of a hydration state in the carbonate product. If thermal energy from the exothermic reactions that take place in the air contactor or the slaker are able to be utilized, the primary energy requirements for the calcination step are likely to decrease because, at a minimum, the material entering the calcination kiln may be able to be preheated *via* this heat recovery.

The air contactors require electricity to run the fans, which consume <0.05 GJ per tCO_2_ and the calcination for CaCO_3_ alone, requires 4 GJ per tCO_2_, not considering any energy losses or heating of the material to calcination temperature.^195^ Heirloom aims for their process to require less than 5.4 GJ per tCO_2_ heat (1500 kWh per tCO_2_) at scale.^195^

### 3.7. Membrane-based DAC

#### 3.7.1. Introduction

The dynamic and rapidly growing field of gas separation by membranes offers energy-efficient and cost-effective solutions for industrial applications, benefiting from advantages such as simplicity and compactness. Membranes can selectively permit gases based on size, solubility, or affinity. Moreover, membrane processes lend themselves to modular integration, enabling decentralized applications and customized solutions for diverse separation needs. In the field of carbon capture, the Polaris membrane by MTR industrial separations was the first commercially available membrane. Delivering a demonstrated performance of 1000 GPU (1 GPU = 7.5 × 10^−12^ m^3^ (STP) m^−2^ s^−1^ Pa^−1^, STP: standard temperature and pressure) alongside a CO_2_ to N_2_ selectivity of 50, it emerges as a solution holding potential for point source CO_2_ capture applications.^211^ However, when applied to lower-concentration scenarios like ambient air, distinct demands for the membrane arise. Elevated permeance and selectivity are requisite for efficient DAC, as detailed by Fujikawa *et al.*^212,213^ Furthermore, variations in gas compositions, including heightened oxygen concentrations and humidity, necessitate tailored membrane properties for DAC.

#### 3.7.2. Major challenges

One major difficulty in developing membrane DAC technology includes the novelty of the technology. According to Fujikawa *et al.*, permeances of 10 000 GPU and a CO_2_/N_2_ gas selectivity of larger than 30 are required to obtain a CO_2_ purity of 40%.^212^ As these membranes do not exist so far, many researchers focus on decreasing the membrane thickness to improve the material properties. This is however challenging the mechanical stability and an upscaled fabrication. Contrary to post-combustion CO_2_ capture, the CO_2_/O_2_ selectivity also determines the applicability of a membrane for membrane DAC which was rarely studied so far.^30,214,215^

#### 3.7.3. Materials

To characterize a membrane's performance for gas separation, permeance and selectivity are used. Gas permeance denotes the ease with which gases traverse membranes. It is determined by the material's permeability and membrane thickness. Hereby, permeability combines solubility and diffusivity, with the former characterizing a material's gas dissolution capacity, and the latter its gas transport capability. Gas selectivity, achieved through gas-selective layers, is especially important in DAC due to low concentrations of the targeted CO_2_. For CO_2_, selectivity is primarily influenced by its solubility in the material, meaning the transport can be facilitated with the incorporation of specific CO_2_ carriers aiding its passage while impeding others.

Current research is focusing on the development of thin film membranes of highly selective materials.^216,217^ These membranes, characterized by reduced thickness, hold the only potential to achieve enhanced gas permeance for a certain material. Thin film membranes are composed of a thin selective layer of polymer or other materials with specific functional groups that interact with the target gas molecules. The selective layer is either coated on a dense gutter or coated with a protective layer. The thin film nature of these membranes allows for efficient transport of gases and is often essential for achieving the desired separation performance. For mechanical stability, an additional porous support is used. Ariyoshi *et al.* built a nanomembrane composites of poly(dimethylsiloxane) and cellulose nanofibers with a permeance of 10 000 GPU and a CO_2_/N_2_ selectivity of 11.^218^ Yoo *et al.* fabricated a defect-free Teflon-based membranes with a CO_2_ permeance of ∼31 500 GPU and a CO_2_/N_2_ selectivity of 3.3.^219^ Additionally, Fujikawa *et al.* developed freestanding siloxane nanomembranes boasting CO_2_ permeances exceeding 40 000 GPU with a CO_2_/N_2_ selectivity of 11.^213^ With reducing the membrane thickness, however, only the permeance can be improved, but not the selectivity. An overview of the performance of polymer membranes for the suitability of direct air capture is listed in the review paper by ref. 30. However, most membranes were only tested for pure gas separation and not explicitly for direct air capture conditions.

Contrary to polymer membranes, facilitated transport membranes (FTMs) use solution-diffusion and carrier mediated mechanisms to increase gas permeation and selectivity. They incorporate complexing agents as carriers, reacting reversibly with feed gas components. Enhanced by carrier-mediated diffusion, FTMs exhibit high permeability and selectivity, especially for CO_2_ separation under low feed pressures, and are therefore another route towards effective membranes for dilute CO_2_ removal. Hoshino *et al.*^220^ used amines to facilitate the CO_2_ transport and fabricated defect-free amine-doped microgel nanomembranes and achieved CO_2_/N_2_ selectivities higher than 2000. Due to the increased selectivity, a higher purified CO_2_ can be produced with up to 95%.^220^

#### 3.7.4. Processes

**Fig. 11:**
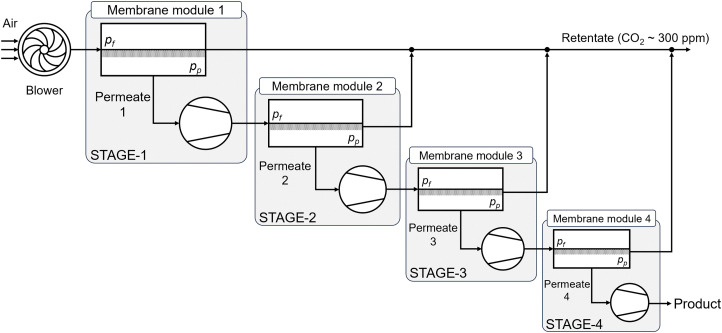
Scheme of the four-stage membrane process for CO_2_ by Fujikawa *et al.* Redrafted from ref. 212.

Membrane-based DAC is a modular approach and thus allows for decentralized capture. So far, only simulation-based process performance has been published. Fujikama *et al.*^212^ revealed that single-step separation processes are inadequate for achieving higher CO_2_ concentrations. In a four-stage process (Fig. 11), with inlet CO_2_ concentration at 400 ppm and outlet at 300 ppm, representing preindustrial levels, they studied a membrane with 10 000 GPU. To obtain CO_2_ concentrations >10%, a selectivity >30 and a pressure ratio between the feed and the permeate side >30 is required.^212^

Alternatively, identifying suitable hybrid processes that combine membrane separation with adsorption or absorption can be advantageous. This approach could address challenges such as selectively separating oxygen from air, a task that can complicate conventional absorption due to solvent oxidation. Combining membrane separation and absorption can streamline the process, requiring less energy by minimizing the volume of gas needing to be pumped through the liquid phase.

#### 3.7.5. State of deployment and deployed plants

The technology of m-DAC is novel and is estimated to be at TRL 1–2. The membrane properties still need to be improved to be used in an upscaled DAC application. So far, there are no deployed plants.

#### 3.7.6. Energy consumption

For m-DAC, the required energy is limited to vacuum pumps only. The first stage with the lowest CO_2_ inlet concentration requires the largest amount of energy with 11.6 kWh per kg CO_2_ per day with a pressure ratio of 25 whereby four stages in total only require 14.2 kWh per kg CO_2_ per day.^212^ Thus, higher CO_2_ concentrations in the feed significantly reduce the energy needed for CO_2_ capture while simultaneously increasing product purity. Skipping the first stage, thus, could tremendously reduce the energy requirement. As the average CO_2_ concentration in office spaces reaches nearly 1000 ppm, they could serve as viable small-scale m-DAC capture site, especially when integrated with air conditioning systems. For instance, Fujikawa *et al.* estimates that approximately 4200 tons of CO_2_ per year could be collected from just one building, such as the Roppongi Hills Mori Tower Building in Tokyo. This also highlights the potential for substantial CO_2_ capture from concentrated air sources, offering opportunities for efficient carbon capture and utilization strategies within urban environments.^212^

### 3.8. Cryogenic direct air capture

In a seminal direct air capture conference paper from Lackner *et al.*, the heat needed to be removed from air to extract a tonne of CO_2_ in the form of dry ice is 50 GJ. Using a coefficient of performance (COP) of 2, which is roughly the theoretical maximum for the temperature difference between solid CO_2_ and ambient (∼15 °C), then 25 GJ t-CO_2_^−1^ of electricity would be required as a theoretical minimum. In reality, inefficiencies in the system would lead to the real electricity requirement being much higher.^79^ Von Hippel explored the performance of a cryogenic DAC system sited in Arctic regions utilising radiative cooling *via* the constant night sky in winter months.^80^ In the Antarctic, it was assessed that the energy demand could be as little as 2 GJ t-CO_2_^−1^ of electricity or 5 GJ t-CO_2_^−1^ in Yukon. However, this system would only be able to run during the winter months, meaning capital costs will need to be minimal to make this approach feasible. Meanwhile, in the Arctic regions suggested, there are also questions over access to clean electricity, CO_2_ storage, and a labour market.

Beyond this, the literature on cryogenic DAC is extremely limited which is possibly due to major challenges around energy demand, availability, and infrastructure highlighted above. As a result, the technology readiness level is very low^1^ and more work needs to be done if cryogenic DAC is to be considered as an option.

### 3.9. Materials discovery for DAC

For many of the technologies discussed in Section 3, materials engineering has been a significant part of the research efforts. The quest for the “optimal” or “best” carbon capture or DAC material has become one of the key questions and objectives of the R&D community over the past years. The promise of finding such a material is dictated by the definition of “optimal”, which is normally translated into one or several metrics, referred to as key performance indicators (KPIs). Material screening studies have been reported in the literature aiming to identify top-performing materials for different carbon capture applications. The ‘workflow’ for a DAC screening study holds several similarities to studies targeting separation from feeds with higher CO_2_ concentration. Such studies comprise the evaluation of KPIs at different levels of complexity or detail, all the way from very simple metrics at the material level, (*e.g.* CO_2_ uptake, selectivity) to much more complex metrics at the technoeconomic or environmental level, and the identification of “best” candidate structures. This section discusses trends distilled from a wide set of papers (∼60) which cover mainly screening studies for CO_2_ separation processes from point-sources, highlighting additional considerations relevant for DAC. This is required as studies which specifically target DAC separation are highly limited.^66,221–227^

**Fig. 12:**
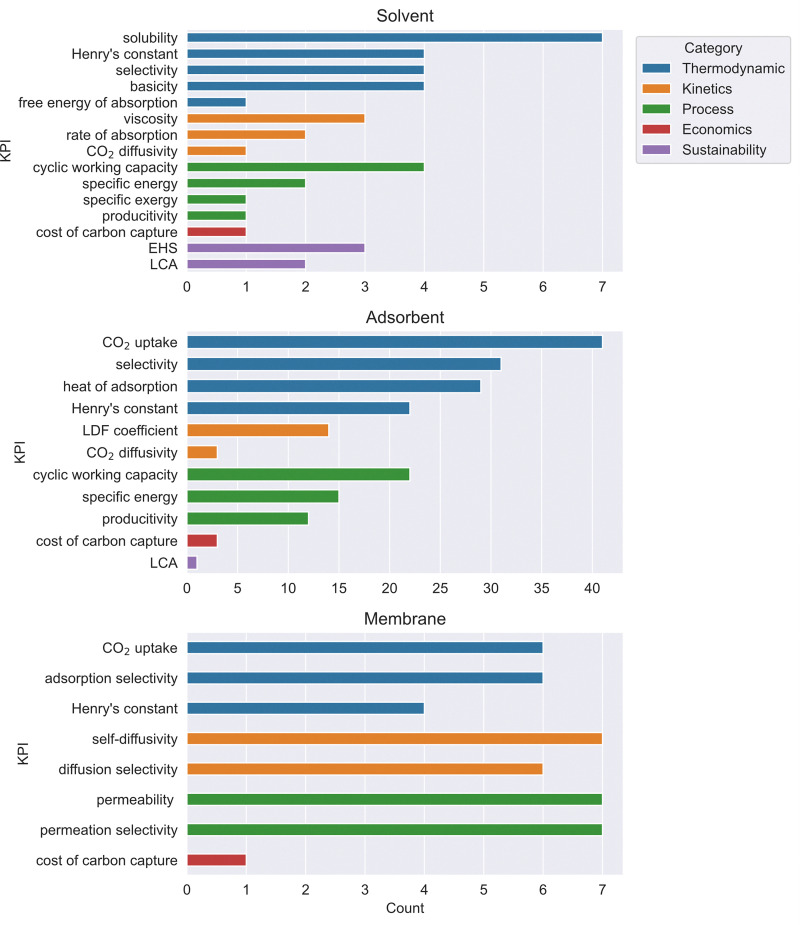
Materials screening KPIs reported for three different capture technologies: solvent-, sorbent-, and membrane- based.

We surveyed 15 papers on liquid-based absorption,^221,222,228–237^ 40 papers on adsorption-based CO_2_ capture,^66,223–227,238–270^ and seven papers on membrane-based CO_2_ capture.^271–277^Fig. 12 categorises KPIs mentioned by these materials-screening studies into several areas – thermodynamics indicators, kinetics indicators, process-level indicators, as well as economics- and sustainability indicators.

Among these papers, thermodynamics and kinetics indicators were the most abundant, followed by process-level indicators. Higher level economics and sustainability- related indicators were the least represented.

**Fig. 13:**
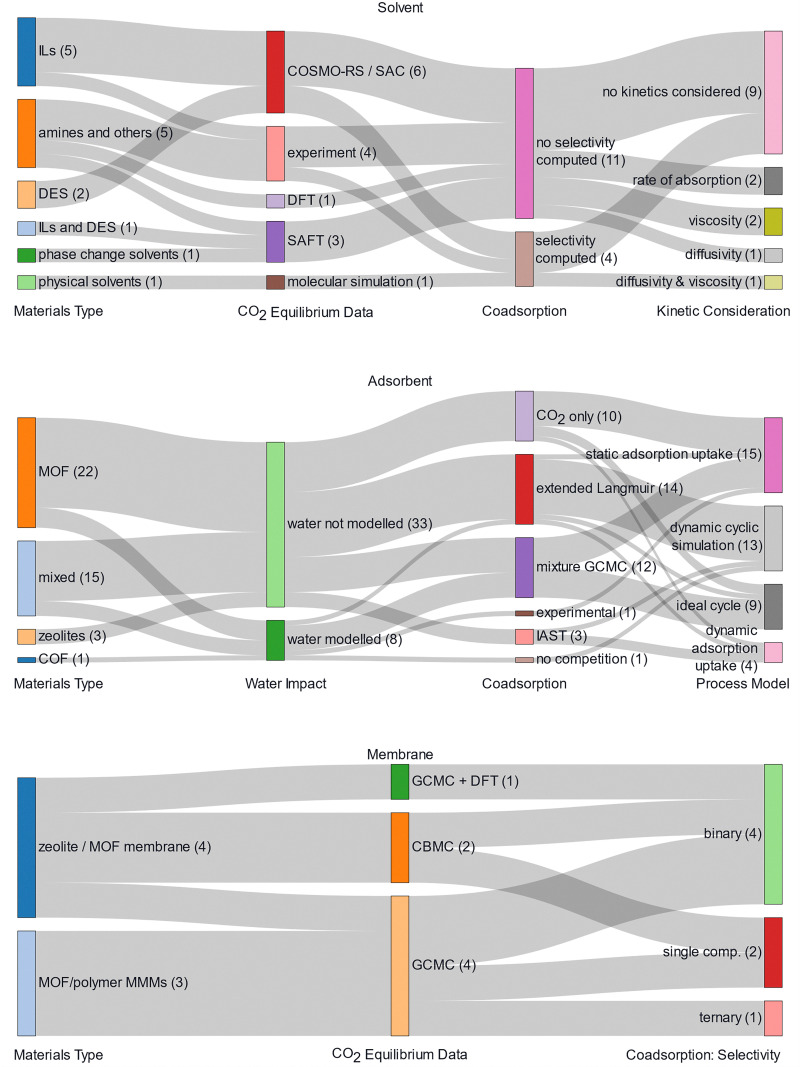
Modelling approaches and considerations for three different capture materials: solvents, adsorbents, and membranes.

For liquid-based absorption, 8 papers considered ionic liquids (ILs) and deep eutectic solvents (DESs), 5 papers considered amines, and only 1 study each mentioned physical solvents and phase change solvents (Fig. 13a, column 1). For solid-based adsorption, 22 studies operated on a dataset of exclusively metal–organic frameworks (MOFs), and 14 studies utilised hybrid databases that are primarily composed of MOFs but also contain zeolites, and zeolitic imidazolate frameworks (ZIFs, a subclass of MOFs) (Fig. 13b, column 1). In addition, 3 studies considered exclusively zeolites, and 1 study considered covalent-organic frameworks (COFs). For membrane capture, 4 studies considered polycrystalline/film-based membranes composed of either zeolites or MOFs (Fig. 13c, column 1). The remaining 3 studies considered mixed-matrix membranes composed of a mixed phase of polymer and MOF.

The types of materials emphasised in the screening studies do not necessarily reflect the popularity of materials deployed in practice. In the case of solid materials (adsorbents and membranes), we observed a strong preference for crystalline materials. The structural information for zeolites,^278^ MOFs,^279,280^ COFs,^281,282^ and subsets thereof, such as 2D zeolites^283^ and fluorinated MOFs^284^ are available in data repositories. We also observe a general preference for structural modularity, as exemplified by ILs (molecular compounds with paired ionic motifs), DESs (mixtures involving paired hydrogen bond donors/acceptors), and reticular framework materials (MOFs and COFs). This preference in screening studies likely reflects feasibility considerations inherent to *in silico* materials discovery, where the available chemical design space, along with the accuracy and computational expense of property and performance predictions, play a crucial role in determining which materials are explored.

The preference for *in silico* performance evaluation is apparent when considering the most computed indicator, the unary CO_2_ loading at the feed condition. For solvents, 11 of the 15 studies rely on computational estimation of the CO_2_ loading (Fig. 13a, column 2). Predictive quantum chemical calculations with conductor-like screening models (COSMO-RS and COSMO-SAC) are adopted to predict physisorption equilibrium properties, while the statistical associating fluid theory (SAFT) equation of state and density functional theory (DFT) can be used for the systems that involve chemisorption.^221,228,235^ For solid materials discussed in Fig. 13b and c, estimation of unary sorbate loadings is well-established *via* the grand-canonical Monte Carlo (GCMC) approach.^238,250^ However, predictive accuracy decreases for systems with strong adsorption nonidealities, such as site heterogeneity (*e.g.*, electrostatic interactions in zeolites), sorbate clustering effects (*e.g.*, condensation phenomena in water adsorption), and reactive capture mechanisms (*e.g.*, amine-carbamate formation in solids).

Beyond unary equilibria, the importance of evaluating co-adsorption equilibria is significant for DAC given that most components in the air feed occur at significantly higher concentrations than CO_2_.^285^ For liquid-based absorption, only four of the 15 studies reported a selectivity indicator (Fig. 13a, column 3). For solid adsorption, 23 out of 40 studies report a binary selectivity and 6 more report equilibrium information for a ternary CO_2_–N_2_–H_2_O system (Fig. 13b, column 2 & 3). Predictions for binary CO_2_–N_2_ equilibria on adsorbents are realized by applying the ideal adsorbed solution theory (IAST) to unary equilibrium information.^286^ The application of IAST to the so-called Langmuir isotherm model yields a co-adsorption uptake equation explicit in temperature and pressure, which is advantageous for repeated computations.^287^ The applicability of IAST is limited for H_2_O-containing systems though. Therefore, mixture co-adsorption equilibria is directly computed by GCMC,^251,259,288,289^ or experimentally measured.^290^ Due to increased computational and/or measurement effort to derive sorption equilibria for humid systems, less than 20% of the papers account for water impact and such evaluations are performed for only a reduced subset of the original scope of sorbents. For membrane-based capture, four of the seven studies report a binary equilibrium selectivity and 1 more reports ternary equilibrium (Fig. 13c, column 3).

Information regarding the process operation can strongly influence comparisons between different materials. For example, the choice of regeneration temperatures informs the residual capacity of CO_2_ retained in the ad/absorbent, directly affecting the working capacity and process energy consumptions. Liquid and solid sorbent capture processes rely on similar metrices for performance evaluation (Fig. 12a and b). Among the 21 solid adsorbent screening studies that discuss the cyclic working capacity, respectively, eight derive the indicator by applying idealized mass and energy balances on specific operating points of the process, while the remaining 13 studies apply transient numerical modelling. Transient modelling allows the incorporation of kinetic and transport phenomena into the capture performance. Representative studies which undertake the evaluation of process KPIs by cyclic process simulations include.^245,246,249,253,258^ Given the difficulties in computing H_2_O equilibria, these studies incorporate only the CO_2_/N_2_ equilibria (Fig. 13b, column 4). Optimization of process operating variables is required to compare materials at their respective best-performing configurations. A single evaluation of an optimized process KPI may require several computing days or hours. Therefore, the initial materials pools are narrowed down considerably before such optimisation analyses, which typically cover tens of materials.

Although kinetic limitations can reduce the efficiency of a separation, less than half of the solvent and adsorbent studies mention kinetic indicators (Fig. 12). The kinetics indicators reported for solvents include viscosity, diffusivity, and rate of absorption,^222,228,229,231,237,291^ and their origin can be experiments, thermodynamic models, or molecular dynamics simulations. For adsorbents, the synthesized crystals typically need to be shaped into macroporous beads or pellets or coated onto a monolith or laminate to have the required mechanical strength and mass transfer properties for the processes. The kinetic performance is therefore largely dependent on the macrostructures, and assumptions of relevant structural properties such as pellet size and porosity are needed for the theoretical estimation of kinetic parameters to be used in process modelling. The mostly used kinetic indicator for adsorbents is the linear driving force (LDF) constant, while a few papers use the more detailed CO_2_ diffusivity. For membranes, gas transport constitutes an integral element of the process performance. The membrane-related studies focus on permeability and selectivity as the key process KPIs, though the level of complexity can vary. Gas diffusivity is an essential element for permeability calculation that can be obtained with molecular dynamics simulation. Most basic cases use ideal selectivity calculated at infinite dilution and with single component equilibrium data, while more realistic cases derive selectivity using binary or ternary permeability.

High-level economic and life-cycle assessment (LCA) KPIs integrate process and engineering design choices to offer interpretability to decision-makers. For solvents, only one study includes simple cost estimation for ionic liquid-based DAC processes,^222^ while a few cases report sustainability KPIs including environmental, health, and safety (EHS) impacts and/or LCA KPIs (*e.g.*, cumulative energy demand, global warming potential).^228,235,236^ For adsorbents, more detailed techno-economic assessments have been carried out.^248,266^ For example, Charalambous *et al.*^266^ developed an integrated technology platform for the holistic evaluation of sorbent-based carbon capture processes, which outputs a diverse set of process, economic and LCA KPIs.

The indicators covered in Fig. 13 traverse widely different length scales – from the atomistic scale in affinity and energetic predictions, to macroscopic plant or regional scales for economic and life-cycle predictions. The materials modelling workflow requires delicate compromises between materials scope, model complexity, predictive accuracy, and stakeholder relevance. Studies which attempt multiscale modelling share several features. First, due to the large starting dataset and the intensive computational efforts required to calculate detailed material properties, a multistage approach is often adopted. With increasing modelling complexity, progressively more advanced material properties are calculated for a smaller set of promising materials (*e.g.*, the number of pure component predictions is larger than the number of mixture prediction, is larger than the number of process modelling runs). Another trend is the reliance of machine learning (ML) models to accelerate expensive computations. ML approaches have been adopted to predict material properties such as basicity of ternary amines,^291^ molecular orbital (MO) energy levels and binding free energies of amines, alkoxides and phenoxides for DFT calculation,^221^ and CO_2_ solubility in deep eutectic solvents.^292^ Alternatively, ML strategies have been explored to predict expensive process indicators from material parameters and operating parameters (cycle step durations, velocities, pressure levels).^255^ Finally, a multiscale approach is sensitive to the propagation of uncertainties. Cleeton *et al.* demonstrated significant quantitative differences arising from force-field uncertainty on the Pareto fronts of process-level KPIs in a pressure-swing adsorption-based CO_2_ capture process.^257^ Therefore, ranking differences are anticipated to significantly benefit from validation with experimental data.

### 3.10. Summary of technology status, challenges, energy consumption, and path forward

#### 3.10.1. Summary of technology status

**Fig. 14:**
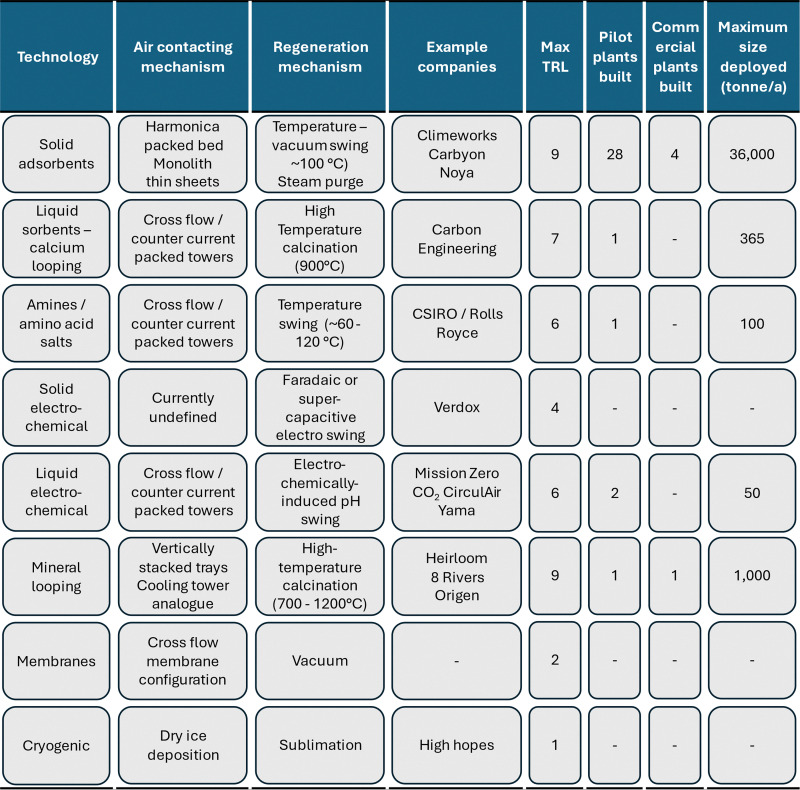
Summary of DAC technology types, air contacting and regeneration mechanisms, example companies and state of deployment.

Summarizing, there is a scala of direct air capture technologies in development or recently commercialised, with novel concepts being explored currently. Only solid adsorbent and mineral looping type technologies have reached commercial scale, mostly because they can be deployed commercially at small scales of 1 kilotonne CO_2_ captured per annum. Liquid absorbents with calcium looping are to follow in late 2025 and will mark the first deployment at the 100's of kilotonnes scale – if successful. Fig. 14 and Table 13 show that at least five technology types have made it to the pilot plant scale, which is a critical step in technology derisking. It has been argued that many more technology variants should follow to allow for a portfolio of DAC technologies that suit different situations and/or regions^17^ and to plot a credible path forward to bring the more promising of these technologies towards commercial deployment too.

The sections above highlighted that all DAC technologies face technical challenges, summarised in Table 7. Water (and absorbent) loss is a common one for the liquid systems, which can be mitigated by improved water management designs, *e.g.*, like the water washes in post combustion CO_2_ capture plants. Materials degradation and/or loss is a challenge for almost every technology, which should be one of the R&D foci for the coming years. Additionally, the very low TRL solutions need scaling and long-term testing under DAC relevant conditions at TRL 4 at least, preferably at the small pilot level. Finally, across all technologies integrated materials – contactor – process design optimisation is needed to increase productivity and drive energy and degradation down.

**Table 7:** Key technology challenges and possible remediations

Technology	Key challenges	Possible remediations
Solid adsorbents	– Low productivity	– Materials – contactor – cycle optimisation
	– Adsorbent degradation	– Materials engineering; cycle design
	– Ambient variability	– Materials diversification
Liquid absorbents – calcium looping	– Low cyclic absorbent capacity	– Absorbent – contactor – process optimisation
	– Water losses in contactor	– Improved water management design
	– Complex sorbent regeneration	– Alternative regeneration approaches
Liquid absorbents – amines/amino acid salts	– Absorbent and water loss	– Improved contactor and water management design
	– Low productivity	
	– Absorbent degradation	– Improved materials – process design
Solid electrochemical	– Novelty, unproven nature	– Integrate parts, scale to TRL 4–5
Liquid electrochemical	– High electrical resistance, overpotential	– Scale novel cell/cathode designs
	– Water losses in contactor	– Improved water management design
	– Materials degradation	– Materials engineering, cell design
	– Materials cost, supply at scale	– Trial cheaper materials, slowly scale supply chains
Mineral looping	– Bed deactivation, passivation	– Optimised bed/contacting designs
	– Reactivity *versus* materials loss	
Membranes	– Insufficient membrane permeance and CO_2_ selectivity to reach desired purity	– Materials research
		– Hybrid DAC approaches
Cryogenic	– Not energetically feasible	– Not applicable

#### 3.10.2. Synthesis of energy consumption

**Fig. 15:**
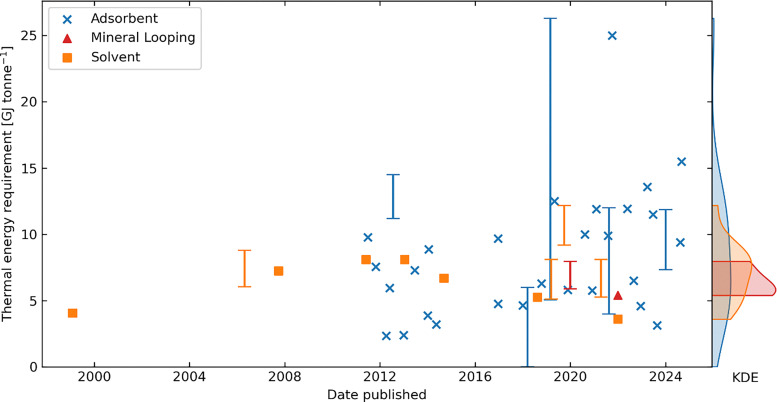
Comparison of DAC thermal energy requirements for liquid and solid sorbents and mineral looping, reported in the scientific literature over time. Markers are reported point estimates, lines are reported ranges. The kernel density estimates (KDE) on the right show the most reported thermal energy values.

Fig. 15 compares the above discussed thermal energy requirement for liquid and solid sorbents and for mineral looping. The figure shows that the most reported thermal energy ranges fall between 4 and 12 Gigajoule per tonne CO_2_ captured with outliers for solid adsorbents to 25 Gigajoule per tonne, while the lowest reported values, also for solid sorbents, are around 2 Gigajoule per tonne, depending on the adsorbent assessed. There is no observable downward trend with time, which may suggest that further research of the kind published in the scientific literature is not *per se* reducing DAC energy consumption. This can be because the scientific literature mainly reports laboratory obtained values for new materials or modelling results for existing processes, while there is scarcity of reported energy performance values for pilot and commercial plants and proprietary material–process combinations. It may well be that companies are ahead of academia in terms of process development and optimisation, as energy performance improvements often come from heat integration options, and use of smarter heating devices (*e.g.*, heat pumps), not *per se* materials development only (while this can contribute too).

**Fig. 16:**
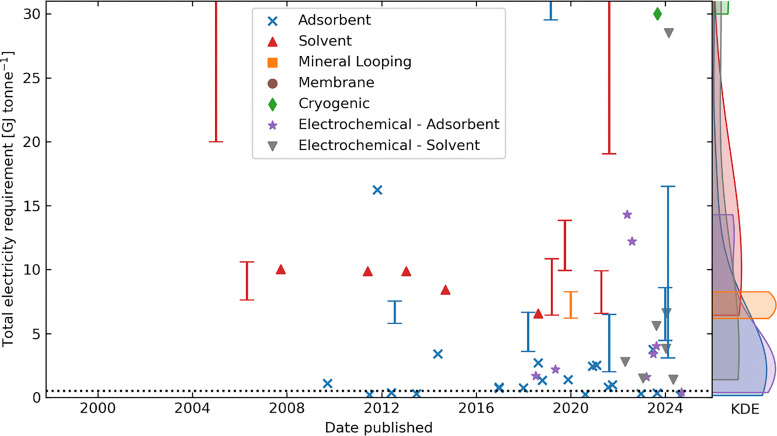
Comparison of DAC total electricity requirements, reported in the scientific literature over time. Note that thermal energy was converted to electricity to allow comparison of heat and electricity-driven DAC technologies. The technologies that use low-grade heat (80–120 °C) were assumed to be powered by an air source heat pump with a coefficient of performance of 2. Technologies that use higher temperatures were assumed to be powered by an electric boiler or kiln with a coefficient of performance of 1. Markers are reported point estimates, lines are reported ranges, the black dotted line is the minimum thermodynamic work needed to separate 50% of CO_2_ from ambient air and purify this to 99.5% (0.45 MJ kg^−1^). The kernel density estimates on the right show the most reported total electricity values.

Finally, Fig. 16 compares the total energy requirement of all reviewed technologies, when heat for regeneration is converted to electricity by assuming low temperature heat (80–120 °C) can be provided by air source heat pumps with a coefficient of performance of 2, while heat over 120 °C is provided by electric boilers with a coefficient of performance of 1. A couple observations from the figure include that cryogenic DAC really has the highest energy requirement with 30 GJ per tonne CO_2_ captured. This suggests a very low chance of cryogenic DAC ever becoming competitive. Additionally, membrane-based DAC appears less competitive, with ranges from 10 to 30 GJ per tonne CO_2_ captured, while achieving substantially lower than 95% CO_2_ purity. Because of the inherently efficient manner of generating low temperature heat *via* heat pumps, solid sorbent DAC appears more efficient than liquid absorbent DAC with mineral looping. Solid sorbents now roughly fall in the same energy consumption range as electrochemical technologies, while we note that most electrochemical values reported stem from lab measurements under highly idealised circumstances at very small scale. The modelling-based electrochemistry studies reveal a very different picture, namely that, at least the liquid absorbent plus electrochemical regeneration techniques show very high energy consumption from 8 to 28 GJ per tonne CO_2_.

The total electricity consumption observed in Fig. 16 also suggests a drastic reduction in energy consumption is yet to happen and recent studies appear to have increased the range upwards or, at least, have widened the reported range for energy consumption. This suggests sound understanding of the total energy consumption of DAC technologies is still lacking, at least in the public domain, and many more detailed studies for the different DAC technologies must be undertaken, contingent to the availability of materials performance data and ideally informed by pilot, demonstration, or commercial plant performance data.

A key remaining question is what a plausible energy consumption may be for future direct air capture plants. While studies targeting this exact question are lacking in the literature reviewed (up to August 2024), some reports may begin to give a direction. Wilcox, Psarras, and Liguori,^293^ for instance, calculated the minimum theoretical work for separating CO_2_ from the air and showed this lies between 0.44–0.48 GJ tCO_2_^−1^ (19.4–21.1 kJ mol^−1^) when achieving a concentrated CO_2_ stream of 99.5% purity, depending on which percentage of CO_2_ was captured from the air (see also the black dotted line in Fig. 16). Assuming a careful second law efficiency for future DAC plants of 10% (industrial fluid separation processes normally achieve less than 30% second law efficiency) implies the real work involved will approximate 4.5 GJ tCO_2_^−1^. Young *et al.* used another approach to estimate the future energy use for four DAC concepts by assuming that energy consumption ‘learns’ (see also Section 4.1.4 for the concept of technological learning) by 5%, equal to the operational cost learning rate of oxygen production from air.^294^ Using this approach, they found that the total work requirement including compression to pipeline pressure could reach 4.65 GJ tCO_2_^−1^ for liquid sorbent DAC with calcium looping, while may reach 3.0 GJ tCO_2_^−1^ for solid sorbent DAC. These two studies thus suggest that 3–5 GJ tCO_2_^−1^ of real work may be feasible long-term.

We also note that the energy consumption of the DAC technologies will add substantial demand to our (decarbonising) energy systems. For many DAC technologies this means low carbon electricity, implying renewable electricity generation capacity needs be built additional to the required clean electrons to decarbonise the other sectors of our economy. The impact of energy carbon intensity on DAC carbon removal is further discussed in Section 4.2.2.

#### 3.10.3. Path forward

Clearly, many direct air capture technologies are still at low maturity, while all DAC technologies still have substantial challenges to overcome in terms of materials stability, energy and resource consumption, and integrated materials – contactor – process designs. Low cost DACCS may only become a reality if these challenges are solved in an integrated fashion. Roadmapping may help laying out specific tasks and actions in time and how these can be combined to reach a certain target, *e.g.*, reaching sufficient direct air capture capacity deployed by the year 2050 to contribute to carbon dioxide removal while incurring acceptable energy consumption. Thus far, two such roadmaps have been published for DAC(CS): the mission innovation carbon removal technology roadmap^295^ and a roadmap published by Heriot Watt University and RMI (Rocky Mountain Institute),^17^ that was incorporated into RMI's applied innovation roadmap for CDR.^296^

Without going into the details of each of the roadmaps, their recommended activities fall into the following categories:

(1) Swift exploration of the materials space, physical properties/characteristics and benefits for all DAC technologies, using combinations of AI/ML-supported materials screening, materials synthesis and characterisation, and process modelling using the materials data obtained. This is a short-term action that should initially be completed by 2030, while a second or third round, *e.g.*, based on upscaling and demonstration findings can run into the 2030's.

(2) Materials stability and recyclability assurance for all DAC technologies, *via* prolonged laboratory testing, then field testing, to finalise before 2030.

(3) Detailed process, evaluation, and optimisation of the various DAC processes, aimed at understanding the performance potential for each DAC approach, including integrated materials – contactor – process optimisation. Also this activity needs finalising before 2030, with subsequent rounds to happen during the 2030's.

(4) Demonstration of all DAC pathways at pilot or demonstration scale, *i.e.*, TRL 6–8, because only through piloting an integrated prototype in its real operating environment can performance be confirmed, challenges and bottlenecks identified, and solutions for further scale up be generated. Or, allowing to discard certain approaches if insurmountable performance challenges are identified. TRL 6 piloting for each technology should be finalised by 2030, while higher TRL demonstration can also spill over for some technologies to 2030–2040.

(5) Development of specialised, ideally non-proprietary equipment for direct air capture processes and establishment plus scale up of equipment supply chains. This activity relates to transferring the burden of equipment development to companies who specialise in this, moving this burden from DAC companies to the experts such that more affordable equipment becomes widely available. This should also account for the supply of absorbents, adsorbents, and membranes. Such activities should also ideally be established by 2030 while spillover into the later years is expected.

(6) Studies investigating integration into and optimisation with existing and future energy and industrial sectors, to allow finding synergistic pathways to less energy and resource intensive DACCS. Also these studies ideally happen before 2030.

An important element highlighted by the HWU/RMI roadmap is that a good deal of the materials and process evaluations need to happen in a fully transparent manner by independent research organisations, to ensure the validity of materials and process performance claims put forward.^17^ This transparency and independence is critical to providing unbiased information the public domain, notably governments and investors, who have the burden of making well-informed decisions about CDR scaling.

**Fig. 17:**
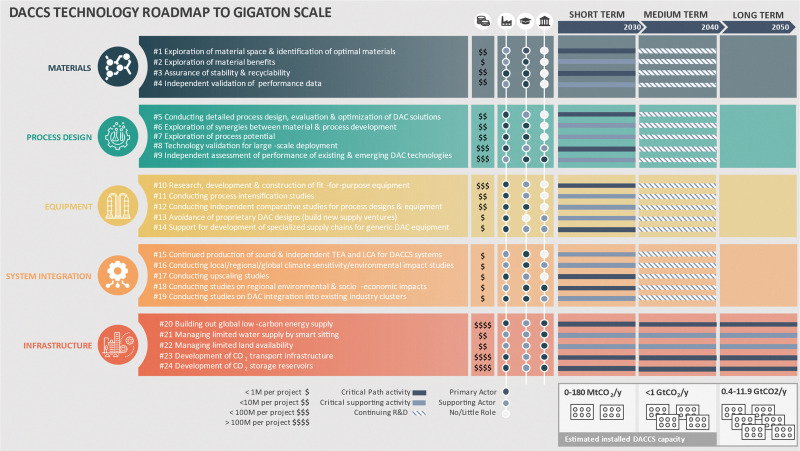
DACCS technology roadmap to gigaton scale as developed by Heriot Watt University and RMI.^17^ Used with permission (CC-BY).

Finally, both roadmaps stress the importance of removing non-technical barriers to improving performance and scaling DAC. Costs, environmental impacts, financing, market creation, supporting policies, and community engagement are all key for successful DAC scaling, and are discussed in the next sections. Fig. 17 summarises the HWU/RMI activities plus indicative timelines and activity costs into their roadmap.^17^

## 4. DAC technology performance assessment

### 4.1. Technoeconomic assessment

This section analyses a total of 27 peer-reviewed DAC techno-economic assessments (TEAs) to understand the associated costs of different technologies and identify gaps in the current knowledge base. The available data predominantly covers technologies using alkaline absorbents coupled to Ca-carbonation, and solid adsorbents. Specifically, 15 out of the 27 studies provided information on absorbents with Ca-looping, while 8 included data on solid sorbents. In contrast, only three studies addressed mineral looping or ambient weathering approaches, and just two studies examined alkaline absorbent systems with electrochemical regeneration. Comparisons of costs across different DAC technologies are relatively scarce, with only a few notable examples, including analyses by Fasihi *et al.*,^121^ Sievert *et al.*,^297^ Young *et al.*,^86^ Sabatino *et al.*,^83^ and Küng *et al.*^17^

#### 4.1.1. Levelised cost of (gross) CO_2_ captured

To facilitate cross-study comparisons, the elicited TEA data was harmonised using a set of standard assumptions. All cost figures were adjusted to present-day values using the chemical engineering plant cost index (CEPCI). For studies where the base year was not explicitly provided, the publication year was assumed as the base year. The target year for harmonization was July 2024, corresponding to the most recent available CEPCI data at the time of review.

Published TEA studies tend to report costs along two primary KPIs: levelized cost of (gross) CO_2_ captured (LCCC) and levelised cost of (net) CO_2_ removed (LCCR). The gross captured cost refers to the cost of capturing CO_2_ from ambient air, including both capital and operational expenditures of the process, but excluding emissions from energy or feedstock use, or other life cycle activities. In contrast, the net removed cost accounts for the life-cycle greenhouse gas (GHG) emissions associated with the capture process and includes the so-called carbon removal efficiency (CRE – units of CO_2_-eq net removed divided by units of CO_2_ gross captured) of the DAC system. To allow comparison, gross captured costs were used as the primary metric in this review, because net removed heavily depends on the energy source, and corresponding greenhouse gas intensity, used. In cases where only net removed costs were reported, an average CRE of 86% was applied to approximate gross captured costs, following Gutsch and Leker.^298^

**Fig. 18:**
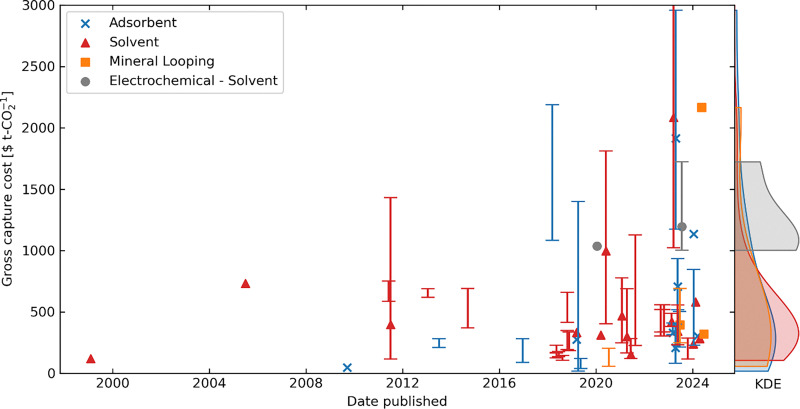
Comparison of DAC total levelised cost of CO_2_ captured (LCCC, gross), reported in the scientific literature over time. All values were converted to present-day terms using the chemical engineering plant cost index (CEPCI). Note that where only net removed cost was reported, the gross capture cost was calculated using an average carbon removal efficiency of 86%.^298^ Markers are reported point estimates, lines are reported ranges. The kernel density estimates on the right show the most reported LCCC values.

Like the energy requirements, our analysis shows that the cost of DAC in the public domain does not follow a clear decreasing trend over time. As Fig. 18 shows, the cost ranges published have increased over time, suggesting newer studies incorporate more cost dependent variables like type of regeneration energy and location. Both solvent and adsorbent technologies present a wide range of costs. Reported solvent system costs range from $105 to $3616 per tonne of CO_2_. The lower bound assumes the use of KOH, with minimal energy inputs and no associated oxygen costs.^105^ The upper bound reflects the use of MEA solvent coupled with low-grade heat stripping.^17^ Studies that have reported a range of costs (low, medium and high) for solvent technology yield averages of $300, $501 and $725 per tonne CO_2_ respectively.

Adsorbent-based DAC costs range between $18 and $2957 per ton of CO_2_. The lowest bound corresponds to a parametric study that assumes an optimized, energy-efficient scenario.^299^ The upper bound corresponds to a solid sorbent DAC plant in the US paired with nuclear power.^86^ Notably, certain cases exceed $3000 per ton, particularly when powered by grid electricity and depending on the plant's location (not shown in Fig. 18).^86^ For adsorbent technologies, the reported average costs for low, medium, and high estimates are $360, $614, and $938 per ton, respectively—only slightly higher than the corresponding averages for solvent-based technologies.

Electrochemical regeneration DAC emerges as the most expensive technology to date, with costs ranging from $1003 to $1722 per tonne of CO_2_, although data is limited to only two published studies by the same authors, and it has been suggested the operating points selected is vastly suboptimal, as the process was optimised assuming much higher than commercial bipolar membrane electrodialysis costs. In contrast, mineral looping offers a very broad cost range of $57 to $2165 per ton of CO_2_ compared to electrochemical DAC. The average low, medium, and high costs for mineral looping are $152, $446, and $959 per ton, respectively, with the low and medium estimates falling below those of solvent and adsorbent technologies. The variability in mineral looping costs can be attributed to differences in material and process assumptions. For instance, higher cost estimates reflect the use of CaO with novel plant components, such as tray movement robotics and an electric kiln.^297^ In comparison, lower-cost estimates use MgO spread on land coupled with a simpler, natural gas-fired calciner. The electric kiln is generally considered more complex and capital-intensive than the gas-fired version.

Importantly, our review also highlighted that many studies omit certain cost adders, *e.g.*, appropriate contingencies, owner's costs, and balance of plant items,^300^ leading to underrepresentation of total DAC costs. Equally, few studies include the full DAC value chain, including CO_2_ compression, transport, and storage (T&S). While T&S costs are often assumed to be consistent for processes producing highly concentrated CO_2_ streams (above 95% purity) (Küng *et al.*, 2023), these costs can vary significantly depending on the chosen transport and storage methods as well as the proximity and characteristics of storage sites. Comprehensive assessments that account for the entire value chain, including various transport and storage combinations, are essential for accurately evaluating the total costs and feasibility of DAC systems. Such holistic analyses will provide the insights needed to guide more effective deployment and technology development. Finally, most studies neglect to report whether their estimates are for current or future technologies and whether the estimates are aspirational or reflect real performance. Future research should, therefore, focus on systematically comparing different DAC technologies using standardised baseline assumptions and including all cost items encountered in real projects.

#### 4.1.2. Spatial DAC costs variations

**Fig. 19:**
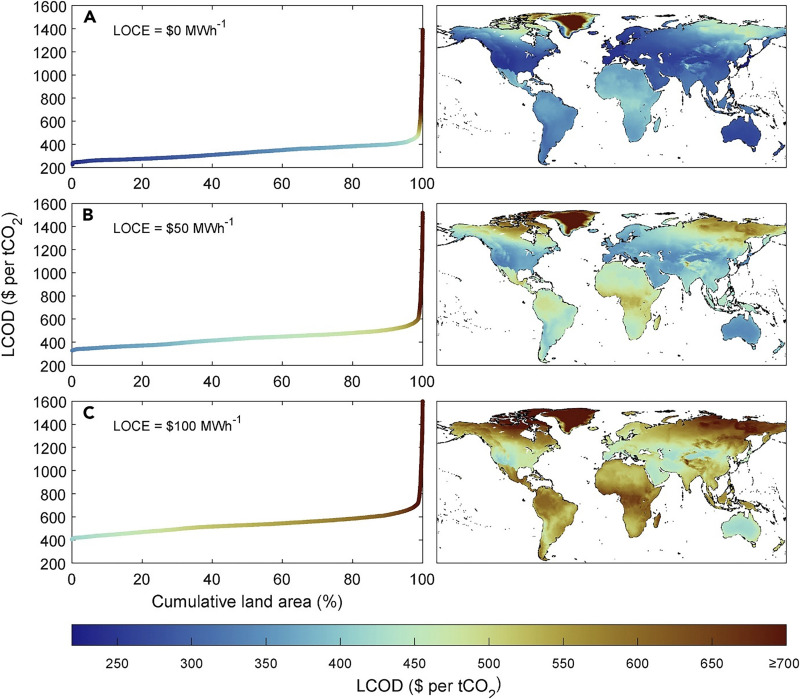
Global cost and supply curve for solid adsorbent-based vacuum temperature swing adsorption. The left figures show the global DAC supply curves at two different levelised costs of electricity (LCOEs) as a function of total land that can deliver DAC at the corresponding levelized cost of CO_2_ captured (LCCC). The colour of the data points on the supply curves matches their location on the corresponding map on the right. Darker blue indicates a lower LCCC, and darker brown indicates a higher LCCC. Reproduced from ref. 48 with permission from Elsevier, copyright 2022.

Data availability on DAC costs outside the United States remains scarce. Among the 27 studies reviewed, only two provide cost estimates for locations outside the U.S.,^48,86^ and of these, only one includes a direct comparison of technology costs. Fig. 19 presents the results of a global geospatial analysis conducted by Sendi *et al.*,^48^ which focuses on adsorption-based DAC using high-resolution temporal and spatial weather data. The analysis estimates the LCCC (defined as the levelised cost of DAC, LCOD, in their paper) for global land areas at two different electricity price levels. At a levelised cost of electricity of $50 per MWh, the LCCC ranges from $320 to $1518 per tCO_2_, while at $100 per MWh, it ranges from $403 to $1646 per tonne CO_2_.^48^ Meanwhile, Young *et al.*,^86^ showed that current and future DACCS costs are lowest for projects developed in China, potentially the US, owing to lower costs of labour respectively energy. The highest costs were anticipated for Brazil and Australia, suffering from high costs of construction and energy. This analysis highlights the substantial influence of geographic conditions on DAC costs, emphasizing the critical need for site-specific assessments to optimize global deployment strategies in future studies.

The significant variability in reported DAC costs underscores the importance of understanding and including both site-specific and technology-specific factors that drive economic performance. Therefore, it is imperative that cost analyses be expanded to a broader range of global locations, to capture opportunities for more economic DACCS deployment, *e.g.*, in regions where low carbon energy are abundant, and construction and resource costs are low.

#### 4.1.3. The impact of energy supply strategy

Where the previous sections discussed variations in DACCS costs for different technologies and locations, this section investigates the effect of energy supply. A small number of studies in our review sample discuss the effects of energy supply strategy, notably McQueen *et al.*,^112,301^ Sievert *et al.*,^297^ Sendi *et al.*,^302^ and Young *et al.*^86^

**Fig. 20:**
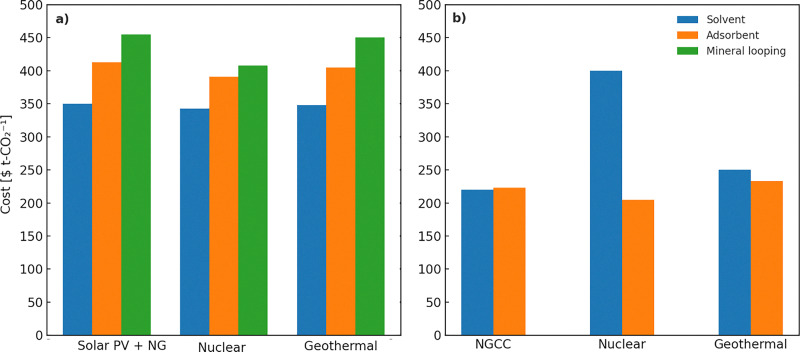
Comparison of the costs of direct air capture for different technology types and energy supply strategies. (a) Levelised cost of net CO_2_ removed for solvent, adsorbent, and mineral looping technologies when powered with solar PV with battery storage for electricity generation plus NG combustion for heat generation; nuclear heat and power; and geothermal heat and power. Costs are in 2022 USD.^297^ (b) Levelised cost of gross CO_2_ captured for solvent and adsorbent technologies when powered with natural gas combined cycle (NGCC) electricity; nuclear electricity; and geothermal electricity, assuming all heat is provided from electricity, *e.g.*, *via* an electric kiln for the solvent based system.^112,301^

McQueen *et al.*,^112,301^ Sievert *et al.*,^297^ compare the costs for various DAC technologies for a generic US location, presenting the levelised cost of net CO_2_ removed (LCCR), respectively gross CO_2_ captured (LCCC), for different DAC technologies and different energy supply strategies. Key insights from these studies are that using nuclear energy for energy provision may result in slightly lower costs, but the differences are small (Fig. 20). The results from the two studies follow roughly the same trend, while noting that the solvent-based DAC system powered by nuclear electricity is much more expensive in the study by McQueen, because they assumed the use of an electric kiln, which increases capital costs.

Young *et al.*,^86^ and Sendi *et al.*,^302^ took a more elaborate approach where they compared the LCCR and LCCC (respectively) for different energy supply strategies and different countries. Young concluded that location has more impact on the cost of DACCS than energy source for early projects as these tend to be driven by capital costs, directing towards countries where capital projects are lowest. Meanwhile, for future projects when capital costs have come down, lowest costs are established by using non-intermittent sources of low carbon energy, *e.g.*, hydropower, nuclear, or geothermal electricity. Sendi corroborates this by showing lowest costs across continents for solid sorbent DAC coupled to nuclear energy, followed by energy from natural gas combined cycles with CCS. Intermittent renewables consistently underperform in cost terms across all regions, even when paired with energy storage technologies. However, non-intermittent options such as nuclear and geothermal have limitations: nuclear power typically entails very long construction timelines, while geothermal potential is geographically constrained, which may restrict large-scale deployment of these energy sources. None of the reviewed studies expect very substantial cost decreases from using different energy supply strategies with costs remaining above $200 per tonne CO_2_ even when the cheapest energy strategies are applied. So, while energy supply can have small effects on individual project costs, the more substantial cost reductions may have to come from future technology improvements, discussed in the next section.

#### 4.1.4. Future cost estimates

To better understand the potential cost reduction in scaling up DAC technologies, some studies have differentiated between so-called first-of-a-kind (FOAK) and Nth-of-a-kind (NOAK) DAC plant costs. FOAK costs are typically higher due to substantial initial capital investments, limited design and operational experience, and the novelty of the technology. In contrast, NOAK costs benefit from economies of scale and the accumulation of technological learning (or experience), allowing plant designers and operators to reduce cost by incorporating their experience in plant design and operations.^300^

**Fig. 21:**
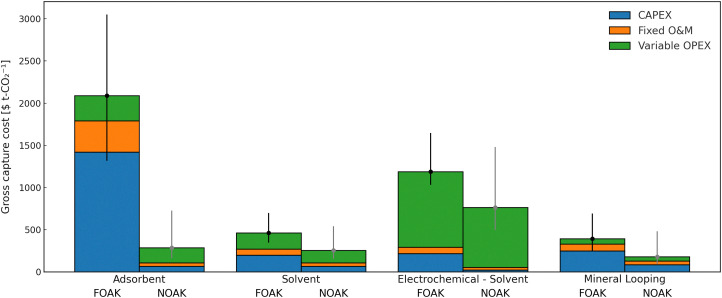
Capital and operating costs for first-of-a-kind (FOAK) and Nth-of-a-kind (NOAK) plants for adsorbent, solvent, electrochemical-solvent and mineral looping technologies. Data sourced from ref. 86 and adjusted to present day values. The left-hand stacked bars present the breakdown of the FOAK LCCC for each technology, paired with a heat pump for low-grade heat (where applicable) and electricity sourced from nuclear power for a generic US location. The right-hand stacked bar charts illustrate the LCCC at a scale of 1000 MtCO_2_ per year deployed.

Fig. 21 presents the capital expenditure (CAPEX) and operating expenditure (OPEX) for four distinct DAC technologies: solid sorbent, liquid absorbent, liquid absorbent with electrochemical regeneration, and mineral looping. The data, sourced from Young *et al.*,^86^ has been adjusted to reflect 2024 present-day values for gross levelized cost of CO_2_ capture. While Young provides cost estimates for multiple countries, including China, the UK, Germany, Brazil, Australia, and Oman, the data presented here focuses exclusively on the United States. The left-hand stacked bar charts depict the breakdown of the FOAK gross cost of CO_2_ capture for each technology, paired with a heat pump for low-grade heat (where applicable) and electricity sourced from nuclear power. The right-hand stacked bar charts illustrate the costs at a scale of 1000 MtCO_2_ per year deployed capacity. While other studies have provided CAPEX and OPEX breakdowns, direct comparisons between technologies are challenging due to differences in heat and electricity generation assumptions and therefore excluded.

Fig. 21 suggests that all technologies can expect substantial cost reductions transitioning from the FOAK to the NOAK scale, but the magnitude of these reductions varies. For instance, the average FOAK LCCC for solid sorbents is $2089 per tonne, primarily driven by CAPEX (68% of total costs). In contrast, the average NOAK cost for the same technology decreases to $288 per tonne, with a reduced CAPEX contribution of 24%. This cost reduction demonstrates the potential impact of technological learning as the technology matures.

For solvent-based DAC, the average FOAK cost is $462 per tonne, dominated by variable OPEX (42% of total costs). The average NOAK cost falls to $257 per tonne, where CAPEX and variable OPEX contribute more evenly to the total. In the case of electrochemical-solvent DAC, average FOAK costs are $1187 per tonne, with variable OPEX accounting for 75% of the total due to the very high energy consumption assumed in the original studies.^147,149^ Unlike other technologies, the CAPEX proportion is relatively low (18% of total costs), reflecting the energy-dominated nature of this method. Although average NOAK costs decline to $766 per tonne, the reduction is less pronounced, indicating that significant innovations in energy efficiency will be required for this technology to become cost competitive. Magnesium oxide looping with land spreading and natural gas-fired calcination exhibits the lowest LCCC among the assessed technologies, with FOAK and NOAK costs of $394 per tonne and $180 per tonne, respectively. The variable OPEX contribution is minimal in both cases, underscoring the cost advantages of this method, such as low material and energy costs and straightforward operations.

Notably, the above analysis relied on using learning rates for CAPEX and OPEX to arrive at NOAK costs. Different DAC technologies may exhibit distinct learning rates due to inherent technological differences. For example, liquid sorbent-based DAC systems tend to capitalize on economies of scale but are less likely to experience rapid improvements in design or manufacturing (Qiu *et al.*, 2022). Conversely, solid sorbent-based DAC technologies emphasize flexibility and modularity, which may facilitate faster iterative advancements and mass production. Several studies, including those by Sievert *et al.*,^297^ Hanna *et al.*,^303^ Qiu *et al.*,^304^ Young *et al.*,^86^ Fasihi *et al.*,^121^ and McQueen *et al.*,^21^ have examined and applied learning rates for different technologies. The latter two studies focus solely on incorporating learning or experience rates into capital expenditures (CAPEX), limiting their scope to material and energy consumption associated with capital investments. Given the nascent stage of DAC technologies, with limited deployment at scale and an insufficient number of Nth-of-a-kind (NOAK) facilities constructed, estimating operational expenditures (OPEX) learning rates remains a significant challenge, while it is anticipated that especially energy costs will come down.

**Fig. 22:**
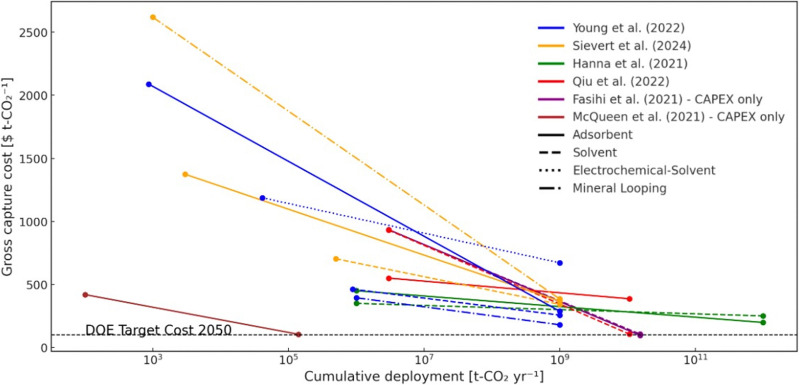
Published cost trajectories for four DAC technology types: solvent, adsorbent, electrochemical-solvent, and mineral looping. Recalculated to 2024 LCCC from ref. 21, 86, 121, 297, 303 and 304.

Fig. 22 shows the LCCC for FOAK and NOAK plants using different learning rates available in literature. The DOE DAC cost target for 2050 ($100 per tonne) (dotted line) is below most NOAK data points, except for two cases of solid sorbent technology and one case for solvent technology: most studies project DACCS to sit between 200 and 500$ per tCO_2_ at the gigatonne per annum deployed scale. This emphasises the likely unattainable cost reductions needed across all technologies to meet the US Department of Energy target, and it may be worthwhile revising this target upward. It also emphasises that direct air capture is not an economic alternative to emissions mitigation through, *e.g.*, renewable electricity production, point source CO_2_ capture, et cetera. The data underline that, indeed, it will act as a complement to other climate change mitigation options and can be expected to sit further down the marginal CO_2_ abatement curve.

### 4.2. Environmental performance

This section analyzes peer-reviewed life-cycle assessment (LCA) studies of direct air carbon capture and utilization and storage (DACCU or DACCS) to identify their expected environmental impacts and gaps in our current understanding. This work focuses on LCA studies investigating multiple environmental impact categories. We identified 15 studies addressing 4 distinct capture technologies to remove CO_2_ from the atmosphere: solvent-based high-temperature swing absorption (SV-DACCS), sorbent-based low-temperature swing adsorption (SB-DACCS), fast-swing sorbent-based low-temperature adsorption (fast-swing SB-DACCS), and sorbent-based humidity-swing adsorption (humidity-swing SB-DACCS). In the following, we introduce the scope of the reviewed LCA studies and summarize the reported energy demands for DAC operation (Table 8).

**Table 8:** Overview of studies presenting LCAs of direct air carbon capture and utilization or storage (DACCU or DACCS) systems across multiple environmental categories and their demands for electricity [kWh per tCO_2_ captured] and heat [MJ per tCO_2_ captured]

Study	DAC technology (scale)	Electricity demand [kWh per tCO_2_]	Heat demand [MJ per tCO_2_]
Van der Giesen *et al.*^313^	Humidity-swing SB-DAC (0.365 kt per year)	378	0
Zhang *et al.*^315^	SB-DAC (—)	370	6300
Wevers *et al.*^314^	SB-DAC (—)	250	6300
Rosental *et al.*^316^	SB-DAC (1.8 kt per year)	400–700	5760–7920
Mo *et al.*^317^	SB-DAC (—)	834	3200
Deutz and Bardow^96^	SB-DAC (4 kt per year)	700	11 900
	SB-DAC (100 kt per year)	500	5400
Terlouw *et al.*^307^	SB-DAC (100 kt per year)	500	5400
Madhu *et al.*^310^	SB-DAC (0.05 kt per year)	180	2600
	SV-DAC (1 Mt per year)	534	4050
Cooper *et al.*^312^	SB-DAC (981 kt per year)	323	18 152
Qiu *et al.*^304^	SB-DAC (100 kt per year)	500	5400
	SV-DAC (1 Mt per year)	345	6280
Cobo *et al.*^311^	SB-DAC (4 kt per year)	650	7200
	SV-DAC (1 Mt per year)	0	8809
Leonzio *et al.*^309^	SB-DAC (—)	299–1540	5040–4 680 000
Zahedi *et al.*^305^	SV-DAC (1 Mt per year)	351	6282
Prats-Salvado *et al.*^306^	SV-DAC (1 Mt per year)	0	8810
	Solar SV-DAC (1 Mt per year)	0	0
Ottenbros *et al.*^308^	Fast-swing SB-DAC (0.1 kt per year)	1500	0

Two of the studies focus on the environmental impacts of solvent-based direct air capture technologies: Zahedi *et al.*,^305^ simulate and compare the environmental impacts of SV-DACCS based on liquid absorption with amine solvents and strong sodium hydroxide solvents. The study indicates that amine-based carbon capture generally has fewer negative environmental effects than hydroxide-based capture but may result in higher energy consumption and fossil resource use. Prats-Salvado *et al.*,^306^ compare the environmental impacts of a SV-DAC process powered entirely by natural gas combustion with a solar-driven SV-DAC process. The study uses a cradle-to-gate system boundary, excluding post-capture processing of the CO_2_. As expected, the results show that the solar-powered SV-DAC approach offers higher carbon removal efficiency by avoiding the direct emissions associated with natural gas combustion and is more cost-effective than the natural gas-powered DAC system.

Multiple LCA studies consider sorbent-based DACCS supply chains: Deutz and Bardow^96^ assess the environmental impacts of low-temperature SB-DACCS real-world plants operated by Climeworks in Hinwil, Switzerland, and Hellisheidi, Iceland. This study compares the environmental impacts depending on the selected energy source and adsorbent. Both plants can already achieve negative emissions under current conditions for low-impact energy sources. The study also suggests that scaling up the DACCS plants to capture 1% of global annual CO_2_ emissions is feasible in terms of material and energy availability but requires an increase in amine production for adsorbents. Building on this work, Terlouw *et al.*,^307^ conduct comprehensive prospective LCAs of stand-alone and grid-connected low-temperature SB-DACCS configurations, investigating the effect of location and future transitions in the background sectors on the system's environmental impacts. Similarly to the results of Deutz and Bardow, all configurations can achieve negative GHG emissions, with the highest removal potential observed in regions with low-carbon electricity and waste heat. The study underscores the importance of including the whole supply chain of DACCS, including capture, compression, transportation, and permanent storage, when assessing its environmental impacts.

Ottenbros *et al.*,^308^ add to this body of research by assessing the prospective environmental impacts of a novel fast-swing SB-DACCS supply chain using solid-activated carbon sorbents. The study investigates both grid-connected and stand-alone configurations, finding that all configurations provide net benefits for climate and ecosystem health. The study aligns with previous works, suggesting that the environmental benefits of DACCS systems are enhanced when powered by lower-impact electricity sources. Lastly, Leonzio *et al.*,^309^ assess the environmental impact of low-temperature SB-DACCS with five different sorbents. The findings indicate that chemisorbents achieve net CO_2_ removal from the atmosphere with lower environmental impacts than physisorbents. The study also suggests future research should enhance sorbent properties to boost capture efficiency and reduce energy consumption, particularly for metal–organic frameworks.

Multiple studies compare the environmental performance of low-temperature SB-DACCS with high-temperature SV-DACCS supply chains. Madhu *et al.*,^310^ find that both SB-DACCS and SV-DACCS can achieve net carbon removal, with SB-DACCS generally outperforming SV-DACCS across several environmental categories. The study also performs an extensive sensitivity analysis on multiple assumptions, such as the sorbent and solvent recovery rates, concluding that the input requirements for chemical sorbents are not a limiting factor for the scale-up of DAC technologies, but can substantially affect their environmental impacts. Qiu *et al.*^304^ compare high-temperature SV-DACCS and low-temperature SB-DACCS technologies within a decarbonizing power system. Their findings indicate that SV-DACCS generally performs better environmentally than SB-DACCS across several impact metrics, which contrasts with the results of Madhu *et al.* Qiu *et al.* attribute this discrepancy to variations in technological assumptions and structural differences in the LCAs. Qiu *et al.* also highlight that both electricity sector decarbonization and advancements in DAC technological learning are crucial to preventing environmental problem-shifting and improving carbon removal efficiency. However, the findings also indicate that large-scale decarbonization also raises concerns about terrestrial ecotoxicity and metal depletion per tonne of CO_2_ captured. These negative impacts can be mitigated through improved efficiency in DAC materials and energy usage. In line with Terlouw *et al.*,^307^ the study also reveals that the environmental effects of DACCS vary regionally, emphasizing the need for strategic siting in energy system planning.

Studies also compare the environmental benefits and burdens of DACCS to other carbon dioxide removal (CDR) technologies: Cobo *et al.*^311^ assess the potential health and environmental benefits of both DACCS and bioenergy with carbon capture and storage (BECCS) as CDR technologies. For DACCS, the study performs a planetary boundary analysis for high- and low-temperature DACCS at a large scale and different energy mixes. The authors find that both CDR methods result in substantial health benefits. However, DACCS may further limit damage to biosphere integrity while limiting impacts on other earth systems. Similarly, Cooper *et al.*^312^ evaluate five CDR technologies across various environmental impact categories, including a high-temperature solvent-based DACCS system. Their analysis reveals that afforestation/reforestation and mangrove restoration are the environmentally most favorable on a per-ton CO_2_ basis. However, when evaluated on a per-ton CO_2_-per-year basis, enhanced weathering, BECCS, and DACCS show lower impacts. These findings highlight the importance of selecting CDR technologies based on specific removal goals, such as rapid CO_2_ reduction or consistent, long-term sequestration.

A few studies explore the potential of DACCS to compensate for emissions from point sources, such as power plants and industrial facilities. Van der Giesen *et al.*^313^ pioneer LCA studies of DACCS by assessing a humidity-swing DACCS process. The study compares the environmental impacts of reducing CO_2_ emissions from a coal-fired plant with either a point-source post-combustion carbon capture system or compensating *via* a distributed humidity-swing DACCS system. The findings indicate that humidity-swing DACCS can mitigate greenhouse gas (GHG) emissions effectively but may increase other environmental impacts. However, the use of photovoltaics to power the humidity-swing system and capturing all background GHG emissions can reduce these environmental effects. The study concludes that, if powered efficiently and in appropriate locations, humidity-swing DACCS can effectively complement point-source carbon capture, allowing for CO_2_ sequestration that is independent of the timing and location of emissions. Wevers *et al.*,^314^ explore pathways to net-zero-CO_2_ power systems, comparing systems that combine natural gas combustion with low-temperature SB-DACCS to those utilizing intermittent renewable electricity with seasonal storage through chemical energy. Their study finds that renewable systems with seasonal storage generally have lower climate change impacts than natural gas systems combined with low-temperature SB-DACCS. However, the study finds no single pathway excelling across all environmental impact categories.

A few studies focus specifically on low-temperature sorbent-based direct air capture and utilization (SB-DACCU) systems, where the captured CO_2_ is used for producing fuels and chemicals: Zhang *et al.*,^315^ assess the environmental performance of low-temperature sorbent-based DAC in the context of a power-to-gas study where the captured CO_2_ is used to produce synthetic natural gas. The findings suggest that power-to-gas can, depending on electricity and CO_2_ sources, reduce GHG emissions compared to conventional gas production, with power-to-hydrogen demonstrating greater emission reduction potential than power-to-methane. Power-to-hydrogen may also have lower environmental impacts than traditional hydrogen production, whereas power-to-methane generally has higher impacts than conventional natural gas. Rosental *et al.*,^316^ assess the environmental impacts of CO_2_ from point-source carbon capture and low-temperature SB-DACCS as a carbon source for producing organic chemicals. Similarly to Zhang *et al.*, the study reveals that carbon capture-based chemicals using offshore wind power can substantially reduce climate change impacts compared to their fossil counterparts. However, impacts may increase in other categories, such as eutrophication and ozone depletion. Mo *et al.*,^317^ explore an electrochemical process to synthesize ethanol by reducing CO_2_ from low-temperature SB-DAC. In line with Rosental *et al.*, the findings indicate that the electrochemical process can only reduce GHG emissions (and even reach carbon neutrality) when operated with low-carbon intensity electricity sources.

To summarize, both solvent-based and sorbent-based DACCS technologies have been shown to effectively remove more carbon dioxide from the atmosphere than they emit through their supply chains. However, the environmental outcomes of each technology vary across studies, with neither consistently outperforming the other. While both DACCS technologies offer environmental co-benefits, they can also lead to burden shifting and even increase climate impacts, depending on their implementation. A key factor driving both the carbon removal efficiency and environmental impacts of DACCS systems is their energy demand, particularly the source of this energy. This sensitivity makes the location of DACCS systems critical, as regional energy mixes strongly influence environmental performance. Table 8 summarizes the energy demands reported by each study for DAC operation. Moreover, a comprehensive environmental assessment requires a model of the entire DACCS supply chain, including capture, compression, transportation, and permanent storage, as emphasized by Terlouw *et al.*^307^ Still, this scope is not covered by all studies. Additionally, the reviewed studies differ in the functional unit, selected background processes and energy source, and assessed impact categories. These differences limit cross-study technology comparisons and thus prevent quantitative conclusions supported by several studies.

#### 4.2.1. Harmonisation framework for LCA study comparison

To enable cross-study comparisons, we harmonize methodological choices and data assumptions of LCA studies. Six studies were identified that offer sufficiently comprehensive life cycle inventories (LCIs) for the harmonisation, encompassing energy and sorbent demands for DACCS operations and material inventories for the capture infrastructure and post-capture CO_2_ processing. For the remaining 9 studies that do not provide comprehensive LCIs, we present energy consumption data for DAC in Table 8.

##### 4.2.1.1. System boundaries

The harmonization framework employed in this work aligns the background data, scope, and system boundaries across the selected LCA studies while maintaining study-dependent capture processes and technological assumptions. The system boundary employed in the harmonization captures the cradle-to-grave environmental impacts of carbon dioxide removal *via* DACCS. In this context, we define the functional unit of the system as ‘1 tonne of CO_2_ captured from air and permanently stored’, reflecting the objective of DACCS supply chains to lower atmospheric CO_2_ concentrations by removing and permanently storing CO_2_ from the air.

The harmonized system boundary includes the infrastructure and operational processes necessary for CO_2_ capture, conditioning, transport, and storage. Additionally, we consider the production of sorbents or solvents used in the capture process. For low-temperature SB-DACCS, we select amines on silica when the study provides LCIs for multiple sorbents (*e.g.*, ref. 96, or we use a sorbent proxy when the study does not offer an inventory for sorbents (*e.g.*, ref. 307). For fast-swing SB-DACCS, we employ potassium carbonate on activated carbon felt.^308^ For high-temperature SV-DACCS, potassium hydroxide (KOH) and limestone are utilized.^304,306,310^ The analysis also accounts for the end-of-life of infrastructure and chemicals used for CO_2_ capture, alongside potential leakages during CO_2_ transport. Although business travel was included in the study by Terlouw *et al.*,^307^ this analysis excludes travel due to its minimal impact on the overall system.

To encompass all components of the harmonized framework, we expand the system boundaries of the reviewed studies, supplementing any missing steps with the most detailed models identified in the literature:

• CO_2_ leakages during transportation: a leakage rate of 0.014 kg CO_2_ emitted per tonne CO_2_ stored and per km.^307^

• Construction, end-of-life, and operation for post-capture CO_2_ conditioning, transport, and storage: from Terlouw *et al.*^307^ for 100 kt per year plants, from Qiu *et al.*^304^ for 1 Mt per year plants.

• Construction and end-of-life of heat pumps: from Terlouw *et al.*,^307^ with study-dependent coefficients of performance.

The assumed distance for CO_2_ transportation varies across studies, ranging from zero kilometers (assumed in Deutz and Bardow^96^) to 1500 kilometers (assumed in Terlouw *et al.*^307^), with the first indicating co-location of the capture and storage facilities, and the latter indicating a potential cross-border CO_2_ transport. Within the harmonization framework, transportation distances are kept study-specific, while an average European GHG intensity of electricity is applied for post-capture CO_2_ conditioning, transportation, and geological storage. This approach ensures a consistent basis for comparison while accommodating variations in transportation assumptions across studies.

##### 4.2.1.2. Energy source

Several reviewed LCA studies explore integrating intermittent renewable energy sources with energy storage systems, emphasizing that these configurations may have significant environmental impacts. According to Young *et al.*,^86^ the optimal scenario for operating DACCS systems involves a nearly decarbonized grid. The study also highlights that pairing DACCS with intermittent renewable energy sources is unlikely to be cost-effective unless those sources exhibit high-capacity factors, as demonstrated by certain offshore wind installations. Therefore, our harmonization framework assumes electricity from the grid.

The heat source depends on the direct air capture technology investigated and is selected in the harmonization framework according to the following criteria:

High-temperature solvent-based direct air capture (SV-DACCS)

For high-temperature solvent-based DACCS (SV-DACCS) operating at temperatures up to 900 °C, heat is assumed to be supplied by natural gas combustion, in line with the literature. CO_2_ emissions resulting from the combustion can be captured to increase the net carbon removals of the DACCS supply chain. This approach is detailed in Qiu *et al.*^304^ The study assumes a full re-capture of CO_2_ emissions from natural gas combustion, with a factor of 0.0589 kg CO_2_ per MJ. Consequently, for every tonne of CO_2_ captured from the air, the carbon capture plant captures an additional 370 kg of CO_2_ from natural gas combustion. This analysis also incorporates the additional post-capture CO_2_ conditioning, transportation, and storage required for these captured combustion emissions. Here, we adopt the methodology of Qiu *et al.*,^304^ to directly co-capture CO_2_ emissions from natural gas combustion. By following this approach, we represent a best-case scenario, assuming that all CO_2_ emissions from natural gas combustion are captured by the plant, thereby minimizing the climate change impact of SV-DACCS technologies.

Low-temperature sorbent-based direct air capture (SB-DACCS)

For low-temperature sorbent-based DACCS (SB-DACCS), we assume a low-temperature heat supply at 100 °C *via* heat pumps in line with the literature.^96,307,318^ The coefficient of performance for these heat pumps remains study-dependent, ranging from 2.5 to 2.9.

While waste heat is often considered a lower-cost and lower-impact alternative,^96,307^ its scalability for sorbent-based DAC is limited by availability. Therefore, this harmonization prioritizes electrified heat pumps, which offer greater scalability and better integration with renewable energy sources.

##### 4.2.1.3. Background data and life cycle impact assessment

The harmonization framework is based on ecoinvent 3.9.1 (allocation, cut-off by classification)^319^ for background data and applies the environmental footprint 3.1 methodology for the impact assessment.^320^ The selection of location and source for background processes follows a hierarchical approach, prioritizing ecoinvent activities in the following order: “Market (RER – rest of European Region),” “Market (Europe without Switzerland),” “Market (Global),” “Production (RER),” “Production (Europe without Switzerland),” and “Production (Global).”

##### 4.2.1.4. Prospective assessment and scale-up

In this study, we utilize a linear scale-up approach to evaluate the environmental implications of storing 1 gigatonne of CO_2_ per year, as outlined in ref. 96. The resulting environmental impacts are normalized against planetary boundaries, which have been adapted to the environmental footprint reference package 2.0 metrics of each life cycle impact assessment (LCIA) category (LCIA-based planetary boundaries).^321^ LCIA-based planetary boundaries convert ecological thresholds into life cycle impact assessment metrics, enabling sustainability thresholds specific to each environmental impact category. For land use, we use the global normalization factor from environmental footprint 3.1,^320^ as the units of the corresponding LCIA-based planetary boundary differ from those of the environmental footprint methodology.

We consider the future transition of the electricity, transportation, steel, cement, and fuels sectors in prospective background databases *via* premise,^322^ with the integrated assessment model IMAGE.^323^ We follow the SSP2 pathway (middle of the road), which assumes population growth, quality of life, and technological progress will generally follow historical trends, and the RCP2.6 framework, which forecasts a rise in global mean temperatures of 1.7 to 1.8 °C by 2100.

##### 4.2.1.5. Carbon removal efficiency

To quantify the cradle-to-grave performance of direct air capture systems, we assess the carbon removal efficiency (*η*_CO_2__) using the following formula:
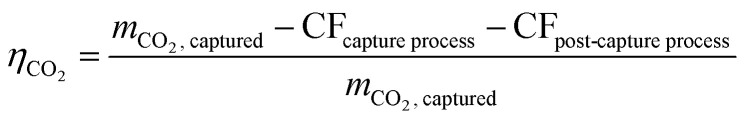


Here, *m*_CO_2_, captured_ is the mass of CO_2_ captured. CF_capture process_ is the life-cycle carbon footprint (CF) of capturing CO_2_. CF_post-capture process_ is the life-cycle carbon footprint related to conditioning, transport, and storage. The life-cycle carbon footprints consider all life-cycle stages from resource extraction, construction, and operation to end-of-life.

#### 4.2.2. Harmonised life cycle performance of DACCS systems

**Fig. 23:**
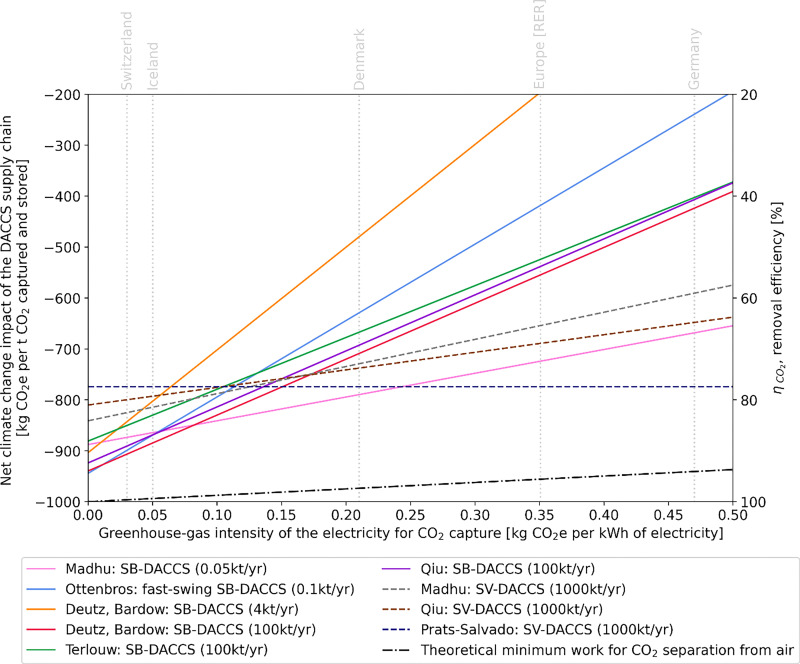
Climate change impact of DACCS as a function of electricity supply greenhouse gas intensity after harmonization of the investigated studies.^96,304,306–308,310^ Technologies include low-temperature adsorbent-based DACCS (SB-DACCS) and high-temperature solvent DACCS (SV-DACCS), with the co-capture of CO_2_ from natural gas combustion, as modelled in Qiu *et al.*^304^ The theoretical minimum work required to separate a stream of air with 400 ppm of CO_2_ into a high-purity CO_2_ stream and a residual air stream with 200 ppm of CO_2_ is quantified at 20 kJ mol^−1^ of CO_2_.^324^ This corresponds to 126 kWh per tonne of CO_2_. Country-specific GHG intensities for electricity are sourced from ecoinvent 3.9.1.^319^

Across harmonized studies, almost all DACCS technologies can contribute to CO_2_ removals for the analyzed range of grid GHG intensities (Fig. 23). However, the climate impact strongly depends on the grid GHG intensity for most systems, in line with previous literature (Section 4.2). The exception is the SV-DACCS configuration in Prats-Salvado *et al.*, which assumes that all energy demand is met through natural gas combustion.

The climate change impact of sorbent-based DACCS technologies is more sensitive to the grid GHG intensity than solvent-based DACCS due to a higher electricity demand for heat supply *via* heat pumps (Fig. 23). As a consequence, SV-DACCS outperforms SB-DACCS for high grid-intensities. By supplying SB-DACCS with waste heat instead of heat pumps, the climate change impacts reduce to levels comparable with those of SV-DACCS. At grid GHG intensities below 0.2 kg CO_2e_ per kWh, solvent and sorbent DACCS technologies exhibit similar climate change impacts. At this lower carbon intensity, additional environmental metrics become important for evaluating and differentiating the technologies. Worth noting is that all reported DACCS supply chains remain significantly above the theoretical minimum energy requirement for CO_2_ separation.

Sorbent-based DACCS supply chains capturing 100 ktCO_2_ per year, analyzed by Deutz and Bardow,^96^ Terlouw *et al.*,^307^ and Qiu *et al.*,^304^ rely on similar or identical inventories and assumptions and, therefore, perform similarly (Fig. 23). In contrast, smaller-scale systems, such as the 4 ktCO_2_ per year SB-DACCS supply chain studied by Deutz and Bardow^96^ and the 0.1 ktCO_2_ per year fast-swing SB-DACCS technology studied by Ottenbros *et al.*,^308^ show a higher electricity demand per tonne of CO_2_ captured. In contrast, the SB-DACCS system from Madhu *et al.*,^310^ indicates a lower electricity demand for direct air capture operations than other studies (Table 8).

##### 4.2.2.1. Contribution analysis of the climate change impact

**Fig. 24:**
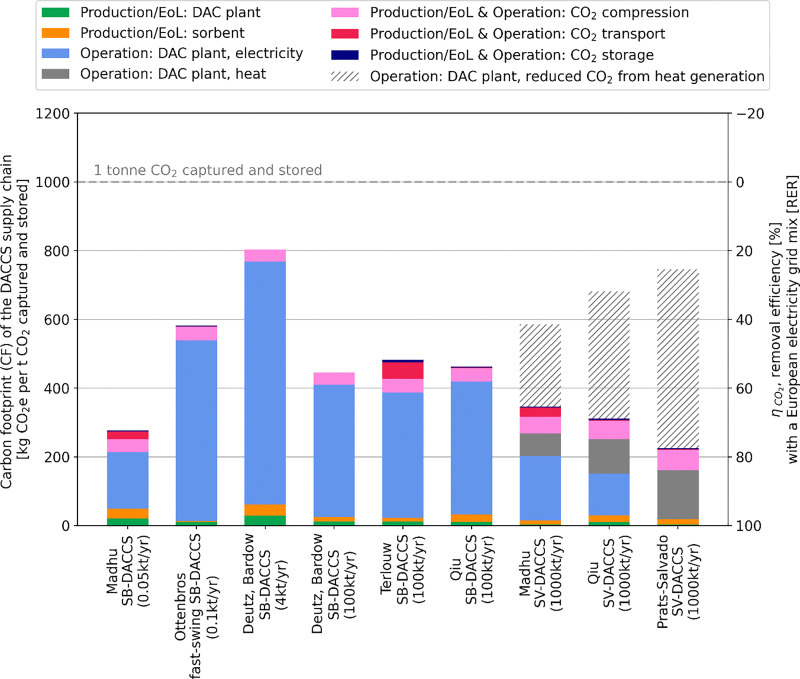
Breakdown of the climate change impact across different stages of the DACCS supply chain for CO_2_ capture and storage, based on harmonized data from the reviewed studies.^96,304,306–308,310^ A European electricity mix (RER) with a GHG intensity of 0.35 kg CO_2e_ per kWh, sourced from ecoinvent 3.9.1, is assumed for DACCS operation. Abbreviations: SB-DACCS refers to low-temperature adsorbent-based DACCS, while SV-DACCS refers to high-temperature solvent-based DACCS.

We examined the contributions of key life-cycle stages and steps of the DACCS supply chain to climate change impact after harmonization (Fig. 24). This assessment assumes a European electricity mix (see Fig. 23, Europe [RER]) with a GHG intensity of 0.35 kg CO_2e_ per kWh, as reported in ecoinvent 3.9.1.

For the European electricity mix, electricity supply to the capture plant operation dominates climate change impact for sorbent-based DACCS systems (SB-DACCS) with an average contribution of 80%. In contrast, for solvent-based DACCS (SV-DACCS), electricity contributes approximately 35% to the climate change impact when co-capturing emissions from natural gas combustion. Instead, high-temperature heat supply for the capture plant operation accounts for an additional 35% of the climate change impact of SV-DACCS systems. Without the co-capture of CO_2_ emissions from natural gas combustion (hatched area, Fig. 24), the climate change impact of SV-DACCS would increase by up to a factor of 3.

Another substantial contributor to climate change impacts is the post-capture CO_2_ conditioning, transportation, leakages, and geological storage, with an average contribution of 17% across the reviewed studies. The extent of this contribution varies based on the assumptions made in each LCA study. For instance, Terlouw *et al.*,^307^ adopt a conservative approach, modeling CO_2_ transportation over 1500 km with pipeline recompression and a leakage rate of 0.014 kg CO_2_ emitted per tonne of CO_2_ stored per kilometer. As a result, the climate change impact from post-capture CO_2_ conditioning, transport, leakages, and geological storage in their study amounts to 95 kg CO_2_ emitted per tonne of CO_2_ captured. In contrast, Deutz and Bardow^96^ assume capture plants to be positioned close to storage sites, thus eliminating the need for transportation and the associated leakage. This strategy reduces the contribution to 35 kg CO_2_ emitted per tonne of CO_2_ captured. Qiu *et al.*,^304^ Madhu *et al.*,^310^ and Ottenbros *et al.*,^308^ follow an intermediary approach with pipeline distances of 50 km, 300 km, and 300 km, respectively, resulting in varying impacts based on the specific circumstances of each study.

The production and end-of-life phases of sorbents and solvents contribute up to 10% and 7% of the climate change impact, respectively. However, the environmental impacts of these materials are highly uncertain: first, the consumption rates of sorbents and solvents per tonne of CO_2_ captured and the life cycle inventories of sorbents are uncertain^96^ and limited understanding of how a number of these materials perform under varying conditions, such as air humidity and temperature.^27,325^ Second, detailed inventories of industrial sorbent production are lacking, as sorbents for DAC applications are low-volume chemicals today.^96^ Lastly, the recycling rates of these materials remain uncertain due to lacking industrial realization. However, sorbent recycling rates and consumption may dramatically influence the performance of DACCS supply chains. For example, Madhu *et al.*,^310^ find an increase of 15% in climate change impact, when recovery rates for SV-DACCS systems are reduced from 99.9% to 99.0% or sorbent lifespan for SB-DACCS systems are reduced from 3 years to 0.5 years.

The construction and dismantling of infrastructure for CO_2_ capture have a relatively lower climate change impact, contributing, on average, 3% in studies assessing SB-DACCS and 2% in studies investigating SV-DACCS.

##### 4.2.2.2. Scale-up analysis and other environmental impacts

To evaluate the environmental impacts beyond climate change, we assume the large-scale deployment of DACCS aimed at achieving the net removal of 1 gigatonne of CO_2_.

**Fig. 25:**
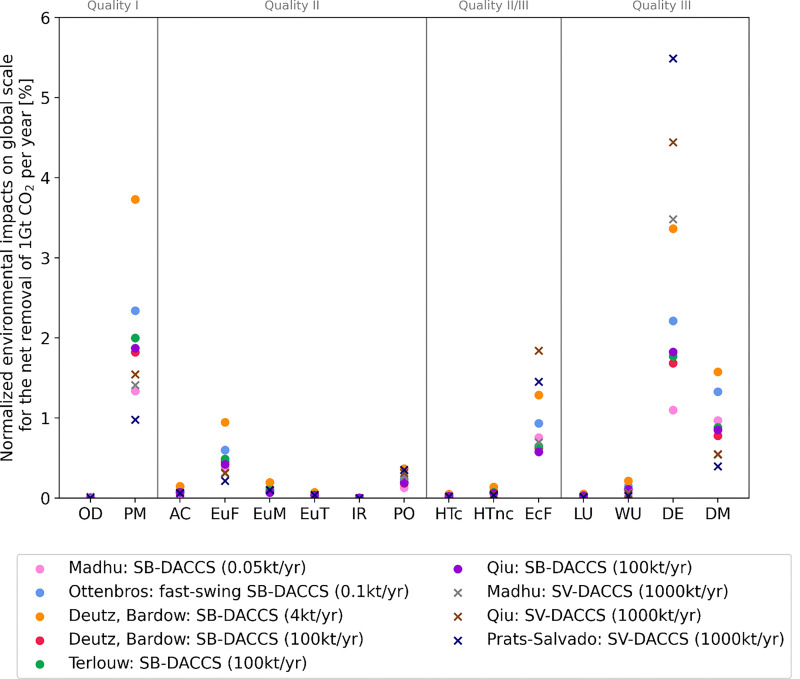
Environmental impacts normalized to a global scale for the net removal of 1 gigatonne CO_2_ per year. The environmental impacts are derived from a prospective life cycle assessment of harmonized DACCS technologies projected for the year 2050, under the SSP2-RCP2.6 climate mitigation scenario developed by IMAGE. The assessed environmental impacts include: ozone depletion (OD), particulate matter (PM), acidification (AC), eutrophication (freshwater, EuF; marine, EuM; and terrestrial, EuT), ionizing radiation (IR), photochemical oxidant formation (PO), human toxicity (carcinogenic, HTc; non-carcinogenic, HTnc), ecotoxicity (freshwater, EcF), land use (LU), water use (WU), depletion of non-renewable energy resources (DE), and depletion of material resources (metals/minerals, DM). The environmental impacts are presented in order of quality level, as defined by the European Commission's Joint Research Centre.^326^

Fig. 25 presents the environmental impacts for the net removal of 1 gigatonne of CO_2_ normalized by LCIA-based planetary boundaries. Neither SB-DACCS nor SV-DACCS consistently outperforms the other across all impact categories, and most impacts remain below 0.5% of the safe operating space (10 out of 15). However, the analysis reveals that net removal of 1 gigatonne of CO_2_ increases specific impact categories by a higher share, namely freshwater eutrophication (EuF) by 0.5–1%, freshwater ecotoxicity (EcF) by 0.5–2%, material resource depletion (DM) by 0.5–2%, particulate matter (PM) by 1–2.5%, and non-renewable energy resource depletion (DE) by 1–5.5%.

Within these categories, certain trends emerge: the non-renewable energy resource depletion is generally higher for SB-DACCS than for SV-DACCS due to the combustion of natural gas for high-temperature heat required by SV-DACCS. Reducing this reliance on natural gas combustion is essential to decrease non-renewable energy resource depletion for SV-DACCS. Additionally, the SV-DACCS non-renewable energy resource depletion findings emphasize that DACCS alone cannot substitute for large-scale mitigation efforts or the widespread adoption of renewable energy solutions. The environmental impacts on the eutrophication of freshwater, particulate matter formation, and depletion of material resources of SB-DACCS are generally higher than for SV-DACCS. This difference is driven largely by SB-DACCS's greater electricity demands, especially when low-temperature heat is supplemented with heat pumps. The higher depletion of material resources in SB-DACCS also reflects the material demands for plant construction and corresponding end-of-life. These findings align with those in Qiu *et al.*,^304^ who also compared the environmental performance of SV- and SB-DACCS technologies. Finally, the environmental impacts on freshwater ecotoxicity are typically higher for SV-DACCS, primarily due to the production and disposal of potassium hydroxide solvent.

In conclusion, our findings indicate that large-scale deployment of DACCS is accompanied by mostly mild increases in most environmental impact categories, in line with previous literature.^96,311^ However, certain impact categories see a more substantial increase: freshwater eutrophication, freshwater ecotoxicity, material resource depletion, particulate matter, and non-renewable energy resource depletion. These results may be influenced by the limited availability of life cycle inventory (LCI) data and the reliance on similar, if not identical, estimates and proxies across studies, contributing to the uncertainty surrounding the environmental impacts of DACCS.

#### 4.2.3. Outlook

LCAs accompany the development DACCS and enhance the understanding of their environmental impacts, as demonstrated by the studies reviewed in this work. However, several gaps persist in the assessment of DACCS supply chains and their environmental implications:

• The limited availability of life cycle inventory (LCI) data and reliance on similar or identical estimates across studies contribute to uncertainty regarding the environmental impacts of DACCS. Future studies should facilitate primary data sharing and incorporate more real-world data across a diverse range of DACCS technologies to enhance the robustness and applicability of DAC assessments, as outlined in Section 3.10.

• The current life-cycle inventories are mainly based on the required energy demands. More data on all other components and their environmental fate are highly needed. Further research is thus essential to investigate the potential degradation of sorbents, the subsequent release of chemicals into the atmosphere during DAC operations, and their local environmental and health impacts, particularly at larger scales.

• Future LCAs should encompass all supply chain components, including compression, transportation, CO_2_ storage, and recycling effects. For solvent-based DACCS, special attention should be given to the treatment and storage of additional CO_2_ co-captured from natural gas combustion, where applicable. Moreover, it is essential to incorporate real-world data and ensure diverse representation across multiple DAC technologies in order to increase confidence in the LCA results.

• Large-scale LCAs, ideally extending beyond linear-scale-up assumptions, are necessary to better understand the broader environmental implications of DAC implementation. This approach will allow more accurate evaluations of the potential environmental impacts of scaling DACCS technologies.

• Additionally, the location-specific performance of DACCS technologies is important. Such analyses should evaluate inlet air conditions such as humidity, temperature, and wind speed, which can substantially influence the effectiveness and environmental footprint of DACCS systems.^27,28,325^ Incorporating these factors into LCA analyses will provide a more comprehensive understanding of DACCS performance across different contexts, enhance the accuracy of environmental impact assessments, and guide technology development and deployment.

## 5. Socioeconomic considerations

### 5.1. Socio-political considerations and governance

The existing governance literature in this field contributes to one of the following aspects or a combination thereof. It (i) offers insights into governance challenges related to large-scale deployment of DACCS, (ii) assesses existing policies or policy lacunas (*e.g.*, ref. 327) as well historical or technology analogues, and (iii) normatively lays out policy proposals and principles for how DACCS deployment should be governed.

Previous studies have identified a series of challenges that governance of DACCS deployment needs to attend to, including: challenges related to business models, financial costs^21,50,86,318,328^ and how to distribute them;^329,330^ challenges related to high energy demand of DACCS and the necessary integration in renewable energy systems,^331^ co-location and transport requirements,^332^ including connections to oil and gas infrastructures,^333^ and debates around how to leverage these for DACCS upscaling;^334^ and challenges related to institutional capacities for mitigation measures^335^ as well as a number of socio-political challenges, including justice and equity, and public acceptance (see Sections 5.2 and 5.3).

In addition to policy considerations for CDR methods that also apply to DACCS, DACCS specific governance principles have been proposed. Based on expert interviews, Sovacool *et al.*,^336^ suggest DAC policy should follow principles that “ensure negative emissions”, “prioritize long-term carbon storage”, “appreciate scale and incentivize experimentation”, “co-develop with capture, transport and storage”, “phase in a carbon price”, “couple with renewables”, “harness hub deployment”, “maintain separate targets”, “embrace certification and compliance” and “recognize social acceptance”. Honegger *et al.*^332^ offer DACCS specific reflections on how to adapt and operationalize the governance principles that they put forward for CDR methods more generally. In order to ensure their principle of “environmental integrity”, they suggest DACCS should be deployed on sites that have a “structural surplus of zero-carbon energy”. Their principle “international cooperation and supports” means in the case of DACCS, on the one hand, that efficiency gains might be possible if capture and storage take place in separate jurisdictions and, on the other hand, that technology transfer and capacity development need to be fostered.

In addition to broad governance principles, specific policy instruments that are discussed in the wider CDR literature are also relevant for DACCS, most notably these are focused on creating economic incentives and market systems. These include debates round incentivizing CDR *via* carbon taxes and emissions trading schemes, crediting negative emissions. Rickels *et al.*,^337^ for example, suggest that in early phases supply of carbon removal credits should be organized by a carbon central bank, thus avoiding direct exchange between carbon removal companies and emitters. Bednar *et al.*^338^ outline a system where carbon tax revenues are partially invested in financing carbon removal at a later point in time, thus fostering technology deployment. Jenkins *et al.*^339^ discuss industrial “carbon takeback obligations” to remove emitted carbon, indirectly fostering investments into technology development and efficiency increases, ultimately contributing to reducing costs of carbon removal.

Literature touching on governance questions at the scale of project implementation at local sites includes calls for public and community engagement and suggestions around community ownership and benefit agreements.^340^ DACCS governance and deployment may prioritize bottom-up decision-making and community ownership and control, a soft path, or instead engender top-down centralized control from industry at the expense of communities.^341^

In the following we shed light on two key governance challenges related to DACCS and review propositions of how policymaking should attend to them: (i) equity and justice and (ii) public perceptions.

### 5.2. Equity and justice dimensions of DAC

One salient element of social dimensions of DAC uptake, or opposition, connects to real or perceived issues of equity and justice. These connections to equity and justice may not appear at first as obvious but are nevertheless omnipresent in various dimensions. The literature generally differentiates between distribution equity issues such as the creation of new adverse outcomes, procedural equity issues including planning and community engagement as well as ownership patterns, spatial equity issues such as air pollution benefits or disparities in adoption, and lastly intergenerational equity issues such as moral hazard and risks for future generations.

Distributive equity issues are a recurring theme in the literature and include at a fundamental level uncertainty over future DAC costs^328^ and thus whether they will be an economic burden, or benefit, to adopting communities, cities, and countries. Whether the distribution of costs and benefits associated with DAC uptake are a net positive or negative will depend upon a range of factors including its economic viability, technical performance, rates of learning and knowledge exchange, and government support and targets (among other factors). Young *et al.*^86^ caution that the cost of DAC will need to fall significantly to benefit adopters and even then their assessment warned that coupling to variable renewable energy only is unfavourable from a cost profile, and that investment grants are best suited to support small rather than large projects—two salient equity concerns. DAC systems can also introduce new economic, social, and environmental risks. Sovacool *et al.*^342^ examined justice and equity risks from DAC at a whole-systems level, from mining and manufacturing to waste, and charted a host of disadvantages shown in Table 9. Sovacool *et al.*^343^ interviewed experts about future DAC deployment and noted equity disadvantages over cost (the necessity for trillions of dollars of new investment that could have otherwise been directed at energy efficiency or renewable energy), environmental impacts (such energy intensity, water use and land use and consequent biodiversity loss), and concerns over liability (durability and performance of carbon storage as well as associated air pollution from diesel trucks and construction).

**Table 9:** Negative equity and justice impacts of direct air capture from a whole systems perspective. Modified from ref. 342

Resource extraction, chemicals, and fertilizers	Manufacturing, labor and ownership	Transportation, construction and land grabbing	Policymaking and planning	Deployment, operation and use	Disposal and waste
Affiliated mining and material needs (concrete), chemical pollution from solvents	Resource-curse risks for workers and communities, creation of sacrifice zones near deployment, strengthening of fossil-fuel incumbents when coupled to enhanced oil recovery	Creation of pipelines and storage reservoirs, competition with other land-uses, displacement of vulnerable groups, increases in fossil fuel consumption	Irresponsible distribution of risks between Global North and Global South, a “Pandora's Box” of liability concerns over stored carbon, loss of freedom for future generations	Immediate increases in energy consumption and affiliated air pollution or greenhouse gas emissions (if fossil-fueled)	Accidents at storage sites including suffocation of host communities, impermanence of long-term storage, earthquakes, energy penalties and increased resource waste

Distributional equity issues arise alongside procedural equity concerns about planning and community engagement as well as ownership. On one level, the inclusion of DAC into climate action plans can promote “restorative justice” and “reparative justice” as it enables the fossil fuel industry to reduce their historic harm and provide new benefits for communities.^344^ However, Batres *et al.*^345^ challenge that communities may not have sufficient resources or understanding of DAC to make wise investments, and that community involvement may be insufficient to ensure equity objectives are accomplished. Moreover,^346^ add that DAC development could benefit developers, investors, and firms rather than communities, leading to unequal power relations that distort the distribution of benefits. Lastly, Low *et al.*^341^ identify how some pathways of DAC deployment can promote community empowerment and equity, such as when done in smaller scales, coupled to renewable energy, and owned by a diversity of communities or cooperatives. Nevertheless, the same authors also identified a contrary pathway of an industrial approach to DAC that is more centralized, coupled to fossil fuels or oil recovery, owned by corporations, with benefits consolidated for incumbents that can actually harm communities.

**Fig. 26:**
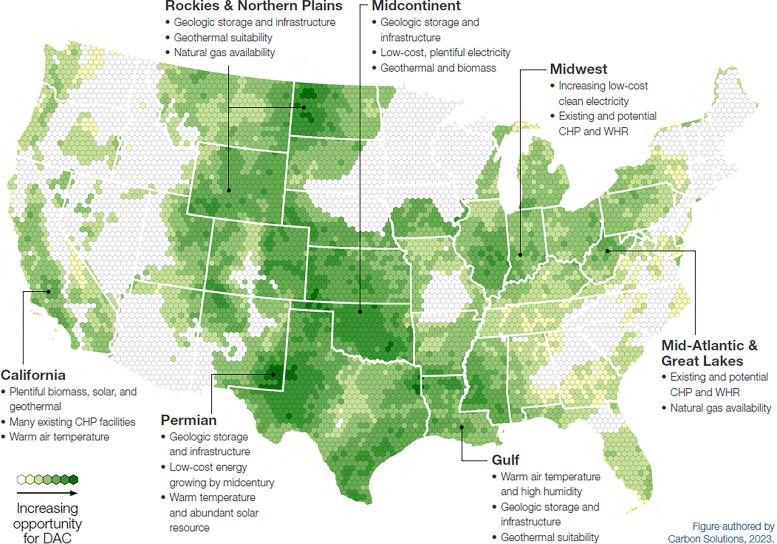
The spatial complexity of the suitability and scalability of DAC deployment in the United States. Reproduced from ref. 347 with permission from Great Plains Institute, copyright 2022.

Spatial equity issues with DAC concern patterns of future land use as well as air pollution benefits and disparities. As Fig. 26 indicates, in regions such as the United States, the suitability and optimality of DAC is heavily shaped by geographic factors such as geological storage, infrastructure, proximity to industry, and energy resource portfolios. Adoption is therefore mediated by, and can generate substantial disparities, in spatial justice.

**Fig. 27:**
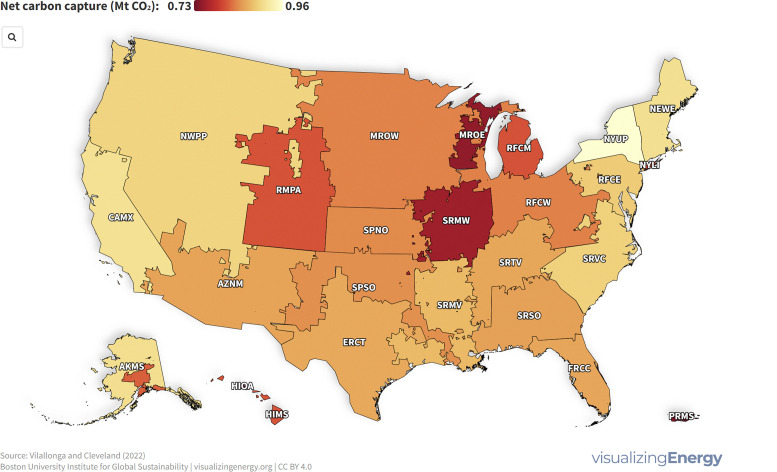
The spatially uneven climate benefits of DAC adoption in the United States. Reproduced from ref. 352 with permission from Boston University, copyright 2023.

Unfortunately, multiple studies have criticized future DAC deployment on the grounds that it could worsen air quality and air pollution distribution both in absolute terms – fossil-fueled DAC would contribute to greater emissions of particle pollution, ozone, and acid rain (Section 4.2.2.2 and Fig. 25) and relative terms – deployment primarily in wealthier states or urban areas could benefit least communities of color (*e.g.* ref. 348). This pattern of air pollution disparities in climate policy and implementation have already been confirmed in a multitude of previous studies (Fig. 27).^349–351^

Another equity concern in the literature is that of intergenerational complexities and future generations. Although DAC has much promise, its widespread deployment is still many years to decades away. One expert elicitation survey anticipated commercialization above 20% market share after 2050.^353^ Other projections based on historical analogues also put widespread use past midcentury.^354^ This creates a temporal equity issue given DAC essentially separates in space and time emissions and mitigation efforts, and it can create a moral hazard against action now in lieu of future, but uncertain, innovation.^355^ The feasibility of DAC thus becomes about choices concerning “the inter-generational equity” of climate pathways.^356^

In sum, DAC deployment is entwined with distributive, spatial, procedural, and temporal equity concerns. These are all attenuated, or dependent, on scale, meaning they may remain marginal if DAC is not deployed at scale, but become sober and widespread if DAC is scaled up. DAC governance and deployment may prioritize bottom-up decision-making and community ownership and control, a soft path, or instead engender top-down centralized control from industry at the expense of communities. DAC projects definitely can put justice, and equity concerns front and center, but whether they will do so effectively remains to be seen.

### 5.3. Public perceptions of DACCS

Public and stakeholder perceptions of DACCS represent another ripe area for investigation. Although a prospective role for DACCS within the transition outlined by the Paris Agreement is discussed in policy and expert circles, the general public remain broadly unfamiliar with this technology.^340,357–363^ Given the kind of large-scale, potentially urgent deployment envisioned, it is crucial that the public be on board, lest such an agenda prompts the blowback and controversy which has bedeviled, notably, genetic modification in the food sector or nuclear power. As a result, the question of social acceptance or having a social license to operate, *e.g.*, in fence-line communities where DACCS would operate, is resonant.^336,357,364–366^ At a minimum, such issues help to establish the geographical bounds in which DACCS might thrive – as a counterpoint, the severe limitations on the storage of carbon dioxide in Germany and Austria offer a worst-case scenario.^345,367^

Reflecting the technology itself, the literature on public perceptions of DACCS remains nascent – amounting to 26 articles, the first published in 2013 (see Table 10). These studies vary in terms of methodological approach and, to an extent, geographic focus. There is a notable uptick in public-perceptions research on DACCS, with two-thirds of studies published in the last four years, along with the first DACCS-exclusive studies^340,368,369^ being published in the last years. In terms of methodological focus, the majority^17^ employed a quantitative approach (such as representative surveys), a quarter^9^ a qualitative approach (*e.g.*, focus groups, deliberative workshops, interviews), and slightly less^7^ a mixed-methods approach. There is however a significant over-representation of Western developed countries, notably the United Kingdom and United States. These two countries featured, as the sole focus, alongside each other or, occasionally, with other Anglosphere countries (*i.e.*, Australia, New Zealand, Canada), in nearly two-quarters^21^ of the studies. As such, the literature on public perceptions gives insights on a specific type of publics – the only countries sampled in relation to DACCS not from this sphere are Germany (once), Norway (once), and Switzerland (twice). The only exceptions include one mixed-methods analysis of social media data (Twitter) – this does focus on English-language tweets.^370^ Recently, there have also been several studies emerging from a global-level set of nationally representative surveys in 30 countries and 19 languages,^363,371^ which were also accompanied by an overlapping 44 focus groups (one urban, one rural) in 22 countries.^362,366,372^ Until these studies, no publics from Asia, Africa, or Latin America – thus, much of the Global South – had been represented.

**Table 10:** Public-perceptions studies identified by literature review

Authors	Year	Focus country	Methods
Corner *et al.*^373^	2013	United Kingdom	Deliberative workshops (*N* = 44)
Wright *et al.*^374^	2014	New Zealand, Australia	Mixed methods – demi-structured interviews (New Zealand; *N* = 30)), brand image analysis (*N* = 2028)
Corner and Pidgeon^375^	2015	United Kingdom	Survey: 2 × 1 between-subjects design (*N* = 412)
Bellamy *et al.*^376^	2016	United Kingdom	Deliberative workshops (*N* = 13)
Gregory *et al.*^377^	2016	United States	Survey (decision pathway) (*N* = 800)
McLaren *et al.*^378^	2016	United Kingdom	Deliberative workshops (*N* = 44)
Bellamy *et al.*^379^	2017	United Kingdom	“Experimental” deliberative workshops: 3 × 1 between-subjects design (*N* = 21)
Campbell-Arvai *et al.*^380^	2017	United States	Survey: 5 × 1 between-subjects design (*N* = 984)
Buck^365^	2018	United States	Semi-structured interviews and site visits (*N* = 32)
Wolske *et al.*^381^	2019	United States	Survey: 3 × 2 between-subjects design (*N* = 980)
Carlisle *et al.*^358^	2020	New Zealand, Australia, United States, United Kingdom	Mixed methods – semi-structured interviews (New Zealand; *N* = 15), brand image analysis and survey (*N* = 2978)
Cox *et al.*^357^	2020a	United Kingdom, United States	Mixed methods – deliberative workshops (*N* = 8), survey (*N* = 2026)
Cox *et al.*^382^	2020b	United Kingdom, United States	Semi-structured (informed stakeholder) interviews (*N* = 17)
Jobin and Siegrist^360^	2020	Switzerland	Survey: 10 × 1 between-subjects design (*N* = 1575)
Shrum *et al.*^383^	2020	United States	Survey (exploratory) (*N* = 113)
Sweet *et al.*^384^	2021	United States	Survey: 3 × 1 between-subjects design (*N* = 1222; only those who believed at least “somewhat” in climate change)
Wenger *et al.*^361^	2021	Switzerland	Survey: 5 × 3 between-subjects design (*N* = 693)
Bellamy^385^	2022	United Kingdom	Survey (*N* = 2111)
Carlisle *et al.*^359^	2022	United Kingdom	Survey: 3 × 1 between-subjects design (*N* = 1558)
Merk *et al.*^367^	2023	Germany	Multifactorial vignette experiment (survey) (*N* = 1689)
Nawaz *et al.*^386^	2023	United States, Canada	Survey (*N* = 2120)
Müller-Hansen *et al.*^370^	2023	N/A	Mixed methods – social media analysis (*N* = 1 452 184 tweets from *N* = 314 484 users)
Satterfield *et al.*^368^	2023	United States, Canada	Survey (*N* = 2120)
Baum *et al.*^363^	2024	Brazil, Chile, India, Indonesia, South Africa, Kenya, Saudi Arabia, Nigeria, Dominican Republic, China, Singapore, United States, United Kingdom, Canada, Australia, Japan, Austria, Germany, France, Sweden, Poland, Switzerland, Greece, Italy, Netherlands, Norway, Spain, Denmark, Turkey, Estonia	Surveys (*N* = 30 284; at least 1000 in each country)
Cox *et al.*^387^	2024	United Kingdom	Survey: 2 × 2 + 1 between-subjects design (*N* = 1978)
Fritz *et al.*^366^	2024a	Australia, Austria, Germany, Switzerland, Poland, Spain, Italy, Norway, Sweden, United Kingdom, United States; South Africa, India, China, Indonesia, Chile, Brazil, Turkey, Saudi Arabia; Kenya, Nigeria, Dominican Republic	Focus groups (*N* = 323, in 44 focus groups (one urban, one rural) in each country)
Fritz *et al.*^372^	2024b	Australia, Austria, Germany, Switzerland, Poland, Spain, Italy, Norway, Sweden, United Kingdom, United States; South Africa, India, China, Indonesia, Chile, Brazil, Turkey, Saudi Arabia; Kenya, Nigeria, Dominican Republic	Mixed methods – focus groups (*N* = 323); survey (*N* = 22 222)
Gaspers *et al.*^388^	2024	Norway	Group model building workshops with stakeholders (*N* = 25, in three workshops)
Low *et al.*^362^	2024	Australia, Austria, Germany, Switzerland, Poland, Spain, Italy, Norway, Sweden, United Kingdom, United States; South Africa, India, China, Indonesia, Chile, Brazil, Turkey, Saudi Arabia; Kenya, Nigeria, Dominican Republic	Focus groups (*N* = 323, in 44 focus groups (one urban, one rural) in each country)
Scott-Buechler *et al.*^340^	2024	United States	Mixed methods – focus groups (*N* = 73); survey (with conjoint analysis) (*N* = 1195)
Sloot and Bostrom^369^	2024	United States	Survey (*N* = 2891)
Sovacool *et al.*^371^	2024	Brazil, Chile, India, Indonesia, South Africa, Kenya, Saudi Arabia, Nigeria, Dominican Republic, China, Singapore, United States, United Kingdom, Canada, Australia, Japan, Austria, Germany, France, Sweden, Poland, Switzerland, Greece, Italy, Netherlands, Norway, Spain, Denmark, Turkey, Estonia	Surveys (*N* = 30 284; at least 1000 in each country)
Yang *et al.*^389^	2024	N/A	Mixed methods with participants in European CDR market (project developers and financiers) – survey (*N* = 47); in-depth interviews (*N* = 27)

Caveats aside, the literature offers numerous insights into the contours of potential social and public acceptance, how these vary for respective publics, and prospective selling points or barriers of DACCS. First, there is evidence that DACCS tends to be viewed positively and does not yet provoke significant controversy. This is generally true both in the anglosphere^340,357–359,376,381,384,385,387^ and other Western countries.^360,361,367^ In fact, Baum *et al.*^363^ and Fritz *et al.*^372^ identify significantly stronger support for DACCS across the Global South – a situation holding for almost all climate-intervention technologies considered. Nawaz *et al.*^386^ do identify lower comfort and support with offshore forms of DACCS in the Pacific Northwest of Canada and the United States, with Satterfield *et al.*^368^ identifying specific concerns about leakage, storage, and the use of below-sea components driving rejection of such a system. Of course, it must be acknowledged that the public perceptions registered by surveys, focus groups, and deliberative workshops are chiefly founded on hypothetical experiences with DACCS, given the current development of this technology – what have been termed “pseudo-opinions”.^390^

Second, while there are indications of prospective support for DACCS, this tends to lag behind other forms of carbon dioxide removal, notably, afforestation.^360,361,363,366,367,381,384,385^ In their survey of Swiss citizens, Wenger *et al.*^361^ found that the perceived risks of DACCS were perceived to the highest, and the perceived benefits the lowest, among five CDR technologies.

We can ascertain an increasingly clear understanding of why DACCS is less preferred from the available literature. Three key concerns tend to emerge, the most frequent of which is its perceived lack of naturalness or that it tampers with nature.^360,361,373,381,384^ Similarly, in their analysis of the “associations” linked to DACCS, *inter alia*, Carlisle *et al.*^358,359^ found DACCS tends to be viewed as “artificial”, “risky”, having “unknown effects” – DACCS was also deemed less “cost effective” than other approaches (see also Bellamy^385^). As a result, those with stronger resistance towards interfering with nature tend to be more opposed to DACCS.^373,381^ Of note, the issue of tampering with nature is a point of overlap between experts from academia and industry^391^ and the general public. At the same time, DACCS is positively perceived for its lower land requirements and, prospectively, reduced threats to biodiversity *vis-à-vis* other CDR approaches.^357,388^ Attempts to frame DACCS as more natural, *e.g.*, as working like “artificial trees”^375^ or “putting [carbon dioxide] back in the ground”,^376^ also seem to hold some potential to increase support.

The second key concern involves the safety and reliability of geological storage of captured carbon, with individuals explicitly focusing on the potential for leakage.^340,357,362–364,376,378,387^ Such concerns were notably heightened in marine environments,^357,368^ while Low *et al.*^362^ highlights their prevalence in both global North and global South countries, but more overwhelmingly in the global North. Storage considerations also often dovetailed with questions about air pollution,^340,357^ with focus-group participants in the United States asking if DACCS could be used to improve air pollution.^340^ This perception that DACCS has co-benefits for improving air quality also emerged in the cross-country focus groups by Fritz *et al.*,^372^ along with the idea that DACCS could help to “buy time” for deeper decarbonization and transitioning the world to renewable energy. Indeed, according to Scott-Buechler *et al.*,^340^ it is mostly the global benefits for tackling climate change, rather than any environmental benefits at local level, which tend to predominate in focus-group discussions.

The third key concern centers on the potentially controversial associations between DACCS and the fossil-fuel industry. Such associations could be both direct, for instance by carbon captured being used for enhanced oil recovery^343,392,393^ or that the rollout of DACCS serves as an excuse not to reduce emissions.^340,362,368,372,378,382^ On this point, Low *et al.*^362^ identified significant support in their global set of focus groups for polluting industries to be required to fund research and innovation into DACCS, akin to the “polluter pays principle”.

Questions over the “moral hazard” of DACCS^363,366,369,378,380^ and its perceived failure to address the root cause of climate change^357,366^ are recurring – another point of overlap with expert perceptions.^353,391^ Through their mediation analysis, Campbell-Arvai *et al.*^380^ found evidence for a potential moral hazard, where reading about DACCS (or other CDR approaches) reduced support for mitigation. This relationship is mediated by declining belief in the threat of climate change – and was stronger for individuals holding conservative political views (in the United States). Accordingly, publics tend to stress the need for DACCS to be coupled to renewable energy, also considering the expected high energy use requirements.^357,376,388^ The German publics participating in the vignette experiment by Merk *et al.*^367^ even identified use of renewable energy as more critical than storage considerations for their support of DACCS. At the same time, the evidence of DACCS presenting a moral hazard is not necessarily uniform, with some studies (*e.g.*, ref. 369) finding no such effect.

It is also notable how little discussion there is of social or ethical considerations among publics. Such concerns tend to be de-prioritized in favor of techno-economic and environmental risks^362,382,388^ (see Yang *et al.*^389^ for similar findings for DACCS project developers and financiers). Given that societal considerations only receive attention in a handful of studies, one wonders how much this reflects the implicit focus of the wider literature. McLaren *et al.*,^378^ in their deliberative workshops in the United Kingdom, highlighted concerns about an unfair shifting of risks, onto poorer populations as well as future generations (see also ref. 363). As an example, the topic of job creation and socio-economic impact (*e.g.*, for local communities) is minimally discussed in the wider literature.^334,340,353,362,365,387,394^ Still, there is growing discussion about how DACCS may promote job creation for those first-movers and areas with high government-industry collaborations: drawing on a global set of focus groups,^362^ specifically identify China, India, Saudi Arabia, Norway, and Switzerland as examples, noting these countries represent a “North-South crosscutting plurality” where extractive industries are strong. Using conjoint analysis, Scott-Buechler *et al.*^340^ demonstrated that job creation increased support for local DAC projects. At the same time, when asked to rank the importance of six criteria for future CDR deployment, one of which was job creation, Cox *et al.*^387^ found that this factor took a backseat to durability, *i.e.* the low probability of carbon leakage, and benefits for biodiversity. While assessing options and pathways for CDR deployment in the United States, a peer-reviewed report (“Roads to Removal”) has stressed the importance of equity and justice considerations for engagement, design, siting, and management decisions on CDR.^395^ This took the form of two novel indices: the energy equity and environmental justice (EEIJ) index, and the social vulnerability index. In the case of DACCS, these justice considerations centered on opportunities to reverse job-loss trends and decisions to opt for siting projects in less vulnerable areas that are better able to engage.

To our knowledge, there are few calculations of prospective job gains from scaling-up DACCS. One, a grey-literature report,^396^ projects that a DAC plant with capacity of 1 megatonne would generate 3500 jobs in the United States along the supply chain, indirectly yielding at least 300 000 (potentially high wage) jobs in construction, engineering, manufacturing, operations and maintenance for the sector as a whole. Larsen *et al.*^396^ noted that potential opportunities could be most closely targeted to workers (and communities) in legacy industries, like cement, chemicals, and natural gas – this echoes the EEIJ index from Pett-Ridge *et al.*^395^ A recent presentation by the Rocky Mountain Institute,^397^ drawing on data from three active DAC companies, estimated a workforce of 400 000–500 000 would be needed to remove 1 gigatonne of CO_2_ per year. Such jobs would principally be in the construction sector. Though for CDR in general, Pett-Ridge *et al.*^395^ also calculated that more than 440 000 long-term jobs may be created if the aim of removing 1 gigatonne of CO_2_ per year were achieved – they note this is nearly five times as many jobs as have been lost in the coal industry since 1990.

Under-examination of societal considerations also extends to the limited attention to governance for DACCS deployment. There is some research on this nascent topic, all published in the last year.^340,362,366,387^ For instance, Fritz *et al.*^366^ identified strong, recurring emphasis in their focus groups on the need for community consultation, in forms such as townhall meetings, deliberation, and community surveys. Calls for such engagement reflected a desire to engage with questions and decisions on siting, storage, design, and risk management of DACCS projects; this was reaffirmed in the United States focus groups (and conjoint analysis) by Scott-Buechler *et al.*,^340^ where engaging communities was seen to serve the aims of avoiding conflict, engaging with opposition, and trying to secure support. For the first time, Cox *et al.*^387^ used a survey in the UK to examine the impact of sociotechnical systems on attitudes towards DACCS: such systems varied in terms of governance (top-down *versus* bottom-up) and market approach (planned *versus* liberal economy). The authors, however, identified no significant differences between the different approaches for public attitudes towards DACCS. As the first research of its kind, this subject remains an important area for further study.

In terms of individual characteristics of DACCS support, those expressing a stronger sense of climate severity and urgency, *i.e.*, viewing the climate crisis as an imminent threat, or climate concern tend to be more supportive.^340,357,360,368,369,372,386,387^ Similarly, Merk *et al.*^367^ revealed, through multifactorial vignette experiment in Germany, that individuals perceiving a stronger moral obligation to mitigate climate change were more likely to support DACCS. Support for DACCS is further linked to the belief and optimism in technology as a solution^386^ and trust in responsible actors or institutions.^360,368,386^ For offshore forms of DACCS, individual views of marine environments as more adaptable, more manageable, and less fragile are tied to support. Interestingly, using an experimental design where some individuals were asked to read descriptions more thoroughly, and that they would be tested, Carlisle *et al.*^359^ found those engaging in more “reflective” thinking tended to view DACCS more favorably.

Regarding demographics, younger individuals are frequently found to be more supportive;^340,367,368,380,384,386,387^ men are more likely to be supportive as well.^340,360,368,385,386^ However, there is also research that finds no such effects (*e.g.*, ref. 367 and 369) for age and gender;^360^ for age;^380^ for gender). Bellamy *et al.*^376^ also established that their male-only focus groups expressed greater concerns about possible costs of DACCS than a female-only counterpart. Education also plays a role, while this is more unclear: those more highly educated, depending on the context, can be less supportive,^367^ more supportive,^368^ or not different from others.^340,360^ Political ideology was also shown to be meaningful by Sweet *et al.*^384^ and Cox *et al.*,^387^ with those expressing more conservative views less likely to be supportive. Again, we underscore that these studies of public perceptions are uniformly from Western, highly developed countries. Recently, utilizing a cross-country set of representative surveys across the global North and global South, Sovacool *et al.*^371^ highlighted that individuals self-identifying as members of ethnic or minority groups were more likely to be supportive of DACCS (and other climate interventions); they also point to spatial differences, whereby those in urban areas were more likely to be supportive, and those in rural areas the least. Of note, regarding ethnicity in the United States, Scott-Buechler *et al.*^340^ found that white participants were more likely to be supportive of DACCS than others.

## 6. Monitoring, reporting, and verification for DACCS

**Fig. 28:**
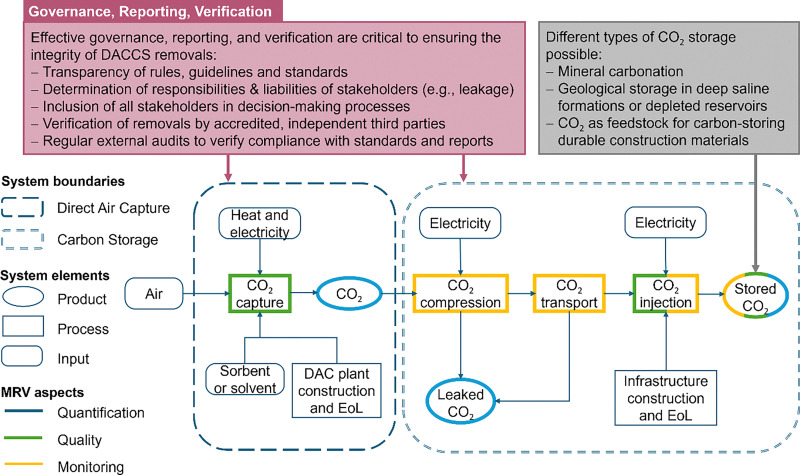
System boundaries, processes, inputs and outputs of DACCS systems. The MRV aspects quantification (blue – accurately measuring and calculating the amount of CO_2_ removed from the atmosphere and securely stored), monitoring (yellow – systematic tracking and collection of data over time to assess the performance and stability of CDR activities) and quality (green – refers to the degree to which a CDR activity meets established standards for additionality, effectiveness, durability, and integrity) are highlighted across key stages. Governance, reporting, and verification apply across the entire system to ensure transparency and credibility.

Monitoring, reporting, and verification (MRV, sometimes also referred to as measurement, reporting, and verification) is fundamental for CDR deployment at scale. MRV as a concept refers to a multi-step process of measuring and quantifying impacts of CDR activities – crucially, CO_2_ removed – and subsequent communication, inventorying, and validation of the veracity of removals claims.^398^ Operationalized MRV frameworks are often designed for different spatial scales, for example project-level activities (*e.g.*, ref. 399 and 400) and national-level accounting toward climate targets.^401^ Additionally, MRV must consider the whole value chain of a CDR activity. The direct air CO_2_ capture and storage process can be described in two distinct stages: the capture of CO_2_, and the subsequent storage of the CO_2_ captured. Across these stages, there are different steps where MRV-relevant activities take place (Fig. 28).

This section presents the state of (scientific) knowledge on MRV of DACCS, which is, frankly, scarce. Using a systematic search and screening approach, it focuses primarily on mapping and synthesizing the evidence in the peer-reviewed literature that answers two questions:

(1) What units can be quantified and

(2) How can these be quantified?

As such, the section emphasises the “M” step of “MRV”, though implications on the “R” (reporting”) and “V” (verification) are also discussed. The findings from the peer-reviewed scientific literature are complemented by a systematic evaluation of the existing MRV protocols for DACCS. These protocols give insight in the current requirements that must be met, including minimum standards for quantification, to certify DACCS removals.

To search the peer-reviewed literature, the DACCS keyword query developed by Lueck *et al.*^24^ was combined with MRV-specific keywords. The final search query is simplified as: (〈DACCS keywords〉 AND 〈MRV keywords〉) and includes literature published before August 2024. We used a pre-defined set of inclusion/exclusion criteria to screen for relevance. We supplemented our results with grey literature, because there is often a time lag between the latest scientific developments and the peer-reviewed literature.

### 6.1. Overview of MRV topics in the DACCS literature

Six primary studies were identified that contained relevant information on MRV while being specific to DACCS, additional to ten studies on carbon capture and storage (CCS) more broadly, with implications for DACCS (Table 11). The limited number of results is likely a result of our very focused search query but also suggests the scientific underpinning for MRV may still be sparce. The review also identified at least 14 papers focussing on life-cycle assessment (LCA) of DACCS, which were excluded here as they are addressed in Section 4.2.

**Table 11:** Peer-reviewed DACCS and CCS MRV studies identified in the scientific literature

Authors, year	Title
Lackner & Brennan, 2009^402^	Envisioning carbon capture and storage: expanded possibilities due to air capture, leakage insurance, and C-14 monitoring
Smal *et al.*, 2014^403^	TG-FTIR measurement of CO_2_–H_2_O co-adsorption for CO_2_ air capture sorbent screening
McGivern *et al.*, 2023^404^	Improved apparatus for dynamic column-breakthrough measurements relevant to direct air capture of CO_2_
Wolf *et al.*, 2004^405^	*In situ* observation of CO_2_ sequestration reactions using a novel micro reaction system
Haefeli *et al.*, 2005^406^	Important accounting issues for carbon dioxide capture and storage projects under the UNFCCC
Jan Roman *et al.*, 2012^407^	Gas permeation measurement under defined humidity *via* constant volume/variable pressure method
Cui *et al.*, 2016^408^	Localization of CO_2_ leakage from a circular hole on a flat-surface structure using a circular acoustic emission sensor array
Ikeda & Tsuji, 2017^409^	Robust subsurface monitoring using a continuous and controlled seismic source
Möller & Schloemer, 2021^410^	Determining soil CO_2_ threshold levels by means of common forecasting methods as part of near-surface monitoring for carbon sequestration projects
De Fommervault *et al.*, 2022^411^	Real-time and continuous monitoring of submarine volcanism with a seaexplorer glider: perspective for carbon storage monitoring
Fawad & Mondol, 2022^412^	Monitoring geological storage of CO_2_ using a new rock physics model
Bakelli *et al.*, 2024^413^	A feasibility study on the pressure monitoring above the injection zone for CO_2_ geological storage in the Uinta Basin, USA
Nii *et al.*, 2024^414^	A conceptual subsurface risk management and measurement, monitoring and verification design for an offshore carbon capture and storage site in Japan
Premadasa *et al.*, 2024^415^	Towards energy-efficient direct air capture with photochemically-driven CO_2_ release and solvent regeneration
Stamberga *et al.*, 2024^416^	Direct air capture of CO_2_*via* reactive crystallization
Iyer & Smith, 2024^417^	Impact of cement composition, brine concentration, diffusion rate, reaction rate and boundary condition on self-sealing predictions for cement–CO_2_ systems

The six papers with a DAC or DACCS focus did not indicate a specific study site, likely because three used laboratory experiments to investigate the DAC process and three were qualitative studies on specific topics with MRV relevant insights, such as that providing guidance on how to improve the safety and verification of underground storage.^402^ The laboratory experiments provided insights on quantification aspects of MRV, more precisely on the capture process: the adsorption capacity and capture efficiency. Out of the ten papers on only CCS, four focused on specific study locations (Japan, Mayotte – a French overseas department, USA, and 4 European countries). In half of the CCS papers, techniques for monitoring CO_2_ storage and leakage detection are main topics. The topics of removal quality and governance are only discussed in one paper each.

### 6.2. Quantification of CO_2_ removals in DACCS

There is evidence in the literature that directly measuring the amount of CO_2_ captured and stored from direct air capture is possible, while studies show that using commercially available mass flow meters can do so with smaller than 0.28% mean absolute measurement errors.^418^ Relative to other CDR methods, CO_2_ removal can be measured for DACCS with comparatively high confidence and low MRV risk, in part because the capture process takes place in a closed system.^398^ Additionally, many existing technologies for measuring and monitoring the transport and storage of CO_2_ can be reused or adapted from industrial carbon capture, utilization, and storage (CCUS). Parameters such as pressure, temperature, and composition of a CCS reservoir can be measured and tracked over time,^419^ allowing quantification of the amount of CO_2_ present in subsurface reservoirs.

In practice, simply measuring CO_2_ flows across the boundaries of a DAC project may be the more appropriate approach for quantifying gross and net CO_2_ removals. Existing equipment and technology that can be used to determine the amount of CO_2_ captured include Coriolis flow meters while other methods have been discussed in the literature too. Smal *et al.*,^403^ used a combined thermogravimetry-Fourier transfer infrared spectroscopy (TG-FTIR) system to measure water and CO_2_ adsorption capacity of sorbents. They developed a method for quantitatively determining the amount of CO_2_ and H_2_O co-adsorbed from ambient air on small sorbent samples. Using a gas pulse-based calibration, CO_2_ capacity could be determined with ∼5% accuracy. Similarly, McGivern *et al.*^404^ developed an improved apparatus for dynamic column-breakthrough measurements that allows for application to adsorbents at a range of temperatures, pressures, and relative humidities.

Approaches for measuring and monitoring stored CO_2_ depend on how it is stored, *e.g.*, *via* an *ex situ* or *in situ* mineralisation process. With *ex situ* mineralisation the method suitability may depend on accuracy and precision needs or be limited by instrument availability and cost. Available tools and analyses that may be used included thermogravimetric analysis (TGA), powder X-ray diffraction, FTIR spectroscopy, volumetric calcimetry, and loss on ignition (LOI).^420^ For *in situ* mineralisation, for example in subsurface geological formations, regulations around CO_2_ storage sites in some countries already set a precedent for how baseline measurements of CO_2_ storage should be conducted, *e.g.*, before injection, and monitored. Surface system inputs are easily measured, and technology is available to measure subsurface injection of CO_2_ in storage reservoirs. These include geophysical or geochemical monitoring approaches (*e.g.*, chemical tracers) and seismic monitoring approaches (*e.g.*, surveys, modelling, gravimetry, and geoelectrical approaches).^405,409,410,412^

The most significant uncertainties relevant to the MRV process for DACCS are around the accounting for the overall estimation and efficiency of (net) CO_2_ removals. A strong carbon accounting framework is an essential building block for MRV and building up a broader CDR ecosystem.^402^ This includes accurate information on the carbon intensity of project inputs (feedstock, energy) and outputs (*e.g.*, wastes and their treatment). This means DACCS MRV needs to tie into existing carbon accounting schemes and standards. The accounting issue is closely linked to the system boundaries and assumptions made when conducting the overall life cycle assessment for a DACCS activity (see Section 4.2), while drafting sound accounting mechanisms also relates to technology readiness. For example, liquid and solid sorbent DAC are comparably well-researched pathways and the understanding of material and energy inputs and outputs is well understood, allowing development of a more exhaustive accounting framework. The more novel pathways, such as membrane techniques, are still in an experimental phase (Section 3.10), and as a result, less is known about the overall technical process, potential material, energy, and infrastructure inputs/outputs, hindering development of accounting frameworks (although this should be solved as technologies move up the TRL ladder).

### 6.3. Outlook for development of DACCS MRV

Robust MRV systems are fundamental to including DACCS in a well-functioning carbon market, thus to deployment at scale. A crucial step in this is developing the standards for demonstrating and proving a CDR activity has taken place, and the methodologies that enable these activities to be quantified and certified. Three DACCS protocols are currently available or under development, serving the current voluntary market (Table 12). One protocol is specific to the capture part of DACCS, one for the storage part, while one includes both. Additionally, 11 protocols are available for CCS. In 2024, Climeworks Orca was the first DAC project to be certified under the Puro.Earth Standard.^421^

**Table 12:** Overview of existing DACCS protocols

Name of protocol	Quantification guidance
Climeworks^421^ (direct air capture, collaboration with Puro.Earth)	• CO_2_ is measured upstream of storage site, post-capture.
	• For activities with TVSA adsorption process, solid sorbent material, and *in situ* storage or mineralisation.
	• Based on ISO standards for quantifying GHG emissions.
	• Calibration of measurement devices should allow uncertainty of 5% or better.
	• Equations provided for calculating CO_2_ injected during monitoring period, and GHG emissions from project operations, construction, and disposal.
Carbfix^400^ (transport + storage) (collaboration w/Puro.Earth)	• CO_2_ is measured at injection wellhead of the storage site
	• Project emissions subtracted from stored CO_2_ quantities
	• Suitability characterization and subsurface monitoring plans required before injection
	• Field sampling and reservoir models can be used for monitoring injection; mass-balance calculations can also be used to quantify injected CO_2_ with reactive tracers to lower uncertainties
	• Any CO_2_ released into the atmosphere after the injection measurement point is subtracted from the CDR credited
Isometric^399^ (direct air capture + storage)	• Calculation of CO_2_-equivalent stored requires measurements in CO_2_ injection stream or within a carbonate solution and total mass of injectant
	• Multiple options given for durable storage of CO_2_ with separate modules
	• Equations provided for calculating net CO_2_-equivalent removed and stored
	• Equations provided for calculating energy usage, transport emissions, and other process emissions

On the national level, protocol development for DACCS has started in Europe, the United States, and Canada.^422^ The European Union, for example, is working on a new carbon removal certification framework (CRCF). The CRCF includes DACCS under its definition of permanent CDR.^423^ Currently, there are no IPCC guidelines for the capture part of DACCS. The IPCC guidelines serve as the basis for national inventory accounting under the United Nations framework convention on climate change (UNFCCC) and are required counting activities towards national climate targets. IPCC national GHG inventory guidelines are, however, available for geological CO_2_ storage. An IPCC methodology report on carbon dioxide removal technologies, and carbon capture, utilization, and storage (CDR/CCUS) is underway and will include DAC. It is expected to be completed by 2027.^424^

Finally, an important research gap remains around the cost of MRV. Little data is available on the topic across CDR methods, including DACCS (Mercer and Burke, 2024). WIth DACCS, perceived challenges influencing cost estimates for MRV link back to regulatory uncertainty and lack of standardization around MRV. Several studies have begun to estimate the costs of monitoring of geological carbon storage based on CCS projects, providing insights directly relevant to the development of MRV systems for DACCS. For example, Elsayed & Okoroafor (2024) estimate that total monitoring costs over a 25-year lifecycle can reach approximately $10.21 million. Their assessment considers a range of monitoring technologies including 4D seismic surveys, crosswell and 2D seismic, interferometric synthetic aperture radar (InSAR), GPS, and tilt measurements. Wu *et al.* (2023) highlight that the monitoring costs during CO_2_ injection are strongly influenced by the assessed risk levels of leakage. In low-risk leakage scenarios, investments tend to be more volatile and sensitive to the precision of monitoring technologies, while in high-risks scenarios costs increase due to the need to minimize CO_2_ leakage and ensure detection reliability.

## 7. DACCS deployment in future scenarios

Following from the discussions above, a key remaining question is how much direct air capture capacity may be needed to achieve the Paris climate goals, *i.e.*, how much direct air capture will be deployed in future scenarios given energy inputs, costs, and carbon removal potential. This requires an assessment of potential future developments of emissions, energy demand and supply, as well as economic and technical developments. To start answering this question, we reviewed the deployment of DACCS in the integrated assessment modelling literature. Integrated assessment models are forward looking optimisation models, that consistently and comprehensively describe the energy system with high technological detail and describe the transformation necessary to achieve a given climate target at the lowest cost achievable, constrained by an allowed technology mix and set of policies and assumptions They combine descriptions of the climate system, economy, and a technology system to answer questions relevant to policymaking and science.

The coverage of DACCS in integrated assessment models (IAMs) has been growing in recent years, but it remains far from a standard technology to be included in IAM scenarios. There are three categories of scenarios (labelled as categories C1–C3, see Tables 3.3 and 3.5 in ref. 425) that limit global mean temperature increase to likely below 2 °C or lower in the so-called AR6 database,^426^ the set of integrated assessment modelling scenarios used for the 6th assessment report (AR6) of the intergovernmental panel on climate change (IPCC). 146 out of the 541 IAM scenarios in these three categories included DACCS, meaning the majority of scenarios excluded DACCS in the technology mix. The 146 scenarios show considerable agreement on early DACCS deployment, with a median deployment amount of only 23 MtCO_2_ year^−1^ in 2050. In almost 90% of the 146 scenarios, DACCS deployment remains below 1 GtCO_2_ year^−1^ until 2050. Conversely, separate IEA study investigating a pathway to global net-zero emissions by 2050 shows DAC deployment of almost 1 GtCO_2_ year^−1^ by 2050 at CO_2_ prices of up to 250 $ per tCO_2_.^427^ An early (2019) assessment by Realmonte *et al.*,^356^ suggested that the 2050 DACCS deployment may be approximately 3 Gt per a in the period 2040–2070 and 20 Gt a^−1^ in the period 2070–2100, with the caveat that comparatively low DAC costs of between 50 and 350 US$ per tonne CO_2_ were assumed.

**Fig. 29:**
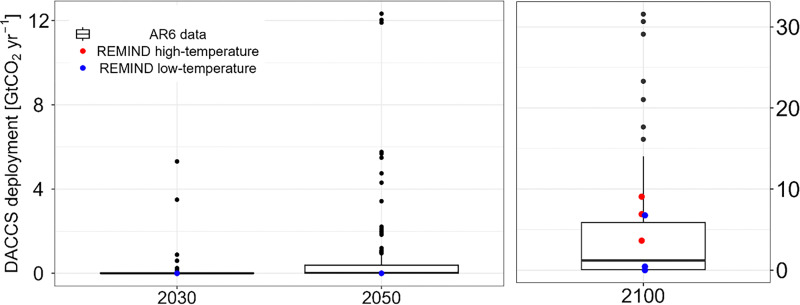
DAC deployment in 2030, 2050, and 2100 in the AR6 database scenarios (black dots and boxplot) and in the REMIND scenarios using liquid solvent (red) or solid adsorbent (blue) DAC. DAC cost and energy parametrisation as per the supplementary Excel file. Note the different deployment scales on the left (2030 and 2050) and right (2100) panels.

**Fig. 30:**
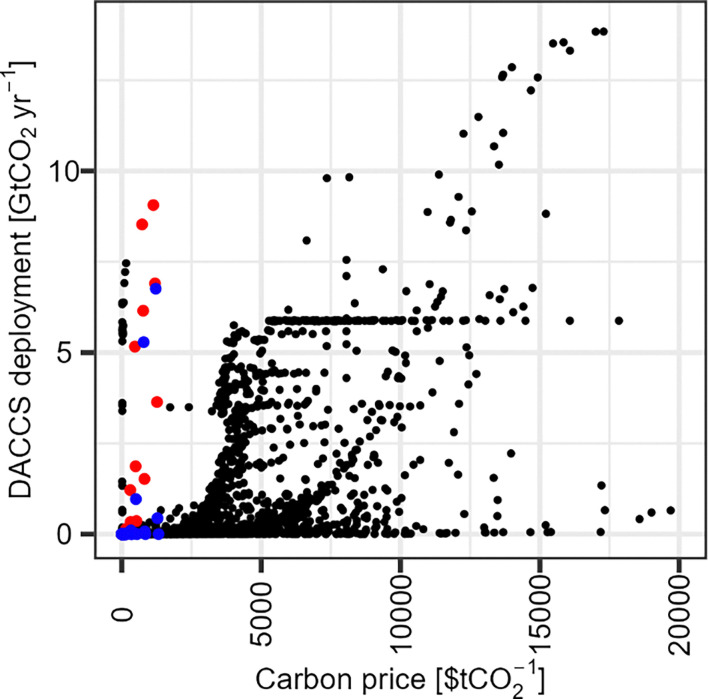
DAC deployment *versus* carbon price in the AR6 database scenarios (black) and in the REMIND scenarios using liquid solvent (red) or solid adsorbent (blue) DAC. DAC cost and energy parametrisation as per the supplementary Excel file.

The projected long-term deployment in the AR6 database, however, shows much more variance. 75% of the 146 AR6 scenarios remain below 6 GtCO_2_ year^−1^, but a small number of scenarios show very large amounts of 10–30 GtCO_2_ year^−1^ in 2100 (Fig. 29). The models also agree that significant DACCS deployment starts only at very high carbon prices of above 2000 $ per tCO_2_ (Fig. 30). A possible reason for the correlation with very high carbon prices beyond the costs of DAC could be the late deployment of DAC in the second half of the century, where corresponding carbon prices usually continue to rise in most models, meaning DACCS deployment happens in parallel with rising prices, not *per se* as a result of further rising prices beyond the costs of DAC (but driven by other model constraints).

We complemented the existing models to understand how much DACCS will be deployed with the DACCS costs highlighted in Section 4.1. To this end, we analysed DAC deployment in the IAM REMIND 3.4.0,^428,429^ using a parameterization derived from the technology and TEA review in Sections 3 and 4.1 in this study. We studied low-temperature (solid adsorbent) and high-temperature (liquid solvent with mineral looping) DAC in separate model realisations, without competition between the two, and including a mean performance (cost and energy consumption) estimate, a more pessimistic and a more optimistic parameterization (derived from Section 4.1, provided in the supplementary Excel file). In short, for liquid absorbents the mean energy requirements were 4.06 GJ per tCO_2_ heat and 0.66 GJ per tCO_2_ electricity; for solid sorbents, the mean energy requirements were 5.9 GJ per tCO_2_ heat and 0.32 GJ per tCO_2_ electricity. For capital expenses, for liquid absorbents respectively solid adsorbents, starting values of 198 and 1419 S per tCO_2_ were inputted, while technological learning was considered, meaning that the capital costs reduce gradually with deployment (see supplementary Excel file for further details). However, energy consumption cannot learn in REMIND and was therefore kept constant over time. We used the NOAK energy requirements, which leads to an overestimation of DAC deployment early on.

Regardless of the favourable energy consumption assumed for early DAC, the model realisations suggest that in scenarios likely remaining below 2 °C (category C3) and in scenarios that remain below 1.5 °C with low overshoot (category C1), there was no or only very limited (<0.1 GtCO_2_ year^−1^) DAC deployment until the end of century, even in the most optimistic parametrisation. This low value results from the implementation of the climate target in REMIND, where the shape of the carbon price trajectory is fixed, and absolute levels are adjusted such that the cumulative carbon budget is met. As a result, in scenarios with low or no overshoot, the carbon price is kept constant after the peak budget is reached, which prevents very high long-term carbon prices (the carbon price never exceeds 450 $ per tCO_2_).

The further discussion will, therefore, focus on the C2 scenario, consistent with 1.5 °C temperature increase in 2100 with high overshoot, where the carbon price continues to increase exponentially until the end of the century. The cumulative carbon budget from 2020 to 2100 is limited to 400 GtCO_2_, which was reported as giving a 67% likelihood to remain below 1.5 °C in 2100 in the AR6^430^ but may be exceeded before 2100. We note that more recent updates of the budget see only a likelihood below 50% to reach 1.5 °C,^431^ while to remain comparable to the AR6 database we here used the 400 GtCO_2_ budget. Assumptions regarding socio-economic drivers such as population, gross domestic product, and energy demand follow medium estimates as defined in the shared socio-economic pathway SSP2.^432^

The REMIND results confirm the considerable carbon prices needed to make DAC economically competitive (Fig. 30). In the C2 scenario, CO_2_ prices increase to around 500 $ per tCO_2_ in 2080 and above 1000 $ per tCO_2_ in 2100. This leads to a late scale-up of DACCS only in the second half of the century, with deployment in 2100 of 6 (min–max: 3.6–9) GtCO_2_ year^−1^ for liquid solvent DACCS and 0.4 (min–max: 0–6.8) GtCO_2_ year^−1^ for solid adsorbent DACCS. However, compared to the AR6 scenarios, high DACCS deployment above the Gt scale is reached already for carbon prices around 300 $ per tCO_2_, at least for liquid solvent DACCS (Fig. 30). For solid adsorbent DACCS, the best estimate is comparable to the AR6 database range and only reaches the Gt scale with the more optimistic parameterization.

These results suggest that DACCS needs significant cost improvements to become a competitive CDR option. If the willingness-to-pay is high enough, DACCS could be scaled to high deployment at least in the second half of the century. However, when interpreting these model results, the reader needs to bear in mind that IAMs consider economically optimal solutions only, where technologies like DACCS are deployed as soon as they are economically competitive. In the models, there is usually no consideration of market failures, or social or political opposition to high carbon prices. Some models including REMIND also use perfect foresight, *i.e.*, they know how high future carbon prices will be, leading to earlier deployment to realize learning and enable higher DACCS levels later in the century. These effects may lead to a higher DACCS deployment in the model than may be seen in the real world.

Finally, there are also some uncertainties that could lead to higher DACCS deployment than currently foreseen in models. DACCS demand could be higher in the real world than in the models either due to other CDR options not delivering as expected, or due to higher CDR demand in general. Especially land-based CDR options such as reforestation and bioenergy with CCS (BECCS) could suffer more from climate damages than currently envisaged. In addition, the amount of bioenergy that can be supplied sustainably is highly uncertain even without considering higher climate damages.^433^ Other CDR options currently discussed that are more independent of land and climate change, such as enhanced weathering of rocks or ocean alkalinisation are still in their infancies, leading to large uncertainties regarding their availability as well.^434–436^ Higher CDR demand in general could result from uncertainties in the carbon budget. Mitigation pathways most often use a carbon budget that remains below 1.5 °C with a likelihood of 67%, as was also done in this study. However, as noted already, more recent updates corrected the remaining carbon budget downward by about 130 GtCO_2_,^414^ resulting in the need to reduce emissions faster or compensate more *via* CDR. In addition, even a 33% chance of exceeding 1.5 °C could be considered too risky given the magnitude of potential climate damages. For the case of temperature reductions back to 1.5 °C after a temporary exceedance, Schleussner *et al.*^8^ argued for the need of several hundred gigatonnes preventive CDR capacity that may be necessary to hedge against the risk of strong earth-system feedbacks. A precautionary strategy could be to invest into research and development of potential large-scale CDR options such as DACCS to have them available at lower cost in case more net-negative emissions are needed.

## 8. Conclusion

### 8.1. Research landscape and technology development

In conclusion, DACCS the research landscape is growing quickly and diversifying from mere materials development to more technology/process development including a much-needed growth in technology and systems analysis studies. More social sciences and humanities studies are gradually emerging, while much more is needed to inform robust implementation planning and policy making, especially focusing on regions other than Europe or North America.

There is strong diversification visible in the DAC technology space, with regular publication of new approaches, which, at least at lab scale, suggest significant improvements in (mostly energy) performance, attempting to address the currently high energy consumption for CO_2_ separation from air. This review distinguished between eight different technology categories, while other reviews have identified even more. Meanwhile, first and second-generation technologies have scaled to higher TRL, with solid adsorbent and mineral looping technology having reached TRL 9, and liquid solvent with mineral looping expected to reach TRL 9 in 2025. A number of second-generation technologies have also progressed to the pilot plant stage (TRL 6 or 7). Key imperatives for further research and development include comprehensive investigations into materials degradation, deactivation, and other losses, including measurement, quantification, and mitigation of substances potentially harmful to humans and environment; and swift advancement of all technology categories to the (small) pilot scale, to understand their performance as an integrated process, identify bottlenecks, corroborate (or reject) lab-scale performance claims, and identify solutions to difficulties found.

### 8.2. Technology performance

The need for performance corroboration and for plotting realistic R&D and upscaling pathways is confirmed by the lagging progress in reducing energy consumption. Our synthesis highlighted that energy consumption reported in the scientific literature remained stable over the past two decades, and the expected breakthroughs haven’t yet materialised. If anything, the range of reported energy consumption has widened upward. This also translates into stable cost predictions as per the scientific publications. While some startups claim fantastically low costs for their technologies, beating the 100 US$ per tonne CO_2_ captured target, a credible pathway to such cost levels is yet to be confirmed by the scientific community. More plausible cost pathways project 100–600 US$ per tonne net removed at a scale of 1 Gtonne CO_2_ removal per year. Additionally, current costs are substantially higher, ranging from 400 to over 2500 US$ per tonne removed. It is unrealistic to believe this will be driven down by R&D only, but instead needs to include learning by doing – facilitated by commercial-scale deployment – in tandem with R&D.

Positively, the carbon balance for (existing as well as future) direct air capture technologies was found well net negative, with most studies reaching a net carbon removal efficiency of over 50% using electricity from current average European electricity grids (at a carbon intensity of 350 kgCO_2_ per MWh); while all studies reached 50% removal efficiency at a UK average grid intensity of 200 kgCO_2_ per MWh. The life cycle analysis in this review also showed that even at a scale of 1 Gtonne CO_2_ per year deployed for a 2050 2 degree C scenario (SSP2/RCP2.6), the global environmental impact is limited: on most environmental indicators, 1 Gtonne CO_2_ per a DACCS increases global impacts by less than one percent, while only for particulate matter formation, the increase is four percent, while the LCA did confirm the high energy needs for DACCS, with a 5–6% rise in cumulative energy demand globally. Yet, a major caveat exists in local environmental impacts, on which topic no studies were found. It is, therefore, unknown what the impacts on local environment or health will be, if any, which needs addressing imminently.

### 8.3. Upscaling & IAMs

Evaluation of direct air capture uptake in integrated assessment models (here, the AR6 database and new REMIND evaluations), does present a stark warning. At current cost and carbon price levels, DACCS deployment will be very limited: almost 90% of the AR6 scenarios that do include DACCS show deployment levels well below 1 GtCO_2_ year^−1^ until 2050 with a median deployment level of only 23 MtCO_2_ year^−1^, contrary to earlier studies that estimated DACCS deployment between 1 and 3 GtCO_2_ year^−1^ by 2050. DAC uptake starts to show significant levels only in the second half of the century, when carbon prices have moved beyond 2000 US$ per tonne CO_2_. However, newly produced REMIND results show scaling of DAC already at lower carbon prices around 300 US$ per tonne CO_2_. This may indicate that the very high carbon prices in AR6 scenarios which are beyond cost assessments in the literature are rather due to the slow scale-up of DAC and the continuous carbon price increase in most models. Still, significant cost improvements are necessary to make DAC a competitive CDR option.

Given that new technologies need learning from deployment to lower costs, this presents a chicken and egg problem, and strong government support in the form of compliance market development, subsidies, or investment tax incentives (additional to carbon emission taxes as was done for solar and wind energy) is imperative to ensure early DAC economic feasibility, help costs decline, and reach carbon market price parity. Given the risks and uncertainties that all CDR options face, from reversibility and permanence to environmental sustainability issues, DAC development puts one more CDR option on the table, which helps reduce the risk of insufficient or unsustainable CDR supply. Alternatively, DAC may still play a smaller role for niche applications, for example, for CO_2_ production at remote locations (*e.g.*, the Kona Hawaii modules delivered by Global Thermostat, Table 13).

### 8.4. Policy, MRV, and public perception

Clearly, policy making needs to step up if DACCS is to be a serious component of the climate change mitigation technology mix. While previous US incentives like the inflation reduction act were hopeful, this review highlights that much stronger and long-term DACCS/CDR policy is needed to create the needed markets and financial incentives, in line with findings from the IAMs. This also includes legislating for large-scale CO_2_ transport and storage, low carbon energy supply in locations where this is most opportune, and integration with other elements of the energy transition and route to net-zero. Equally, it is imperative for public support that policies foster equity and social justice, and make climate change mitigation work for all, instead of for few. In that light, the massive inflow of Western corporate and investor money into direct air capture may present challenges that robust policy frameworks need to help overcome. Currently, there appears to be sufficient support for DACCS, especially among those with a higher awareness of the climate emergency, but this can quickly erode with unjust or inequitable DACCS implementation. Finally, there are few MRV protocols to certify the carbon removal performance of DACCS projects, hampering the development of well-functioning markets. While MRV protocol development can be left to commercial parties, robust frameworks governing such MRV protocols are needed to ensure trust in DACCS as a carbon removal strategy.

## Author contributions

Mijndert van der Spek: conceptualisation, data curation, formal analysis, methodology, visualisation, writing – original draft, writing – review & editing. André Bardow: conceptualisation, writing – review & editing. Chad M. Baum: conceptualisation, formal analysis, writing – original draft. Vittoria Bolongaro: conceptualisation, data curation, formal analysis, investigation, software, visualisation, writing – original draft. Vincent Dufour: conceptualisation, data curation, formal analysis, visualisation, writing – original draft. Carla Esch: conceptualisation, data curation, formal analysis, writing – original draft. Livia Fritz: conceptualisation, data curation, formal analysis, writing – original draft. Susana Garcia: conceptualisation, methodology, visualisation, writing – original draft, writing – review & editing. Christiane Hamann: conceptualisation, data curation, formal analysis, visualisation, writing – original draft. Dianne Hondeborg: conceptualisation, data curation, formal analysis, visualisation, writing – original draft. Ali Kiani: conceptualisation, data curation, formal analysis, visualisation, writing – original draft. Sarah Lueck: conceptualisation, data curation, formal analysis, software, visualisation, writing – original draft. Shrey Kalpeshkumar Patel: conceptualisation, data curation, formal analysis, visualisation, writing – original draft. Shing Bo Peh: conceptualisation, data curation, formal analysis, visualisation, writing – original draft. Maxwell Pisciotta: conceptualisation, data curation, formal analysis, visualisation, writing – original draft. Peter Psarras: conceptualisation, writing – review & editing. Paola Alejandra Saenz-Cavazos: conceptualisation, data curation, formal analysis, visualisation, writing – original draft. Ingrid Schulte: conceptualisation, data curation, formal analysis, visualisation, writing – original draft. David Shu: conceptualisation, data curation, formal analysis, visualisation. Qingdian Shu: conceptualisation, data curation, formal analysis, visualisation, writing – original draft. Benjamin Sovacool: conceptualisation, data curation, formal analysis, visualisation, writing – original draft. Jessica Strefler: conceptualisation, data curation, formal analysis, software, visualisation, writing – original draft. Sara Vallejo Castaño: conceptualisation, data curation, formal analysis, visualisation, writing – original draft. Jin-Yu Wang: conceptualisation, data curation, formal analysis, visualisation, writing – original draft. Matthias Wessling: conceptualisation, writing: review & editing. Jennifer Wilcox: conceptualisation, writing: review & editing. John Young: conceptualisation, data curation, formal analysis, visualisation, writing – original draft. Jan C. Minx: conceptualisation, data curation, formal analysis, methodology, visualisation, writing – original draft, writing – review & editing.

## Conflicts of interest

The authors declare no conflict of interest.

## Supplementary Material

EE-018-D5EE01732G-s001

EE-018-D5EE01732G-s002

EE-018-D5EE01732G-s003

## Data Availability

The data supporting this article have been included as part of the supplementary information (SI). Supplementary information: the review protocol has been included as a SI Word document, while part of the review data, and the REMIND techno-economic input data are included as supplementary spreadsheets. The REMIND source code for the modelled scenarios can be retrieved from: https://doi.org/10.5281/zenodo.15830280. See DOI: https://doi.org/10.1039/d5ee01732g.

## Appendices

### Appendices

**Table 13:** Overview of pilot, demonstration, and commercial DAC plants deployed as per August 2024

Developer	Project name	Project location	Start-up year	TRL	Main capture material	Ref.
Climeworks	Orca	Iceland	2021	9	Adsorbent	437
Airthena/CSIRO	Airthena DAC Demonstrator	Australia	2020	5	Adsorbent	79
Climeworks	Hinwil	Switzerland	2016	8	Adsorbent	438
Global Thermostat	GT K-Series	United States	2022	8	Adsorbent	439
Hydrocell			2017	6	Adsorbent	93
Carbon Collect		United States	2022	5	Adsorbent	440
Climeworks		Germany	2015	6	Adsorbent	441
Climeworks		Switzerland	2016	6	Adsorbent	441
Climeworks		Iceland	2017	6	Adsorbent	441
Climeworks		Switzerland	2018	6	Adsorbent	441
Climeworks		Italy	2018	6	Adsorbent	441
Climeworks		Germany	2019	6	Adsorbent	441
Climeworks		Netherlands	2019	6	Adsorbent	441
Climeworks		Germany	2019	6	Adsorbent	441
Climeworks		Germany	2019	6	Adsorbent	441
Climeworks		Germany	2020	6	Adsorbent	441
Climeworks		Germany	2020	6	Adsorbent	441
Climeworks		Germany	2020	6	Adsorbent	441
Global Thermostat	GT T-Series	United States	2021	6	Adsorbent	442
Global Thermostat	Kona Hawaii GT T-Series	United States	2023	9	Adsorbent	442
Global Thermostat	GT SRI Pilot 1	United States	2011	6	Adsorbent	441
Global Thermostat	GT SRI Pilot 2	United States	2013	6	Adsorbent	441
Global Thermostat	GT Huntsville Pilot	United States	2018	6	Adsorbent	443
Carbon Engineering		Canada	2015	7	Solvent	441
Heirloom Carbon	Uno	United States	2023	6	Mineral looping	206
Exxon Mobil	Baytown Pilot	United States	2024	6	Adsorbent	444
Avnos	Bakersfield Pilot	United States	2023	6	Adsorbent	445
University of Twente	Twente DAC Pilot	Netherlands	2022	6	Adsorbent	446
Skytree	Cumulus – WUR	Netherlands	N/A	6	Adsorbent	447
Skytree	Cumulus – Fieldless	Canada	N/A	6	Adsorbent	447
Skytree	Cumulus – Neboda Farms	Spain	N/A	6	Adsorbent	447
Skytree	Cumulus – Koppert Cress	Netherlands	N/A	6	Adsorbent	447
DACMA		Brazil	2024	5	Adsorbent	448
DACMA		Brazil	2024	6	Adsorbent	448
CO_2_CirculAir		UK	2024	6	Electrochemical – solvent	
Mission Zero Technologies		UK	2023	6	Electrochemical – solvent	151
