# Supplementary material for: An ecosystem of carbon dioxide removal reviews – part 1: direct air CO2 capture and storage
Source: Energy Environ Sci. 2025 Oct 1;18(22):9713–85. doi: 10.1039/d5ee01732g (PMC12533392; doi:10.1039/d5ee01732g)
Supplement: EE-018-D5EE01732G-s001 [file EE-018-D5EE01732G-s001.pdf]

Supplementary Information (SI) for Energy & Environmental Science.
This journal is © The Royal Society of Chemistry 2025

An Ecosystem of Carbon Dioxide Removal Reviews - Part 1: Direct Air CO_2 Capture and Storage.

A Systematic Review Protocol

Mijndert van der Spek[1], André Bardow[2], Chad M. Baum[3], Vittoria Bolongaro[2], Vincent Dufour-Décieux[2], Carla Esch[4], Livia Fritz[3], Susana Garcia[1], Christiane Hamann[5], Dianne Hondeborg[6], Ali Kiani[7], Sarah Lueck[5], Shrey Kalpeshkumar Patel[8], Shing Bo Peh[1], Maxwell Pisciotta[8], Peter Psarras[9], Tim Repke[5], Paola Alejandra Sáenz-Cavazos[10], Ingrid Schulte[5], David Shu[2], Qingdian Shu[11], Benjamin Sovacool[3,12,13], Jessica Strefler[14], Sara Vallejo Castaño[11], Jin-Yu Wang[1], Matthias Wessling[4,15], Jennifer Wilcox[8], John Young[16], Jan C. Minx[5,17]

[1]Research Centre for Carbon Solutions, Heriot-Watt University, Edinburgh, UK
[2] Department of Mechanical and Process Engineering, ETH Zürich, Zürich, Switzerland
[3]Department of Business Development and Technology, Aarhus University, Denmark
[4]Chemical Process Engineering AVT.CVT, RWTH Aachen University, Aachen, Germany
[5]Department for Environmental Economics and Policy, Potsdam Institute for Climate Impacts Research (PIK), Berlin, Germany
[6]Department of Management, Technology, and Economics, ETH Zürich, Zürich, Switzerland
[7]CSIRO Energy, NSW 2304, Mayfield West, Australia
[8]Department of Chemical and Biomolecular Engineering, University of Pennsylvania, Philadelphia, USA
[9] Carbon Direct, New York City, NY, USA
[10]Department of Chemical Engineering, Imperial College London, London, UK
[11]Wetsus, European Centre of Excellence for Sustainable Water Technology, Leeuwarden, the Netherlands
[12]Science Policy Research Unit (SPRU), University of Sussex Business School, United Kingdom
[13]Department of Earth and Environment, Boston University, United States
[14]Potsdam Institute for Climate Impact Research, Potsdam, Germany
[15]DWI – Leibniz-Institute for Interactive Materials, Aachen, Germany
[16]Climeworks AG, Zürich, Switzerland
[17]Priestley Centre for Climate Futures, University of Leeds, UK

Background

- Carbon dioxide removal (CDR) technologies play an important role in meeting global temperature targets
- Yet, research on individual carbon dioxide removal (CDR) technologies varies
- A harmonized review protocol across CDR pathways is needed to provide information on an equal footing
- This is especially true for direct air capture (DAC) where we have witnessed substantial efforts in materials design, but very limited research on technologies, systems, costs, environmental impacts, socioeconomic and policy.
- Most studies applying global Integrated Assessment Models (IAMs) do not include the full range of CDR technologies available, including DACCS, due to limited and fragmented information
- Previous reviews provide a high-level overview of CDR technologies and literature, but lack the granularity necessary for informing IAM scenarios, implementation, and policy making. (1–3)
- A detailed overview of the current evidence on individual CDR technologies, including DACCS, is necessary to advancing learning on CDR
- This requires coordinated research synthesis efforts by the research community

Objectives

This review is part of a larger initiative that aims to facilitate individual, coordinated systematic reviews for all CDR technologies based on a systematic map. Here, we focus on DACCS. In undertaking these systematic reviews, our goal is to develop a comprehensive review ecosystem within the research community to help us (1) understand the status and evolution of individual CDR technologies, including trends and research gaps, (2) inform future research, and (3) support policymakers, decision-makers, and investors in accelerating CDR development and deployment.

Primary question: What is the *current state* of knowledge and *evidence base* on DACCS in published research?

Secondary research questions

- Where do we have literature on DACCS?
- How much literature is available on different types of DACCS?
- What information is available on DACCS projects from specific developers?
- What do we know about DACCS design, performance, and economic considerations?
- What is the status of DACCS deployment and delivery?
- What do we know about possible environmental side effects of DACCS?

- How important are geographic factors when siting a DACCS plant?
- What may be the socioeconomic impacts and how does the policy/governance landscape look?
- How far are we developing robust DACCS MRV protocols?
- How does DAC fit in future decarbonization scenarios and what does this mean for DACCS upscaling?

Defining DACCS

Direct air CO_2 capture and storage (DACCS) is a chemical process by which CO2 is captured directly from the ambient air, with subsequent safe and permanent storage. It is also known as direct air capture and storage (DACS).

Stakeholder engagement

An expert review team consisting of experts from the DACCS field was responsible for the development of the systematic review protocol on the topic. Specifically, the expert review team decides on the final research questions, coding categories, data collection approach, and type of data synthesis. The review team is also responsible for leading and implementing the systematic review.

Methods

This protocol provides guidance for conducting a systematic review from a systematic map. A systematic map protocol was first designed and implemented by researchers at the Potsdam Institute for Climate Impacts Research (PIK) to understand the current state and development over time of published research on carbon dioxide removal technologies. For each CDR technology outlined in the systematic map, a specific systematic review protocol is iteratively designed by a technology-specific expert review team based on a template provided by PIK. The expert review team then also implements the systematic reviews for their respective technology.

This document presents the systematic review protocol template adapted for the review of the CDR technology Direct Air CO_2 Capture and Storage. We start by introducing the method used for generating the corpus of review literature on DACCS, adapted from the method used in the systematic map protocol. Specific steps include the literature search strategy, screening, and coding labels. Next, we outline the data extraction (i.e., coding) methods for the systematic review. The data extracted in the review expands on the initial coding undertaken for the map and goes into more granular detail with new coding categories and labels and by disaggregating some of the existing systematic map coding labels.

The systematic review broadly follows the guidance for systematic reviews developed by the Collaboration for Environmental Evidence (CEE, https://environmentalevidence.org/information-for-authors/), and the protocol conforms to the ROSES standards for reporting for systematic review protocols (https://www.roses-reporting.com/).

Search strategy

Sources

To ensure our review as comprehensive as possible, we include ten bibliographic databases and one platform in our literature search. These are outlined in Table 1.

Table 1. *Bibliographic databases (including platforms) included in search*

Database(s)	Platform
Science Citation Index Expanded (SCI-EXPANDED) 1900 -present;<br>Social Sciences Citation Index (SSCI) 1900-present;<br>Arts & Humanities Citation Index (A&HCI) 1975-present;<br>Conference Proceedings Citation Index- Science (CPCI-S) 1990-present;<br>Emerging Sources Citation Index (ESCI) -- 2015-present;<br>Conference Proceedings Citation Index- Social Science & Humanities (CPCI-SSH) 1990-present;<br>Book Citation Index– Science (BKCI-S) -- 2005-present;<br>Book Citation Index– Social Sciences & Humanities (BKCI-SSH) -- 2005-present;<br>Current Chemical Reactions (CCR-Expanded) -- 1985-present;<br>Index Chemicus (IC) -- 1993-present	Web of Science Core Collections

Search string

To capture the research on DACCS, we use a search string that includes relevant terminology that can be used to identify literature on the technology. We consciously refrain from the use of abbreviations in our search string to avoid the introduction of additional noise.

During the generation of the query, random samples of records are screened on a title and abstract basis. Exclusion statements are developed accordingly to reduce noise by excluding records that use related terminology in a field or topical focus with no relation to DACCS.

Table 2: Keywords in our search string for DACCS
Syntax is given for Web of Science Core Collection.

Subject	Associated keywords
	DACCS
DACCS	"TS = (((captur* OR extract) AND (direct* NEAR/3 (air OR atmosph*))) AND (CO2 OR carbon)) OR TS = ((*sorbent OR amine OR membrane) AND capture NEAR/2 (carbon OR CO2) AND (""ambient air"" OR ""atmospher*"")) OR<br>AB = ((reduc* OR captur* OR stor* OR extract* OR remov*)NEAR/2 ("CO2" OR "carbon") NEAR/2 ("ambient air" OR "direct air"))<br><br>NOT TS = (phenolic OR PCB* OR particulate OR NOx OR isotope OR "heat pump" OR polycyclic OR *bacteria* OR lignin OR sink OR pollution OR biofuel* OR sugar)

Languages
We limit our search to literature that is available in English. While it could be useful to capture literature in other languages in the future, the corpus of English literature is already large. Adding additional languages to the search would increase the time, capacities, and resources needed to undertake the review. As such, conducting this first review in only English can already give an indication of the state of the research while providing a starting point for potential replication in other languages in the future.

Checking comprehensiveness of the search
To ensure comprehensiveness, the results from the search query are checked using a validation set – a collection of records of known validity and importance.

Grey literature
Our initial database does not include grey literature. While it is likely that there is some research on DACCS in such literature, it is more difficult to access in a systematic manner. Additionally, the lack of a comprehensive database of grey literature also would make its inclusion more complex and time intensive. However, where grey literature sources added relevant complementary information, and had undergone some sort of peer review, individual reports were manually added to the review literature.

Combining records and removing duplicates
Search results are scraped from the respective databases and combined in the bespoke review management tool (apsis.mcc-berlin.net), for screening.

Screening for eligibility

Screening process

Once the dataset of articles on DACCS is collected, the documents are screened at the title and abstract level by a team of trained coders at PIK using the review management tool mentioned in the previous section. To be considered relevant, the documents must meet the inclusion criteria presented in the subsequent section. The coding is carried out in duplets to ensure consistency and any discrepancies that cannot be resolved between the two coders are discussed and clarified in the full group. Discrepancies in inclusion decisions are discussed, inclusion criteria revised and clarified where necessary.

Consistency checking

Coding is conducted by multiple researchers from different backgrounds. To ensure that their coding is consistent, all of the extracted data is stored in a database developed as per labels below, and all entries are checked by the lead author. This allows us to find disagreements between researchers; such disagreements are discussed with the wider team until consensus is reached.

Study coding and meta-data extraction

We extract a range of meta-data from the relevant studies (see Table 3) and apply codes to relevant studies, to further characterize the research. The coding schema for our categories is described in Tables 4-12.

Meta-data

Data in this category provides a high-level description of each study.

Table 3. *Coding scheme for the meta data extraction.*

Label /	Description/Definition
Citation	First author name, last author name, publication date, title, doi
Lead author affiliation	The full address of the lead author (organization, street, city, country)

Geographical information

This category captures information on the location or geographic focus of a study, if applicable. If multiple study locations or sites or covered, please include all.

Table 4. *Coding scheme for the geographical information category on the publication.*

Label /	Description/Definition

World region	Where in the world is the lead institution from
Country	Where in the world is the lead institution from
City	Where in a country is the lead institution from

Types of DAC

This label identifies the type of DAC technology that is the focus of a study. If the study does not research a specific DAC technology.

Table 5. *Coding scheme for the DAC types category*

Label /	Description/Definition
Liquid solvent with mineral looping	Technologies using some kind of mineral looping scheme to regenerate the solvent
Solid adsorbent	Technologies using solid adsorbents to capture and produce a pure CO_2 stream. Excludes mineral looping solid sorbents.
Membrane-based	Technologies using physical separation of CO_2 via pressure differentials over CO_2 selective membranes.
Cryogenic DAC	Technologies producing 'dry ice' CO_2 by sublimation of CO_2 onto solid surfaces.
Mineral looping DAC	Technologies using some kind of minerals (e.g., $Ca(OH)_2$ to capture CO_2, purified CO_2 is released upon heating of the mineral (calcination)
Amine-based solvents	Technologies using some kind of amine or amino acid (salt) to capture the CO_2, regeneration commences via heating, or preconcentration and heating.
Electrochemical-adsorbent	Technologies using electrochemistry to capture and concentrate CO_2, active phase is solid.
Electrochemical-solvent	Technologies using electrochemistry to capture and concentrate CO_2, active phase is liquid.

Design considerations

The category is used to code information that is relevant to the design of a DAC technology, such as any (non-financial) inputs or other factors that may influence CO2 uptake.

Table 6. *Coding scheme for the design considerations category*

Label	Description/Definition
Main capture material	adsorbent/absorbent/membrane type
Air contacting mechanism	Passive, active, fixed bed, moving bed, column, cooling tower
Main regeneration mechanism	Mineral looping, temperature swing, electrical swing, pH swing, pressure swing, humidity swing
CO_2 produced purity (%)	
CO_2 produced pressure (bar)	At which pressure is the CO2 produced for subsequent use or compression to transportation specs
Thermal energy requirement (GJ per tCO2) and regeneration temperature (Celsius)	
Electricity requirement (GJ per tCO2)	
Equivalent work consumption (kWh/GJ per tCO2)	
Main material inputs	Solvent (which), sorbent (which), minerals (which), process water, cooling water, other feedstock
Main waste streams	Mineral fines, spent sorbent/solvent, wastewater, amine (degradation) emissions to air

Project developer information

This category is used if a study provides information on DACCS projects that are being planned or implemented by specific developers. If information on multiple developers is included in a study, please record each project separately.

Table 7. *Coding scheme for the DAC developer category*

Label /	Description/Definition
Developing company	
Location	e.g., Country
Size	Nameplate capacity in tonnes CO_2 per annum
Year of start-up	

Deployment/delivery

This category captures information on the amount of DAC that has actually been deployed (project activities have begun) and delivered (carbon credits have been retired) and at which Technology Readiness Level technologies are.

Table 8. *Deployment/delivery*

Label /	Description/Definition
Current scale (tCO2 per year)	
Current TRL	using the US DOE definition
Current CO_2 sales price (US$/tonne removed)	If available/known
Foreseen commercial plant scale (kt CO2 captured per annum)	
Scale up time (unit)	Estimated time needed for scaling
Amount pilot/demonstration plants built	
Amount commercial plants built	

Environmental side effects

This category codes any potential or observed life cycle environmental effect of DACCS application or deployment on the (ecological/physical) environment, including life cycle assessment indicators.

Table 9. *Environmental side effects*

Label /	Description/Definition
LCA method used	E.g., ISO 14040/14044
LCA software used	Gabi, SimaPro, OpenLCA, etc
LCA database used	e.g., EcoInvent
LCA level	Country, region, global
LC environmental impact amount included	Number
LC environmental impact assessment method	Environmental Footprint, IPCC, ILCD, ReCiPe
System Boundaries/scope	
Functional Unit	
LCA - Climate change	
LCA – Ozone depletion	
LCA – Particulate Matter Formation	
LCA – Metal depletion	
LCA – Fossil resources depletion	
LCA – Eutrophication freshwater	
LCA – Eutrophication Marine	
LCA – Eutrophication Terrestial	
LCA – Acidification – terrestrial and freshwater	
LCA – Ionizing radiation	
LCA – Photochemical Ozone Formation	
LCA – Human Toxicity Cancer	
LCA – Human Toxicity Non-Cancer	
LCA – Ecotoxicity freshwater	
LCA – Land Use	
LCA – Water Scarcity	
LCA – Resource Depletion Energy	
LCA – Resource Depletion Minerals and Metals	

Economic considerations

This category is used to code detailed information about the economic viability of DACCS and its cost.

Table 10. Coding scheme for the economic considerations category

Label /	Description/Definition
Base location	
Base year	
Maturity	First of a kind, N^{th} of a kind, early mover
Cost basis	Net CO_2 removed or Gross CO_2 captured
Assumed project life (year)	
Study scale (tonne CO_2 per annum)	
Discount rate assumed (%)	Weighted average cost of capital (WACC)
Capacity factor (%)	Or hours per full year
Capital cost (USD/t CO_2 captured)	
Fixed operational cost (USD/t CO_2 captured)	
Variable operational cost (USD/t CO_2 captured)	
Current cost of capture (USD per tonne CO_2 gross/net removed)	
Projected cost of capture (USD per tonne CO_2 gross/net removed)	
Estimated capture price (USD per tonne CO_2 gross/net removed)	

Geospatial performance differences

This category is used to code detailed information about the spatial performance differences of DACCS.

Table 11. Coding scheme for the spatial considerations category

Label /	Description/Definition
Region	
Energy consumption (GJ/kWh per tonne CO_2 captured	Or differential to standard conditions
Productivity	Or differential to standard conditions
CO_2 removal efficiency	Considering differences in energy supply
Water consumption (tonne per tonne CO_2 captured/removed)	Or differential to standard conditions
Political considerations	E.g., established policy/incentive schemes

Socioeconomic side effects and policy/governance considerations
This category codes any potential or observed non-CDR effect of DACCS application or deployment on the social/economic surroundings plus considerations for policy and governance.

Table 12. *Non-environmental side effects*

Label /	Description/Definition
Social	Environmental justice, do no significant harm, etc.
Economic	Local and country GDP, wealth creation and equality.
Health	Impact on local community health
Jobs creation	How many jobs were/can be created?
Potential spillover effects	Wider technological advancement. I.e., creating a market for CO_2 transport and storage (should help CCS in general), creating large market for heat pumps and renewable energy etc.

References

1. Minx JC, Lamb WF, Callaghan MW, Fuss S, Hilaire J, Creutzig F, et al. Negative

emissions - Part 1: Research landscape and synthesis. Environ Res Lett. 2018;13(6).

2. Fuss S, Lamb WF, Callaghan MW, Hilaire J, Creutzig F, Amann T, et al. Negative emissions - Part 2: Costs, potentials and side effects. Environ Res Lett. 2018;13(6):063002.
3. Nemet GF, Callaghan MW, Creutzig F, Fuss S, Hartmann J, Hilaire J, et al. Negative emissions - Part 3: Innovation and upscaling. Environ Res Lett. 2018;13(6).
